# Diverging perspectives on emerging mental health symptoms: Multi‐informant discrepancies and their associated determinants across the transition to adolescence in the Adolescent Brain Cognitive Development Study

**DOI:** 10.1002/jcv2.70122

**Published:** 2026-05-24

**Authors:** Matias Martinez, Vijay A. Mittal, Jonathan D. Schaefer, Claudia M. Haase, Katherine S. F. Damme

**Affiliations:** ^1^ Department of Psychology Vanderbilt University Nashville Tennesse USA; ^2^ Institute for Innovations in Developmental Sciences Northwestern University Chicago Illinois USA; ^3^ Institute for Policy Research Northwestern University Evanston Illinois USA; ^4^ Department of Psychology Northwestern University Evanston Illinois USA; ^5^ Department of Psychiatry Northwestern University Chicago Illinois USA; ^6^ School of Education and Social Policy Northwestern University Evanston Illinois USA; ^7^ Interdepartmental Neuroscience Northwestern University Evanston Illinois USA; ^8^ Buffett Institute for Global Studies Northwestern University Evanston Illinois USA; ^9^ Department of Psychology University of Texas at Dallas Richardson Texas USA; ^10^ Center for Vital Longevity University of Texas at Dallas Dallas Texas USA; ^11^ Center for Brain Health University of Texas at Dallas Dallas Texas USA

**Keywords:** caregiver‐youth dyads, developmental change, mental health, reporter discrepancy, socio‐experiential environments

## Abstract

**Background:**

The transition from childhood to adolescence is marked by significant developmental, social, and contextual changes that present practical challenges for the measurement of emerging mental health symptoms. This study explores transdiagnostic symptoms across three core dimensions from the Achenbach system of empirically based assessment brief problem monitor—internalizing, inattention (INA), and externalizing (EXT) (aggressive/rule‐breaking behavior)—as well as across reporters (youth, caregiver, teacher), reporter characteristics, and developmental and social contexts.

**Methods:**

Four years of longitudinal data from the Adolescent Brain Cognitive Development (ABCD^®^) release 5.1 study sample included 11,832 youth (age: 9.67–14.75 years; 48% girls) from 21 sites across the United States. Youth symptoms of internalizing, INA, and EXT problems were reported by youth, caregivers, and teachers. Analyses examined mean‐level directional discrepancies in the reporters' overall ratings and whether those discrepancies were predicted by reporters' characteristics and socio‐experiential environments (e.g., parental warmth, school environment), and developmental change over time.

**Results:**

Discrepancies in youth symptom ratings varied systematically across reporters, youth and caregiver characteristics, and socio‐experiential factors. Overall, youth consistently rated their symptoms higher than caregivers and teachers across all domains, with discrepancies widening over time. Reporter discrepancies were greater if the youth was female, non‐white, or showed advanced pubertal development. Symptom ratings were higher from caregivers with higher education and depression symptoms. Teachers reported higher symptoms for youth with non‐white caregivers, but lower symptoms for youth with immigrant caregivers. Socio‐experiential factors show that caregiver reports of youth symptoms were higher than self‐reports in contexts of greater caregiver warmth and more prosocial school environments. Self‐reports were also higher than both caregivers and teacher reports of youth symptoms when experiencing higher levels of family conflict.

**Conclusion:**

When capturing youth symptoms, whom you ask matters. The impact of reporters varies in complex ways, including by features of the reporter, environment, developmental stage, and symptom dimension.

## Key Points

**What’s known?**

- Reports of youth mental health from different informants (youth, caregivers, teachers) often show discrepancies. However, less is known about their systematic relationship with symptom types, reporter characteristics, and social contexts.

**What’s new?**

- Youth self‐reports were typically higher than adults, a discrepancy that widens as they age, especially for inattention. These gaps are systematically associated with youth characteristics (e.g., being female), caregiver factors (e.g., depression), and social environments (e.g., family conflict).

**What’s relevant?**

- Whom you ask about a youth's mental health profoundly matters. Reporter perspectives are not interchangeable. This choice can significantly impact research findings and is critical for the accurate and early detection of emerging mental health problems.

## INTRODUCTION

Accurately detecting emerging symptoms of psychopathology in childhood poses a key methodological challenge for researchers: which symptom reports to rely on (De Los Reyes et al., 2023; Hope et al., 1999; Lewis et al., 2012; Pavlova & Uher, 2020). Early‐to‐middle childhood assessments of mental health rely on outside observers, including caregivers and teachers, while assessments of adolescents and adults rely on self‐report (Baldwin et al., 2024; De Los Reyes & Kazdin, 2005; Hope et al., 1999; Penney & Skilling, 2012). When the information is available from multiple reporters, the reports are often treated as interchangeable across observers, or concordant reports (De Los Reyes et al., 2023). Yet reality is complex. Adolescence is a dynamic period of development, with growing independence from adults, which may limit adults' insights into youth's behaviors (Dahl et al., 2018; Nelson et al., 2005). In this context, researchers must balance questions of developmental appropriateness of the reporter, potential biases of observers, and features of the context that influence the emerging symptoms (Charamut et al., 2022; De Los Reyes et al., 2023; Makol et al., 2020; Rezeppa et al., 2021).

In late childhood and early adolescence, emerging clinical symptoms tend to be subtle, heterogeneous, and often characterized by general distress rather than highly specific disorder features (McGorry & Nelson, 2016; Shah et al., 2022). As a result, early detection of symptoms may require integrating information across multiple symptom dimensions and reporters, as each informant provides a unique perspective. Some research has focused on convergent reports as a measure of validity while discounting discrepancies (i.e., differences among these reporters in rating the same youth's symptoms) as reflecting measurement error, reporter bias, or random error (e.g., Angold et al., 1987; Grace, 2001). However, a growing body of work suggests that informant discrepancies can also represent meaningful variation in youth behavior across development, settings, and relationships, thereby constituting domain‐relevant information rather than mere nuisance (De Los Reyes et al., 2013, 2023; De Los Reyes & Kazdin, 2005).

Indeed, reporter characteristics can lead them to misrepresent youth's behaviors and symptoms. For instance, the *depression‐distortion hypothesis* suggests that caregivers experiencing higher depressive symptoms perceive their children's mental health more negatively due to biases in their attention, memory, and interpretation of life events (e.g., Gartstein et al., 2009; Richters, 1992). Consistent with this view, Ehrlich et al. (2011, 2014) demonstrated that higher levels of maternal depressive symptoms were associated with greater discrepancies between mothers' and youth's reports of the youth's internalizing (INT) symptoms and relationship quality. Similarly, López‐Pérez & Wilson (2015) documented that caregivers' own emotional states predicted their perceptions of their youth's emotional well‐being, with caregivers' ratings of youth's happiness correlated more strongly with their own happiness than children's self‐reports. These findings underscore that discrepancies can emerge from systematic variation in who is doing the reporting, rather than differences in youths' underlying symptomatology.

Conversely, other researchers have linked informant discrepancies to variation in youths' underlying mental health and to differences in behavior across contexts, suggesting that disagreements can carry clinically meaningful information (De Los Reyes et al., 2013, 2023). For example, differences in symptoms reported by caregivers observing youth's behaviors at home and by teachers observing youth's interactions with other peers at school may reflect important dynamics within the family or school (e.g., DuPaul et al., 2020; Olson et al., 2018; Stone et al., 2013). Recent work has also shown that discrepancies can improve prediction of clinically relevant outcomes when they are modeled explicitly rather than treated as noise. Makol et al. (2019) found that when caregivers reported greater INT symptoms than adolescents' self‐reports, youths were more likely to exhibit suicidality upon admission to inpatient psychiatric care and to receive intensive treatment during their hospital stay. Similarly, Cohen et al. (2019) demonstrated that while youth self‐reports were more accurate than caregivers' ratings in detecting *concurrent* depressive episodes relative to clinical diagnostic interviews, caregiver reports were more accurate in forecasting *future* episodes.

Collectively, the available evidence highlights that discrepancies may reflect meaningful variability in social contexts or situational specificity, as well as observers' perspectives or potential biases. This blended interpretation likely characterizes many predictors of informant discrepancies (e.g., Ehrlich et al., 2011). For example, discrepancies around immigrant families may reflect both youth's behavioral differences and reporter's perceptual biases. The *acculturation discrepancy hypothesis* proposes that when youth acculturate faster to the host culture than their parents and contribute to genuine mental health symptoms (Kim et al., 2013; Schwartz et al., 2016). At the same time, immigrant caregivers' expectations, parenting practices, and relationships often differ from those in their host countries, generating disagreements in perceptions of normative youth behavior that may be unrelated to mental health symptoms (Kim et al., 2016; Lau et al., 2004; Verhulp et al., 2019).

Demographic characteristics of youths and reporters provide another illustration of this dual role of discrepancies as both potential bias and signal. Features of reporters and their social environments may contribute to symptom discrepancies and, at the same time, serve as markers for how youths are perceived and treated in their social worlds. For example, Barrett & DuPaul (2018) found that mother's racial background predicted attention deficit hyperactivity disorder (ADHD) ratings more than child's racial background. Relatedly, DuPaul et al. (2020) identified caregiver–teacher discrepancies in ADHD symptom reports that varied by youth gender, age, race, and ethnicity. These patterns may reflect systematic biases in expectations of youth based on demographic characteristics, but they may also add clinically relevant information about how youths' identities are perceived and responded to across home and school contexts.

In addition to these cross‐sectional determinants of informant discrepancies, an emerging literature has begun to ask how discrepancies themselves change as children move into adolescence. The transition to adolescence is marked by a growing independence from family and other authority figures (e.g., caregivers, teachers) and a shift toward a peer‐centered social system, as described by social reorientation model (Nelson et al., 2005). This normative social transition may lead to increasing discrepancies between youth and caregiver reports, if youth's daily experiences and internal states become less visible to adults (Nelson et al., 2016). Alternatively, as youth mature, they may come to evaluate their own symptoms in ways that more closely resemble how adults view them, potentially reducing discrepancies over time (Muris et al., 2004; Patalay et al., 2014). Most empirical work on informant discrepancies has focused on cross‐sectional data (De Los Reyes et al., 2015), with relatively fewer studies examining longitudinal associations and even fewer centering on this developmental period (Parker et al., 2025; Yang et al., 2021). Among those taking a longitudinal approach, Yang et al. (2021) observed that caregiver‐youth reports of aggressive behavior tend to diverge over time, whereas Parker et al. (2025), using the same dataset as the present study, reported relatively low agreement between caregiver‐ and youth‐reported suicidal ideation. These single‐domain findings are consistent with the idea that social reorientation in adolescence may reshape reporter agreement, but they do not yet clarify whether similar patterns hold across symptom dimensions.

The present study seeks to extend the empirical literature on informant discrepancies by leveraging the large, longitudinal Adolescent Brain Cognitive Development (ABCD) Study®. We focus on youth ages 9–13 and integrate self‐, caregiver‐, and teacher‐reports of symptoms across three domains—internalizing, INA, and externalizing (EXT) (aggressive/rule‐breaking behavior)—alongside a broad set of youth, caregiver, and contextual characteristics. We specifically aim to address three research questions:

1. Do reporters disagree on youth symptoms, and does mean‐level disagreement vary by INT, INA, and EXT domains? Overall, the empirical literature on informant discrepancies has focused on specific mental health domains independently (Charamut et al., 2022; DuPaul et al., 2020; Hart et al., 2013; Makol et al., 2020; Parker et al., 2025; Robinson et al., 2019; Yang et al., 2021) with fewer studies examining multiple domains simultaneously (Bonadio et al., 2022; Makol et al., 2020; Stone et al., 2013). These latter studies have focused on comparing youth‐caregiver discrepancies across INT and EXT symptoms, with some suggesting slightly smaller discrepancies for EXT symptoms (De Los Reyes et al., 2015; Makol et al., 2020) and others showing no differences (Jungersen, 2023).
2. Are reporter characteristics and socio‐experiential environments associated with larger or smaller discrepancies? Following previous studies (e.g., DuPaul et al., 2020; Ehrlich et al., 2014; Kim et al., 2016), we test whether youth characteristics (i.e., age, sex, race, immigrant status), caregiver characteristics (i.e., sex, race, immigrant status, depressive symptoms, education), and social‐experiential contexts (i.e., caregiver warmth, prosocial school climate, family conflict, neighborhood deprivation) are associated with informant discrepancies.
3. Do discrepancies change as youth transition into early adolescence? Building on recent studies (e.g., Parker et al., 2025; Yang et al., 2021), we ask whether discrepancies remain stable or change across the transition from late childhood to early adolescence.

## METHODS

### Participants

The ABCD study recruited a sample of 11,832 youths and their primary caregivers in 21 cities in the US (Garavan et al., 2018; Karcher & Barch, 2020). The present analyses are based on data from baseline through the 3‐year follow‐up and use only complete cases with list‐wise deletion.

### Ethical considerations

Children verbally consented to the study, their caregivers provided written informed consent, and each participating research site’s Institutional Review Board (IRB) certified that the study complied with the biomedical ethics requirements for research with human subjects (Clark et al., 2018). ABCD protocol was approved by the central IRB at UC San Diego and by local IRBs at some sites. Additional details and reliability measures for the main variables at baseline are reported in Supporting Information S1: Appendix S1; Table S1.

### Measures

#### Youth mental health

*Self‐reports* were measured using the brief problems monitor (BPM‐Y). The BPM‐Y is an abbreviated version of the child behavior checklist (CBCL) including 19 items measuring three subscales: six INT symptoms (e.g., feels worthless; too fearful or anxious), six symptoms of INA (e.g., can’t concentrate for long; restless or hyperactive), and seven EXT symptoms (e.g., argues a lot; threatens people). The extra EXT item on the youth version originates in questions about disobedient behavior at home and school (Karcher & Barch, 2020; Pedersen et al., 2021; Piper et al., 2014). The instrument is administered every 6 months. The response options range from 0 to 2 (0 = not true, 1 = somewhat true, or 2 = very true).

*Caregiver reports* of youth mental health symptoms were measured using the CBCL, a 119‐item questionnaire completed by caregivers that assess their child's behavioral, social, and emotional problems (Achenbach & Rescorla, 2001; Michelini et al., 2019). To align with the BPM‐Y measures, we constructed three CBCL subscales corresponding to INT, INA, and EXT symptoms using the 18 items that are common across informants. The CBCL is administered annually starting at baseline.

*Teacher reports* of youth symptoms were collected using the brief problem monitor–teacher (BPM‐T), the teacher‐report counterpart to the BPM‐Y derived from the Teacher's Report Form (Achenbach & Rescorla, 2001). The BPM‐T includes 18 items, with six items each assessing INT, INA, and EXT problems, rated on the same 0–2 response scale as the BPM‐Y (0 = not true, 1 = somewhat or sometimes true, 2 = very true or often true). Teachers were recruited via youth caregivers identified whom they had the most frequent contact with their child. ABCD staff then asked the selected teacher asking to complete the BPM‐T (Barch et al., 2021). 70% of youth had at least one non‐missing teacher BPM‐T report. Teacher characteristics are not available.

We used symptom scores from the waves with reports from youth, caregiver, and teachers, and constructed parallel INT, INA, and EXT scales based on the 18 shared items, for different clinical magnitudes see Supporting Information S1: Tables S2–S4; Appendix S2, and symptom‐level data, see Supporting Information S1: Table S5; Figure S1; Appendix S3. When comparing youth‐caregiver reports, the self‐report of disobedience at school was excluded, and when comparing caregiver‐teacher reports, we compared reports of disobedience at home for caregivers and at school for teachers.

#### Youth characteristics

Demographic features: Included children's age (in years; between 1‐ and 3‐year follow up: mean [*M*_1–3_] = 11.93, standard deviation [SD_1–3_] = 1.04; at baseline: mean [*M*_0_] = 9.91, standard deviation [SD_0_] = 0.62), sex determined at birth is girl (Baseline: 47.79%; 3 time‐points: 47.54%), racial background is non‐white (Baseline: 47.96%; 3 time‐points: 46.39%), and youth was born outside the United States (U.S.) (Baseline: 2.95%; 3 time‐points: 2.88%).

Pubertal Development: Pubertal status at baseline was assessed with the pubertal development scale (PDS; Petersen et al., 1988), as adapted by Cheng et al. (2021). Youth completed a self‐report version that asks about visible physical changes associated with puberty (e.g., body hair, skin changes) as well as sex‐specific changes (e.g., breast development girls; facial hair growth boys). Items are rated on a 4‐point scale from 1 (“has not yet begun”) to 4 (“seems complete”), with menarche recorded as a yes/no item. Following ABCD conventions, a continuous youth‐reported puberty score by averaging the relevant PDS items, with higher scores indicating advanced pubertal development (*M*_0_ = 1.67, SD_0_ = 0.52).

#### Caregiver characteristics

Demographic features: Included primary caregivers' age (in years; between 1‐ and 3‐year follow up: *M*_1–3_ = 42.13, SD_1–3_ = 6.85; at baseline: *M*_0_ = 39.95, SD_0_ = 6.84), sex at birth is female (Baseline: 89.03%; 3 Time‐points: 89.06%), racial background is non‐white (Baseline: 25.70%; 3 Time‐points: 24.25%), and caregiver was born outside the United States (Baseline: 17.60%; 3 Time‐points: 17.43%), and educational attainment (e.g., holds a master's degree or doctorate: Baseline: 25.21%; 3 Time‐points: 26.19%).

*Depressive symptoms* of the caregiver were measured at baseline using Achenbach adult self‐report, which is one component of the Achenbach System of Empirically Based Assessment (Achenbach & Rescorla, 2003; Guerrero et al., 2020; Michelini et al., 2019). Caregivers reported 14 symptoms of depression in the past six months (e.g., lack of energy, cries, suicidal thoughts, feeling worthless; 0 = Not true; 1 = Somewhat or sometimes true; 2 = Very true or often true; *α*_0_ = 0.84; averaged: *M*_0_ = 3.97; SD_0_ = 3.66).

#### Socio‐experiential environments

*Parental warmth* was measured using the Acceptance Subscale of the Child Report of Behavior Inventory (Kerr et al., 2021; Zucker et al., 2018). Youth reported on caregiver warmth and acceptance of their primary caregiver using 5 items (e.g., makes me feel better after talking over my worries with him/her; 1 = Not like him/her; 2 = Somewhat like him/her; 3 = A lot like him/her; *α*_0_ = 0.79; averaged: *M*_0_ = 2.78; SD_0_ = 0.30).

*School prosocial environment* was measured using 6 items from the Inventory for School Risk and Protective Factor included in the ABCD study (Arthur et al., 2007; Zucker et al., 2018). Youth reported on opportunities and rewards for prosocial involvement in their schools (e.g., “In my school, students have lots of chances for students to get involved in sports, clubs, or other activities outside of class,” “In my school, students have lots of chances to help decide things like class activities and rules”; 1 = NO!; 2 = no; 3 = yes; 4 = YES!—with capitalized responses meaning that the statement is definitely true for the child, and little letter if it is mostly true for them; *α*_0_ = 0.61; averaged: *M*_0_ = 3.32; SD_0_ = 0.47).

*Family conflict* was measured at baseline using the Family Conflict subscale of the Family Environment Scale of the PhenX toolkit (Assari et al., 2021; Hendershot et al., 2015). Specifically, youth reported on fighting, anger, criticism, competitiveness, and yelling within the family using 9 items (0 = No, 1 = Yes; *α*_0_ = 0.70; averaged: *M*_0_ = 0.23; SD_0_ = 0.22).

*Neighborhood deprivation* was measured by an area deprivation index ranging from 1 to 100 and validated for the United States (U.S.) by Singh (Kind et al., 2014; Singh, 2003), which includes 17 U.S. census poverty, education, housing, and employment indicators (*M*_0_ = 0.95; SD_0_ = 0.21).

Table 1 presents the descriptive statistics of variables measured between 1‐ and 3‐year follow ups for the 11,832 youths (5655 females, 6177 males) in the sample, and Supporting Information S1: Table S1 in Appendix S1 presents the descriptive statistics measured at baseline.

**TABLE 1:** Summary statistics, measured between 1‐ and ‐3‐year follow‐ups.

	Range	*N*	Mean	SD
	(1)	(2)	(3)	(4)
Youth mental health symptoms				
Internalizing symptoms, youth reports	0–12	31,063	1.86	2.24
Internalizing symptoms, caregiver reports	0–12	32,195	1.46	1.90
Internalizing symptoms, teacher reports	0–12	12,407	1.54	2.15
Inattention symptoms, youth reports	0–12	30,040	3.36	2.71
Inattention symptoms, caregiver reports	0–12	32,196	2.20	2.61
Inattention symptoms, teacher reports	0–12	12,462	2.41	3.00
Externalizing symptoms, youth‐caregiver reports	0–12	30,863	1.97	1.90
Externalizing symptoms, caregiver reports	0–12	32,195	1.58	1.98
Externalizing symptoms, youth‐teacher reports	0–12	30,903	1.84	1.79
Externalizing symptoms, teacher reports	0–12	12,465	0.94	2.00
Youth characteristics				
Age (in years)	9.67–14.75	32,526	11.93	1.04
Sex at birth (girl = 1)	0–1	32,457	47.54%	0.50
Race (non‐white = 1)	0–1	32,453	46.39%	0.50
Pubertal development, at baseline	1–4	32,268	1.67	0.52
Immigrant (born outside US = 1)	0–1	32,419	2.88%	0.17
Caregiver characteristics				
Age (in years, at baseline)	23–80	32,236	40.10	6.75
Sex at birth (female = 1)	0–1	32,441	89.06%	0.31
Race (non‐white = 1)	0–1	32,394	24.25%	0.43
Immigrant (born outside US = 1)	0–1	32,449	17.43%	0.38
Depressive symptoms, at baseline	0–28	32,450	3.96	3.63
Primary caregiver’s education, at baseline				
High school or less	0–1	32,414	15.93%	0.37
Some college	0–1	32,414	16.01%	0.37
Associate degree	0–1	32,414	12.89%	0.34
College	0–1	32,414	28.98%	0.45
Masters or more	0–1	32,414	26.19%	0.44
Social environments, measured at baseline				
Parental warmth	1–3	32,389	2.78	0.30
Prosocial school environment	1–4	32,409	3.32	0.47
Family conflict	0–1	32,409	0.23	0.22
Neighborhood deprivation	0.01–1.26	30,182	0.94	0.21

*Note*: Range corresponds to observed range in the data.

Abbreviations: *N*, number of observations (pooled across the 1–3 years follow‐ups); SD, standard deviation.

### Analysis plan

#### Discrepancies by reporters and mental health domains

Paired *t*‐tests statistically test informant discrepancies for the three mental health domains of interest (i.e., INT, INA, and EXT), comparing ratings between each reporter dyad.

#### Predictors of discrepancies

We conducted separate regression analyses for each informant dyad (i.e., youth‐caregiver, youth‐teacher, caregiver‐teacher) and each mental health domain (INT, INA, and EXT), resulting in nine outcomes in total. The model predictors included five youth characteristics (i.e., age, sex at birth, racial background, pubertal development, and immigrant status), five caregivers' characteristics (i.e., age, sex at birth, racial background, immigrant status, and depressive symptoms), and four social environments (i.e., caregiver warmth, prosocial school environment, family conflict, and neighborhood deprivation). All predictors are measured at baseline except for youth's age, which is linked the longitudinal transitions seen longitudinally. To account for the hierarchical structure of the data, models included random intercepts for families and research sites grouping by individual. Analyses were performed using multilevel mixed‐effects linear regression models fitted by maximum likelihood estimation in Stata (command mixed; Heeringa & Berglund, 2020; StataCorp LP, 2013). For this analysis and ease of interpretation, we standardized non‐binary variables.

#### Reporters' discrepancies in the transition to adolescence

Multilevel mixed‐effects linear regression model (described above) examined how informant reports of youth mental health symptoms changed over time, focusing on overall symptoms and each specific mental health outcome (i.e., INT, INA, and EXT). Specifically, we transformed our original panel dataset, which included reports from multiple reporters (i.e., youth, caregivers, teachers) across three follow‐up periods (years 1‐ through 3, with baseline data included as covariates; *N* = 32,529 observations) into an expanded panel of mental health domains × reporters (expanding the original panel by nine times; *N* = 292,761 observations) over the same period. Additionally, each model included informant indicators (caregiver, teacher) and their interaction with youth age to explicitly test whether discrepancies between informants changed over time as youth transitioned into adolescence. Models accounted for clustering by families and research sites by including random intercepts and were fitted via maximum likelihood estimation in Stata, see Supporting Information S1: Appendix S4; Table S6.

#### Sensitivity analyses

Sensitivity analyses explored the robustness of results to corrections for alternative operationalizations of discrepancies (Supporting Information S1: Appendix S5; Tables S5–S13; Figures S2 and S3), missingness/multiple imputation (Supporting Information S1: Appendix S6; Tables S14–S18; Figure S4), multiple testing (Supporting Information S1: Appendix S7; Tables S19 and S20), different specifications for testing predictors of discrepancies (Supporting Information S1: Appendix S8; Tables S21–S26), and testing discrepancies over time (Supporting Information S1: Appendix S9; Table S27; Figure S5). Overall conclusions from the sensitivity analyses are available in Supporting Information S1: Appendix S10.

## RESULTS

### Discrepancies by reporters and domains

**FIGURE 1:**
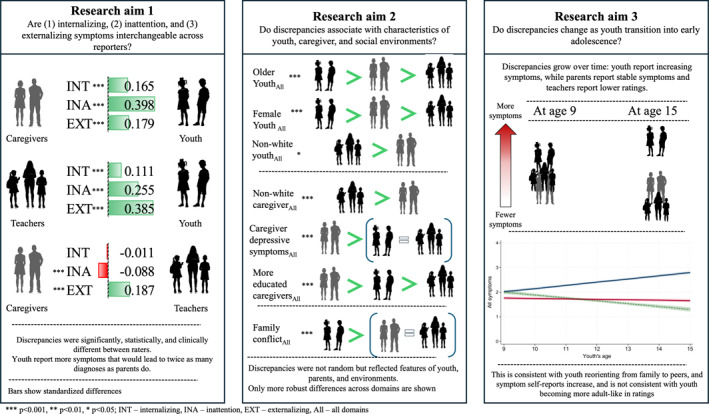
Overview of the primary aims and findings.

**FIGURE 2:**
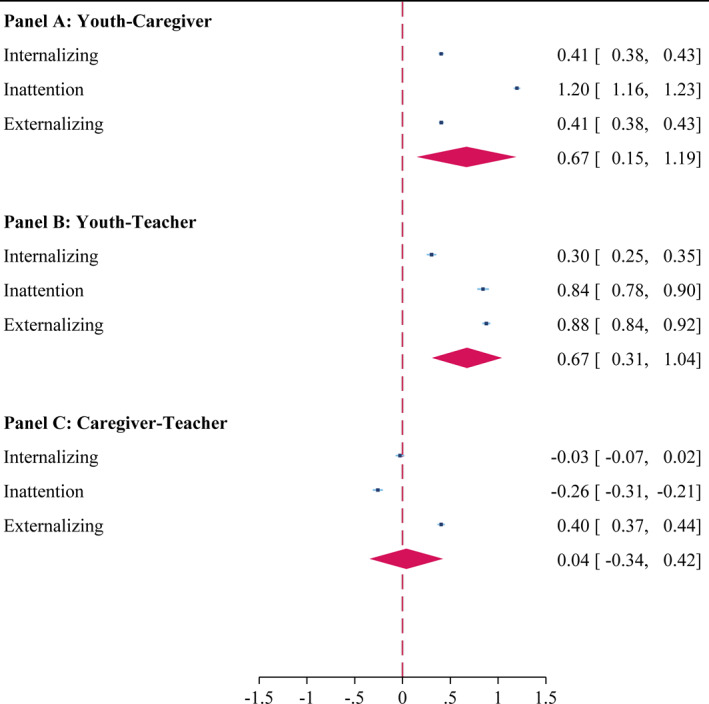
Mean‐level discrepancies by reporters and mental health domains. Point estimates are presented to the right and in dark blue squares. The 95% confidence intervals are presented in brackets to the right and in light blue lines. In Panel (A) and (B), positive values indicate higher youth self‐reports relative to caregivers and teachers, respectively. In Panel (C), positive values indicate higher reports from caregivers relative to teachers.

Paired *t*‐tests indicated significant discrepancies in youth mental health ratings between informants (youth‐caregiver, youth‐teacher, caregiver‐teacher) across three broad domains: INT, INA, and EXT symptoms (Table 2; Figures 1 and 2). Specifically, youths consistently reported higher levels of INT symptoms compared to caregivers (mean difference [MD] = 0.405, standardized mean difference [SMD] = 0.165, *p* < 0.001) and teachers (MD = 0.304, SMD = 0.111, *p* < 0.001). Conversely, caregivers reported slightly fewer INT symptoms than teachers (MD = −0.027, SMD = −0.011, *p* < 0.001). For INA symptoms, youths reported higher symptom levels compared to caregivers (MD = 1.198, SMD = 0.398, *p* < 0.001) and teachers (MD = 0.842, SMD = 0.255, *p* < 0.001), with caregivers reporting significantly lower levels than teachers (MD = −0.259, SMD = −0.088, *p* < 0.001). Regarding EXT symptoms, youth reports were again higher than those of caregivers (MD = 0.406, SMD = 0.179, *p* < 0.001) and substantially higher than teachers (MD = 0.877, SMD = 0.385, *p* < 0.001). Caregivers also reported higher levels of EXT symptoms compared to teachers (MD = 0.404, SMD = 0.187, *p* < 0.001).

**TABLE 2:** Mean‐level discrepancies, by reporters and symptoms.

	Differences in symptoms, by reporters		
	Youth—Caregiver	Youth—Teacher	Caregiver—Teacher
	(1)	(2)	(3)
Internalizing symptoms	0.405 [0.165]***	0.304 [0.111]***	−0.027 [−0.011]
Inattention symptoms	1.198 [0.398]***	0.842 [0.255]***	−0.259 [−0.088]***
Externalizing symptoms	0.406 [0.179]***	0.877 [0.385]***	0.404 [0.187]***

*Note*: Results based on the BPM‐Y, BPM‐T, and CBCL, which are available for all participants from baseline to the 3‐year follow‐up for the caregiver and teacher, and from the 1‐year follow‐up to the 3‐year follow‐up for the youth. We focused our analysis on data from 1‐ to 3‐year follow‐up. Columns (1–3) show the average difference between reporters and the statistical significance according to a paired *t*‐tests. Standardized differences in brackets. Differences in Column (1) are based on around 30,000 observations, and differences in reported in Columns (2) and (3) are based on around 12,000 observations ***p < 0.001, **p < 0.01, *p < 0.05, *t* < 0.10.

Abbreviations: BPM‐T, brief problem monitor–teacher; BPM‐Y, brief problems monitor; CBCL, child behavior checklist.

These discrepancies are not only statistically significant but also clinically meaningful. In a follow‐up analysis, we estimated that the prevalence of meeting “diagnoses” criteria can be twice as high (e.g., prevalence of 3.09% for the “diagnosis” of an INT disorder based on youth self‐reports compared to 1.51% for caregiver reports; and assuming a threshold of 8 in the 0–12 score) depending on who reports the information and how strict the thresholds are defined to identify a disorder (Supporting Information S1: Tables S2–S4 in Appendix S2).

#### Discrepancies by symptoms

To gain deeper insights into individual drivers of informant discrepancies, differences were examined at the symptom level (Supporting Information S1: Table S5 and Figure S1 in Appendix S3). Among symptoms with the highest discrepancies, “restless or hyperactive” demonstrated substantial differences, with youth reporting significantly higher symptoms compared to caregivers (mismatch rate [MMR] = 55%) and teachers (MMR = 56%). Similarly, for “argues a lot,” youth ratings were higher compared to teachers (MMR = 53%), and caregivers reported higher symptoms compared to teachers (MMR = 44%). Another prominent discrepancy appeared for the symptom “can't concentrate for long,” with youth reporting higher levels than caregivers (MMR = 49%) and teachers (MMR = 50%).

### Predictors of discrepancies

Mean‐level directional Analysis: Discrepancies reported in the previous sections across INT, INA, and EXT domains (Table 3) varied systematically based on youth characteristics, caregiver characteristics, and socio‐experiential environments. Among youth characteristics, older youth showed greater discrepancies, consistently reporting more symptoms than caregivers (*p*'s < 0.001) and teachers (*p*'s < 0.001), with smaller differences between caregiver and teacher ratings (*p*'s < 0.01). Compared to non‐females, female youth reported significantly higher levels of all symptom domains compared to both caregivers (*p*'s < 0.001) and teachers (*p*'s < 0.001). Non‐white youth generally showed slightly higher self‐reports relative to caregivers (*p*'s < 0.05) and lower ratings from teachers for EXT symptoms (*p* < 0.05) but not for other domains. The main discrepancies were between teachers and caregivers, with teachers reporting higher symptoms for non‐white youth than white youth (*p*'s < 0.001). Pubertal development was associated only with youth‐caregiver INT discrepancies such that youth reported higher symptoms than their caregivers (*p*'s < 0.001). Youth immigration status was negatively related to youth‐caregiver discrepancies in INA symptoms (*p* < 0.05) and positively to caregiver‐teacher discrepancies in EXT symptoms (*p* < 0.01).

**TABLE 3:** Predictors of mean‐level discrepancies, by reporters and domains.

	Youth—Caregiver			Youth—Teacher			Caregiver—Teacher		
	INT	INA	EXT	INT	INA	EXT	INT	INA	EXT
	(1)	(2)	(3)	(4)	(5)	(6)	(7)	(8)	(9)
Youth’s characteristics									
Age (in years)	0.132***	0.235***	0.106***	0.234***	0.309***	0.223***	0.082***	0.070**	0.119***
	(0.012)	(0.015)	(0.011)	(0.026)	(0.030)	(0.020)	(0.023)	(0.026)	(0.018)
Sex at birth (girl = 1)	0.518***	0.875***	0.378***	0.480***	1.461***	0.555***	0.098*t*	0.707***	0.260***
	(0.036)	(0.046)	(0.034)	(0.057)	(0.069)	(0.048)	(0.051)	(0.060)	(0.044)
Race (non‐white = 1)	0.136*	0.154*	0.06*	−0.048	−0.017	−0.156*	−0.242***	−0.200*	−0.212***
	(0.054)	(0.069)	(0.051)	(0.080)	(0.098)	(0.068)	(0.071)	(0.084)	(0.062)
Pubertal development	0.088***	−0.030	−0.011	0.064*	0.003	0.009	−0.027	0.018	−0.029
	(0.018)	(0.022)	(0.017)	(0.029)	(0.035)	(0.024)	(0.026)	(0.030)	(0.022)
Immigrant (born outside US = 1)	0.146	−0.361*	−0.156	−0.034	−0.243	0.242	0.032	0.303	0.398**
	(0.115)	(0.148)	(0.111)	(0.188)	(0.233)	(0.161)	(0.170)	(0.200)	(0.147)
Caregiver’s characteristics									
Age (in years)	−0.000	−0.008*t*	−0.005	−0.010*	−0.009	−0.001	−0.009*	0.000	−0.000
	(0.003)	(0.004)	(0.003)	(0.005)	(0.006)	(0.004)	(0.004)	(0.005)	(0.004)
Sex at birth (female = 1)	−0.296***	−0.201*	−0.223***	−0.099	−0.220*t*	−0.077	0.236**	−0.031	0.147*
	(0.061)	(0.078)	(0.059)	(0.095)	(0.116)	(0.080)	(0.085)	(0.100)	(0.074)
Race (non‐white = 1)	0.153**	−0.056	0.180**	−0.090	−0.556***	−0.438***	−0.241**	−0.581***	−0.630***
	(0.059)	(0.075)	(0.056)	(0.097)	(0.119)	(0.082)	(0.086)	(0.101)	(0.075)
Immigrant (born outside US = 1)	0.143*	0.078	0.025	0.285**	0.255*	0.299***	0.144*t*	0.360***	0.266***
	(0.057)	(0.073)	(0.054)	(0.091)	(0.111)	(0.076)	(0.081)	(0.095)	(0.070)
Depressive symptoms	−0.378***	−0.394***	−0.333***	0.036	0.099**	0.031	0.432***	0.514***	0.333***
	(0.020)	(0.026)	(0.019)	(0.031)	(0.037)	(0.026)	(0.027)	(0.032)	(0.024)
Education level									
Some college	−0.047	0.035	−0.036	0.259*	0.439**	0.194*	0.355***	0.499***	0.277**
	(0.070)	(0.090)	(0.067)	(0.115)	(0.140)	(0.097)	(0.102)	(0.120)	(0.089)
Associate degree	−0.100	−0.082	−0.126*t*	0.241*	0.401**	0.140	0.313**	0.525***	0.258**
	(0.074)	(0.096)	(0.071)	(0.120)	(0.147)	(0.101)	(0.107)	(0.126)	(0.093)
College	−0.354***	−0.284**	−0.180**	0.387***	0.662***	0.427***	0.729***	0.936***	0.579***
	(0.068)	(0.087)	(0.065)	(0.108)	(0.132)	(0.091)	(0.096)	(0.113)	(0.084)
Masters or more	−0.425***	−0.278**	−0.143*	0.442***	0.932***	0.356***	0.835***	1.164***	0.498***
	(0.071)	(0.091)	(0.068)	(0.112)	(0.137)	(0.095)	(0.100)	(0.117)	(0.087)
Social environments									
Parental warmth	−0.131***	−0.118***	−0.021	−0.032	−0.108**	−0.043*t*	0.097***	0.012	−0.066**
	(0.019)	(0.024)	(0.018)	(0.032)	(0.038)	(0.026)	(0.028)	(0.032)	(0.024)
Prosocial school environment	−0.058**	−0.176***	−0.122***	−0.117***	−0.180***	−0.038	−0.015	0.009	0.102***
	(0.019)	(0.023)	(0.018)	(0.031)	(0.037)	(0.026)	(0.027)	(0.032)	(0.023)
Family conflict	0.149***	0.205***	0.220***	0.124***	0.208***	0.255***	−0.085**	−0.024	0.006
	(0.018)	(0.023)	(0.017)	(0.030)	(0.036)	(0.025)	(0.027)	(0.031)	(0.023)
Neighborhood deprivation	0.047*t*	0.031	0.015	−0.006	0.040	−0.022	−0.018	0.040	−0.009
	(0.027)	(0.031)	(0.024)	(0.039)	(0.046)	(0.032)	(0.033)	(0.038)	(0.029)
Ave. outcome	0.393	1.199	0.400	0.300	0.853	0.888	−0.023	−0.255	0.417
*N* research sites	21	21	21	21	21	21	21	21	21
*N* families	8817	8783	8818	6266	6155	6262	6422	6457	6450
*N* person‐wave dyads	28,064	27,135	27,878	11,006	10,682	10,992	11,467	11,518	11,518

*Note*: Each column represents an independent model. All models were fitted using multilevel mixed‐effects linear regression and the estimations were performed by maximum likelihood using Stata 19 and the command MIXED.

Abbreviations: Ave., average; EXT, externalizing; INA, inattention; INT, internalizing; *N*, number.

***p < 0.001, **p < 0.01, *p < 0.05, *t* < 0.10.

Caregiver characteristics related to discrepancies. Caregivers reporting higher depressive symptoms reported significantly higher youth mental health symptoms compared to both youth (*p*'s < 0.001) and teachers (*p*'s < 0.001). Caregiver racial background also predicted discrepancies: non‐white caregivers reported lower symptom levels relative to teachers across all domains (*p*'s < 0.001), while caregivers with higher education (college or master’s degree) tended to report fewer symptoms than youth but more symptoms than teachers (*p* < 0.001). Additionally, female caregivers reported fewer symptoms than youth (*p* < 0.05) but slightly more than teachers (*p* < 0.05).

Lastly, socio‐experiential environments predicted discrepancies as well. Greater caregiver warmth was associated with fewer youth‐reported INT and INA symptoms relative to caregivers (*p*'s < 0.001), while family conflict consistently predicted higher symptom reports from youth compared to caregivers (*p*'s < 0.001) and teachers (*p*'s < 0.001). A prosocial school environment significantly reduced discrepancies, with youth reporting fewer symptoms relative to caregivers and teachers (*p*'s < 0.001). Neighborhood deprivation showed no association with any discrepancies.

### Reporters' discrepancies over time in the transition to adolescence

**FIGURE 3:**
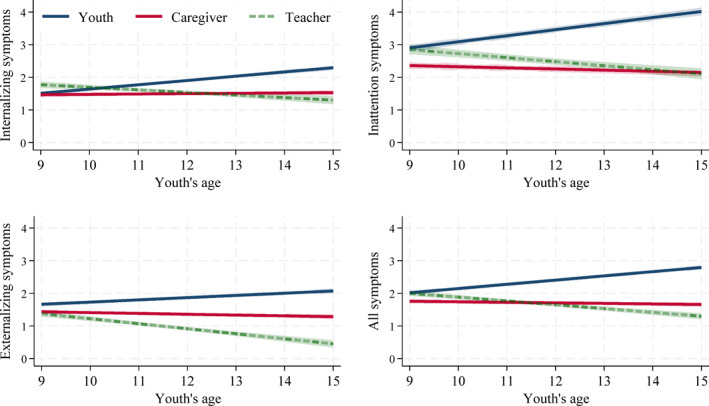
Mental health symptoms over youth age, by reporters and mental health domains.

Multilevel analyses also revealed distinct developmental trajectories for each reporter. Youth‐reported symptoms steadily increased across adolescence in all symptom domains, with the steepest rise observed for INA symptoms. In contrast, caregiver and teacher reports either remained stable or declined slightly with increasing youth age. Interaction terms indicated significant and progressive widening discrepancies between youth reports and both caregiver (*β* = −0.091 to −0.217, *p* < 0.001) and teacher reports (*β* = −0.209 to −0.307, *p* < 0.001) across all domains as youth transitioned through early adolescence (Figure 3; Supporting Information S1: Appendixes S4 and S6; Table S6).

To further examine these interactions, we performed simple slope analyses. As shown in Figure 3, between age 9 and 15 the youth‐reported symptoms increased steadily across age in all domains: INT (slope = 0.132), INA (slope = 0.186), EXT (slope = 0.073), and overall (slope = 0.133). In contrast, caregiver ratings remained relatively flat, with only small increases across adolescence: INT (slope = 0.012), INA (slope = −0.030), EXT (slope = −0.017), and overall symptoms (slope = −0.010). Teacher ratings showed a declining pattern: INT (slope = −0.078), INA (slope = −0.124), EXT (slope = −0.149), and overall (slope = −0.113). These findings illustrate that as youth move through early adolescence, their symptom self‐reports increase consistently, while caregivers show stability and teachers report fewer symptoms, especially in the EXT domain.

### Sensitivity analysis results

Sensitivity analyses examined the robustness of our findings in several ways (analyses are detailed in Supporting Information S1: Appendix S10). Results from these sensitivity analyses were highly consistent with the reported results.

## DISCUSSION

The present study shows that mean‐level reporter discrepancies are clinically meaningful, widespread, particularly for INA symptoms‐‐tend to increase as youth age and correlate with features of both reporters and environments. Discrepancies were more pronounced for some mental health symptoms (e.g., hyperactivity), highlighting that reporter discrepancies may be of particular concern for certain areas of clinical inquiry. Systematic patterns emerged based on features of the youth including, sex, racial background, caregiver education, and social context. Discrepancies increase over development which may reflect the normative social transition toward independence. These results underscore the importance of viewing informant discrepancies not only as noise or error, but as meaningful sources of information that may reflect developmental processes and context‐specific expression of youth mental health.

Youth consistently and increasingly reported higher levels of INA compared to caregivers and teachers, especially for symptoms involving restlessness, hyperactivity, and difficulty concentrating, consistent with previous studies (DuPaul et al., 2020). For INT and EXT symptoms, youth‐caregiver discrepancies were similar in magnitude. Discrepancies in INT symptoms widened over time, which aligns with previous reports (Booth et al., 2023; De Los Reyes et al., 2015; Kawabe et al., 2021). This result could reflect a youth's increasing awareness and introspection during adolescence (Hope et al., 1999; Penney & Skilling, 2012) or social reorientation away from adults towards peers (Nelson et al., 2016; Yang et al., 2021) among other developments. Conversely, youth‐teacher discrepancies widened more for EXT than for INT symptoms, driven by decreases in teacher ratings. This change in discrepancy may indicate that EXT behaviors become harder to observe by teachers as youth transition into adolescence; potentially making their reports less reliable for research focused on late adolescence (De Los Reyes et al., 2011; Olson et al., 2018).

Regarding the predictors of discrepancies, we identified relevant factors related to youth, caregivers, and social‐environmental characteristics. Relative to non‐females, female youth had greater discrepancies between participant's own self‐reported symptom severity and reports of their caregivers and teachers. These findings may reflect multiple theoretical processes, including increased symptom prevalence and greater awareness of internal states among girls, early socialization experiences encouraging females to mask negative emotions or distress to protect others' feelings (Chaplin & Aldao, 2013; Shi et al., 2021). For discrepancies related to EXT dimension, girls may be more comfortable acknowledging problems and less likely to be externally disruptive (Carlén et al., 2022); on the other hand, girls may be framed as less prone to hyperactivity and overt misbehavior, leading adults to overlook or misinterpret these behaviors more (Slobodin & Davidovitch, 2019). Consistent with our results, the combination of both phenomena may lead to an adult underestimation of girls' mental health symptoms. Empirical researchers should take this pattern into consideration when interpreting results based on adult reports.

Our findings also indicated significant discrepancies related to youth's racial background, with teachers reporting higher EXT symptoms for non‐white youth compared to their caregivers. Potential explanations include differing beliefs among these caregivers about what constitutes a mental health concern (Maddox et al., 2025), racist biases or differential expectations among teachers, even in the absence of objectively observed behavioral differences (Sabol et al., 2022), and “socialization discontinuity,” in which typical or normative behaviors among youth from minoritized backgrounds conflict with majority‐culture school norms and attract heightened teacher attention (Ginsberg, 2023). These findings add to a growing body of literature suggesting that racial background may influence early ratings of emerging symptoms in complex ways that reflect environmental features that contribute to them (De Los Reyes et al., 2013, 2023). Researchers should be cautious when using teacher reports only to study mental health outcomes for racial minority youth.

Caregiver characteristics also contributed to reporter discrepancies. Higher depressive symptoms in caregivers are related to elevated severity ratings of the youth's mental health symptoms across all domains compared to youth self‐reports and teacher ratings. This pattern may reflect depression‐distortion or true elevations in youths' symptoms associated with caregiver depression through shared familial and environmental pathways (Ehrlich et al., 2011; Gartstein et al., 2009; Olino et al., 2021; Richters, 1992). Additionally, caregivers with higher education tended to rate their children's symptoms lower than youth reports, yet higher than teacher assessments. This finding opposes previous studies reporting higher discrepancies for youth from low socioeconomic status (e.g., Stone et al., 2013; Van Roy et al., 2010). Lastly, discrepancies related to caregiver racial background and immigration status revealed further complexities. These differences could reflect biases or culturally specific expectations held by teachers regarding behavioral norms (Sabol et al., 2022). Conversely, teachers endorsed fewer symptoms than youth and caregivers when caregivers were immigrants, potentially indicating differing cultural perceptions of problematic behavior (Kim et al., 2013, 2016).

Social‐environmental characteristics were also significant predictors of reporter discrepancies, with both positive and negative contexts shaping how observers perceived youth mental health symptoms. Specifically, caregiver‐reported symptoms were systematically higher than youth self‐reports in contexts characterized by greater caregiver warmth, potentially indicating that caregivers who maintain close and open communication with their children may be paying more attention to their behavior and symptoms, which can indicate overprotective parenting or seeing problems that youth may not be aware of Ehrlich et al. (2011, 2014). Likewise, more prosocial school environments, were related to lower caregivers' and teachers' ratings relative to youth self‐reports. This finding aligns with research showing that even in generally positive school settings, discrepancies remain (Curhan et al., 2020). Conversely, higher family conflict was associated with increased youth self‐reporting of symptoms compared to caregivers and teachers and may reflect a stressful home environments (De Los Reyes et al., 2016; De Los Reyes & Kazdin, 2006; Lohaus et al., 2020). Notably, our analysis indicated no consistent relationship between neighborhood deprivation and informant discrepancies. Collectively, these findings point to the importance of measuring the quality of social contexts when studying multi‐informant reports of youth mental health.

Despite the contributions of this study, several limitations should be acknowledged. First, our sample was drawn from the general U.S. population, and although large and diverse, it was not clinically enriched. Therefore, caution is necessary when generalizing findings to other countries, clinical populations, or youths experiencing severe mental health symptoms. Second, the absence of an external benchmark or “gold standard” measure, such as clinician assessments, clinical diagnoses, or independent observational measures, limits our ability to determine which informant reports most accurately reflect true underlying symptoms; interpretations of such should be made with caution. Third, our assessments relied exclusively on subjective reports from youth, caregivers, and teachers, without incorporating more objective biomarkers or physiological indicators of mental health, which could provide complementary information and enhance validity. Additionally, teachers vary each year, with no demographic data provided, which may limit the conclusions that can be drawn from teacher reports. Future research addressing these limitations can further clarify how discrepancies across reporters reflect meaningful variations in mental health symptoms, thereby improving early identification and targeted intervention strategies.

## CONCLUSION

Discrepancies in youth mental health symptoms across INT, INA, and EXT domains during the critical developmental transition from childhood to adolescence. Discrepancies systematically varied by symptom type, reporter characteristics, and socio‐environmental contexts, underscoring the need to interpret these differences not merely as measurement error, but as meaningful information reflecting youths' developmental experiences, biases among observers, and contextual variations. Future studies should consider applying advanced analytic approaches to enhance early detection and improve accuracy in youth mental health assessments.

## AUTHOR CONTRIBUTIONS

**Matias Martinez**: Conceptualization; data curation; formal analysis; writing—original draft; visualization; writing—review and editing. **Vijay A. Mittal**: Writing—review and editing. **Jonathan D. Schaefer**: Writing—review and editing; supervision. **Claudia M. Haase**: Writing—review and editing; funding acquisition; resources. **Katherine S. F. Damme**: Writing—original draft; writing—review and editing; visualization; formal analysis; project administration; data curation; supervision; conceptualization.

## CONFLICT OF INTEREST STATEMENT

The authors declare no conflicts of interest.

## ETHICAL CONSIDERATIONS

Children verbally consented to the study, their caregivers provided written informed consent, and each participating research site’s institutional review board certified that the study complied with the biomedical ethics requirements for research with human subjects (Clark et al., 2018). The central Institutional Review Board (cIRB) at the University of California, San Diego, is responsible for the ethical review and approval of the research protocol and for coordinating all interactions between relying sites and the cIRB for data collection. The data analyses that were produced here were determined not to qualify as human research by the Institutional Review Boards of Northwestern University, Vanderbilt University, and the University of Texas at Dallas.

## Supporting information

Supporting Information S1

## Data Availability

Data used in the preparation of this article were obtained from the Adolescent Brain Cognitive Development™ (ABCD) Study, held in the NIH Brain Development Cohorts Data Sharing Platform. This is a multisite, longitudinal study designed to recruit more than 10,000 children aged 9–10 and follow them over 10 years into early adulthood.
