# Supplementary material for: Diverging perspectives on emerging mental health symptoms: Multi‐informant discrepancies and their associated determinants across the transition to adolescence in the Adolescent Brain Cognitive Development Study
Source: JCPP Adv. 2026 May 24:e70122. Online ahead of print. doi: 10.1002/jcv2.70122 (PMC13339503; doi:10.1002/jcv2.70122)
Supplement: Supplementary file 1 — Supporting Information S1 [file JCV2-9999-e70122-s001.docx]

**Reporter discrepancies of youth mental health symptoms:**

**Disagreements between youth, caregivers, and teachers**

**in the transition to adolescence**

**Supporting Information**

**Contents**

[**Appendix S1**: Additional details for main variables and descriptive statistics at baseline 2](#_Toc218956488)

[**Appendix S2**: Discrepancies are clinically meaningful 5](#_Toc218956489)

[**Appendix S3**: Replicating analyses at the symptom level 10](#_Toc218956490)

[**Appendix S4**: Estimates from model testing discrepancies over time 13](#_Toc218956491)

[**Appendix S5**: Alternative definitions for reporters’ discrepancies 15](#_Toc218956492)

[**(a) Exact mismatches** 15](#_Toc218956493)

[**(b) Presence versus absence mismatches** 22](#_Toc218956494)

[**(c) Predictors of absolute discrepancies, by domains and reporters** 29](#_Toc218956495)

[**Appendix S6**: Multiple imputation 33](#_Toc218956496)

[**(a) Missingness patterns** 33](#_Toc218956497)

[**(b) Implementing Multiple Imputation** 37](#_Toc218956498)

[**Appendix S7**: Correction of multiple testing 44](#_Toc218956499)

[**Appendix S8**: Alternative specifications for the predictors model 48](#_Toc218956500)

[**Appendix S9**: Alternative specifications for the model testing discrepancies over time 63](#_Toc218956501)

**Appendix S10:** Sensitivity analysis results……………………………………………………………………………………………67

# **Appendix S1**: Additional details for main variables and descriptive statistics at baseline

**Sample characteristics**

The demographic characteristics of the 11,832 youths (5,655 females, 6,177 males) in the sample are summarized in **Table 1** in the main manuscript (measures from the 1- through 3-year follow-ups) and **Table S1** in this section (baseline measures). Youth participants had an average age of 9.91 years (SD=0.62, range=8.92–11.08) at baseline and 11.93 years (SD=1.04, range=9.67–14.75) across the follow-up periods. Approximately half of the youth were female (48%) and nearly half identified as non-white (46%). A small proportion (3%) of youth participants were born outside the United States. Across follow-ups, youth self-reports averaged 1.86 (SD = 2.24) for internalizing and 3.36 (SD = 2.71) for inattention. For externalizing, youth means differ slightly depending on the comparison set because BPM-Y includes one additional externalizing item (youth–caregiver comparable: M = 1.97, SD = 1.90; youth–teacher comparable: M = 1.84, SD = 1.79). Caregiver-reported averages were slightly lower for internalizing (M=1.46, SD=1.90), inattention (M=2.20, SD=2.61), and externalizing symptoms (M=1.58, SD=1.98). Teachers reported the lowest average externalizing symptoms (M=0.94, SD=2.00), and slightly higher than caregivers for internalizing symptoms (M=1.54, SD=2.15) and inattention symptoms (M=2.41, SD=3.00).

Caregivers were predominantly female (89%), with an average age of 39.95 years (SD=6.84) at baseline. Approximately one-quarter of caregivers identified as non-white (24%) and 18% were immigrants. Educational attainment of caregivers varied, with 17% having completed high school or less, 16% some college, 13% an associate degree, 28% a bachelor's degree, and 25% holding a master’s degree or higher. Caregiver-reported depressive symptoms at baseline averaged 3.97 (SD=3.66) on a scale from 0 to 28.

Regarding socio-experiential environments, youths reported relatively high parental warmth (M=2.78, SD=0.30 on a 1-3 scale) and a positive prosocial school environment (M=3.32, SD=0.47 on a 1-4 scale). Family conflict was relatively low, with an average score of 0.23 (SD=0.22 on a 0-1 scale). Neighborhood deprivation scores averaged 0.95 (SD=0.21, range=0.01–1.26), indicating generally moderate levels of neighborhood disadvantage across the sample.

*Youth mental health*

**Self-reports** were measured using the Brief Problems Monitor (BPM-Y). The BPM-Y is an abbreviated version of the Child Behavior Checklist (CBCL) including 19 items measuring three subscales: six internalizing symptoms (e.g., feels worthless; too fearful or anxious), six symptoms of inattention (e.g., can't concentrate for long; restless or hyperactive), and seven externalizing symptoms (e.g., argues a lot; threatens people). The extra externalizing item on the youth version originates in questions about disobedient behavior at home and school (Karcher & Barch, 2020; Pedersen et al., 2021; Piper et al., 2014). The instrument targets youth ages 6 to 18 years and is collected every six months starting on the six-month follow-up. The response options range from 0 to 2 (0=not true, 1= somewhat true, or 2=very true). We used data from the one- (Cronbach alpha for internalizing- (*α_1I_*)=0.745, inattention- (*α_1A_*)=0.766, and externalizing-symptoms (*α_1E_*)=0.665), two (*α_2I_* =0.780; *α_2A_*=0.777; *α_2E_*=0.661), and three-year follow-up (*α_3I_* =0.802; *α_3A_*=0.788; *α_3E_*=0.660).

**Teacher reports** of youth mental health symptoms were also measured using the Brief Problems Monitor (BPM-T). The BPM-T includes 18 out of the 19 items in BPM-Y and measures the same three subscales (i.e., internalizing, inattention, and externalizing symptoms). The excluded question asks about youth disobedient behavior at home. This instrument is also collected every six months, and we examined data collected at the one- (*α_1I_*=0.827; *α_1A_*=0.886; *α_1E_*=0.872), two (*α_2I_*=0.833; *α_2A_*=0.888; *α_2E_*=0.856), and three-year follow-up (*α_3I_*=0.838; *α_3A_*=0.865; *α_3E_*=0.864).

**Caregiver reports** of youth mental health symptoms were measured using the CBCL, a 119-item questionnaire completed by caregivers that assess their child’s behavioral, social, and emotional problems (Achenbach & Rescorla, 2001; Michelini et al., 2019). The three subscales measured in the BPM-Y and BPM-T (i.e., internalizing, inattention, and externalizing symptoms) were measured using the corresponding 18 items present across instruments. When comparing youth-caregiver reports, the self-report of disobedience at school was excluded, and when comparing caregiver-teacher reports, we compared reports of disobedience at home for caregivers and at school for teachers. The CBCL is collected every year starting at baseline. Our analyses used reports collected at the one- (*α_1I_* =.756; *α_1A_* =.852; *α_1E_* =.785), two (*α_2I_* =.762; *α_2A_* =.847; *α_2E_* =.783), and three-year follow-up (*α_3I_* =.776; *α_3A_* =.846; *α_3E_* =.773).

**Table S1.** Summary statistics measured at baseline

|  | Range | N | Mean | SD |
| --- | --- | --- | --- | --- |
|  | (1) | (2) | (3) | (4) |
|  |  |  |  |  |
| **Youth mental health symptoms** |  |  |  |  |
| Internalizing symptoms, caregiver reports | 0-12 | 11,825 | 1.44 | 1.83 |
| Inattention symptoms, caregiver reports | 0-12 | 11,825 | 2.37 | 2.71 |
| Externalizing symptoms, caregiver reports | 0-12 | 11,824 | 1.73 | 2.07 |
| **Youth characteristics** |  |  |  |  |
| Age (in years) | 8.92-11.08 | 11,831 | 9.91 | 0.62 |
| Sex at birth (girl = 1) | 0-1 | 11,832 | 47.79% | 0.50 |
| Race (non-white = 1) | 0-1 | 11,830 | 47.96% | 0.50 |
| Pubertal development, at baseline | 1-4 | 11,760 | 1.67 | 0.52 |
| Immigrant (born outside US = 1) | 0-1 | 11,816 | 2.95% | 0.17 |
| **Caregiver characteristics** |  |  |  |  |
| Age (in years) | 23-80 | 11,742 | 39.95 | 6.84 |
| Sex at birth (female = 1) | 0-1 | 11,825 | 89.03% | 0.31 |
| Race (non-white = 1) | 0-1 | 11,809 | 25.7% | 0.44 |
| Immigrant (born outside US = 1) | 0-1 | 11,827 | 17.6% | 0.38 |
| Depressive symptoms, at baseline | 0-28 | 11,827 | 3.97 | 3.66 |
| Primary caregiver's education, at baseline |  |  |  |  |
| High school or less | 0-1 | 11,815 | 17.27% | 0.38 |
| Some College | 0-1 | 11,815 | 16.45% | 0.37 |
| Associate degree | 0-1 | 11,815 | 12.98% | 0.34 |
| College | 0-1 | 11,815 | 28.09% | 0.45 |
| Masters or more | 0-1 | 11,815 | 25.21% | 0.43 |
| **Social Environments, measured at baseline** |  |  |  |  |
| Parental warmth | 1-3 | 11,798 | 2.78 | 0.30 |
| Prosocial school environment | 1-4 | 11,808 | 3.32 | 0.47 |
| Family conflict | 0-1 | 11,808 | 0.23 | 0.22 |
| Neighborhood deprivation | 0.01-1.26 | 10,955 | 0.95 | 0.21 |
|  |  |  |  |  |
|  |  |  |  |  |
| N: Number of observations (one observation per youth); SD: Standard deviation | | | | |
| **Notes**: Range corresponds to observed range in the data | | | | |

# **Appendix S2**: Discrepancies are clinically meaningful

To further study whether the discrepancies are not only statistically significant but also clinically meaningful, we conducted three additional exercises based the 0–12 domain scores for internalizing, inattention, and externalizing symptoms (each based on six items, using the common 6-item set across reporters). First, we treated these scores as if they were used to define a threshold-based diagnosis for each domain based on sum-scores and examined how often youth would be classified as meeting a diagnosis criterion across a wide range of plausible cut points (3–12). As summarized in **Table S2**, even for relatively stringent thresholds, reporter discrepancies have meaningful implications. For example, if we use a threshold of 8 for each of the three mental health domains under study (e.g., all six symptoms present and at least two of them rated as more frequent or intense), the prevalence of “internalizing disorder” would be 3.09% based on youth reports, 1.51% based on caregiver reports, and 2.47% based on teacher reports. That is, youth-based prevalence is roughly double the caregiver-based prevalence and notably higher than teacher-based prevalence. For inattention, at the same threshold of 8, teachers would identify 9.05% of youth as meeting criteria, compared with 8.85% based on youth self-reports and 5.53% based on caregiver reports (i.e., teacher-based prevalence is more than 60% higher than caregiver-based prevalence). While the prevalence decreases with the threshold for meeting diagnosis criteria, the diagnosis discrepancies emerge across the range of cut points we examined.

Second, we examined diagnosis mismatches between pairs of reporters. For each domain and threshold, we coded a mismatch whenever one reporter classified a youth as above threshold and the other as below threshold and then calculated the proportion of dyads with such a disagreement. As shown in **Table S3**, these mismatches are particularly frequent for low thresholds, although they are still meaningful when using stricter thresholds. For instance, at an inattention threshold of 8, 11.09% of youth-caregiver dyads, 13.15% of youth–teacher dyads, and 10.44% of caregiver–teacher dyads disagree on whether the youth meet the threshold; for internalizing at the same threshold, youth–caregiver and youth–teacher mismatches are 3.87% and 4.97%, respectively.

Third, we also linked symptom discrepancies on the Brief Problem Monitor (BPM) to diagnosis discrepancies on the K-SADS at the 2-year follow-up (KSADS diagnoses are not available at the 1-year follow-up or 3-year follow-up). Due to data constraints (i.e., no teacher reports in KSADS and not all disorders reported by youth and caregivers), we focused on diagnoses for which both youth and caregiver reports are available (i.e., depressive disorders, anxiety disorders, a broad internalizing cluster, and externalizing disorders). For each domain, we created a binary indicator of diagnosis mismatch (youth vs caregiver K-SADS diagnosis differs) and regressed this outcome on the absolute youth-caregiver discrepancy in the corresponding BPM score (for depression and anxiety we used the three corresponding items within the internalizing domain). Results show that larger symptom discrepancies are systematically associated with a higher probability of K-SADS diagnoses mismatches (see **Table S4**). For example, for internalizing difficulties, a one-unit increase in youth-caregiver BPM discrepancy is associated with an approximately 3-4 percentage-point increase in the likelihood of a youth-caregiver K-SADS diagnosis mismatch in linear probability models (b ≈ 0.036) and about a 3 percentage-point marginal effect in logit models. Given that the average internalizing discrepancy is around 0.4 symptoms, this corresponds to roughly a 1-2 percentage-point higher chance that youth and caregiver K-SADS diagnoses diverge when symptom ratings differ to the extent we observe on average. Similar patterns were observed for depression, anxiety, and externalizing domains, with somewhat smaller but still statistically robust effects for externalizing symptoms.

These findings support the idea that informant discrepancies in symptom reports observed in the study are clinically meaningful and underscore the need for researchers and clinicians to be explicit about how reporter choice may affect estimates of disorder prevalence and identification.

**Table S2.** Diagnosis rates for different number/intensity of symptom thresholds

|  | Internalizing | | |  | Inattention | | |  | Externalizing | | |
| --- | --- | --- | --- | --- | --- | --- | --- | --- | --- | --- | --- |
|  | Youth | Caregiver | Teacher |  | Youth | Caregiver | Teacher |  | Youth | Caregiver | Teacher |
| Symptom threshold | | | | | | | | | | | |
| 3 | 28.31% | 21.74% | 23.16% |  | 56.03% | 35.41% | 37.10% |  | 33.16% | 25.28% | 19.69% |
| 4 | 18.91% | 13.03% | 15.52% |  | 43.41% | 25.96% | 28.89% |  | 19.54% | 15.41% | 11.00% |
| 5 | 12.28% | 7.85% | 10.41% |  | 31.95% | 18.01% | 21.91% |  | 10.41% | 8.65% | 6.54% |
| 6 | 8.12% | 4.69% | 6.75% |  | 22.14% | 12.12% | 16.77% |  | 5.58% | 5.27% | 3.76% |
| 7 | 5.05% | 2.72% | 4.13% |  | 14.26% | 8.05% | 12.38% |  | 2.60% | 3.11% | 2.16% |
| 8 | 3.09% | 1.51% | 2.47% |  | 8.85% | 5.53% | 9.05% |  | 1.22% | 1.85% | 1.24% |
| 9 | 1.94% | 0.82% | 1.66% |  | 4.82% | 3.44% | 6.09% |  | 0.48% | 0.97% | 0.59% |
| 10 | 1.08% | 0.43% | 0.85% |  | 2.12% | 2.00% | 3.63% |  | 0.19% | 0.43% | 0.24% |
| 11 | 0.53% | 0.23% | 0.39% |  | 0.69% | 0.95% | 1.81% |  | 0.04% | 0.17% | 0.10% |
| 12 | 0.22% | 0.08% | 0.19% |  | 0.16% | 0.25% | 0.50% |  | 0.01% | 0.04% | 0.02% |
|  |  |  |  |  |  |  |  |  |  |  |  |
|  |  |  |  |  |  |  |  |  |  |  |  |
|  |  |  |  |  |  |  |  |  |  |  |  |

**Table S3.** Diagnosis mismatches by number/intensity of symptom thresholds and reporter

|  | Internalizing | | |  | Inattention | | |  | Externalizing | | |
| --- | --- | --- | --- | --- | --- | --- | --- | --- | --- | --- | --- |
|  | Y - C | Y - T | C - T |  | Y - C | Y - T | C - T |  | Y - C | Y - T | C – T |
| Symptom threshold | | | | | | | | | | | |
| 3 | 30.14% | 32.15% | 29.05% |  | 39.97% | 40.21% | 30.32% |  | 32.95% | 31.75% | 6.15% |
| 4 | 21.54% | 24.09% | 20.60% |  | 36.10% | 35.82% | 25.61% |  | 22.98% | 21.03% | 4.71% |
| 5 | 14.77% | 16.78% | 14.51% |  | 29.55% | 29.58% | 20.99% |  | 13.89% | 12.66% | 2.36% |
| 6 | 9.97% | 11.34% | 9.62% |  | 22.57% | 23.67% | 17.27% |  | 8.46% | 7.34% | 1.64% |
| 7 | 6.35% | 7.67% | 6.13% |  | 16.16% | 17.83% | 13.66% |  | 4.75% | 4.00% | 1.05% |
| 8 | 3.87% | 4.97% | 3.55% |  | 11.09% | 13.15% | 10.44% |  | 2.68% | 2.15% | 0.68% |
| 9 | 2.34% | 3.32% | 2.29% |  | 6.74% | 8.77% | 7.76% |  | 1.31% | 0.96% | 0.42% |
| 10 | 1.30% | 1.73% | 1.20% |  | 3.58% | 4.79% | 4.93% |  | 0.56% | 0.37% | 0.22% |
| 11 | 0.65% | 0.88% | 0.56% |  | 1.50% | 2.24% | 2.53% |  | 0.18% | 0.11% | 0.07% |
| 12 | 0.28% | 0.35% | 0.27% |  | 0.39% | 0.61% | 0.68% |  | 0.04% | 0.02% | 0.02% |
|  |  |  |  |  |  |  |  |  |  |  |  |
|  |  |  |  |  |  |  |  |  |  |  |  |
| Y: Youth; C: Caregiver; T: Teacher | | |  |  |  |  |  |  |  |  |  |

**Table S4.** Symptom discrepancies as predictors of diagnosis mismatches, youth versus caregiver reports

|  | Linear models | | | |  | Logit models | | | |  |
| --- | --- | --- | --- | --- | --- | --- | --- | --- | --- | --- |
|  | DM-DEP | DM-ANX | DM-INT | DM-EXT |  | DM-DEP | DM-ANX | DM-INT | DM-EXT |  |
|  | (1) | (2) | (3) | (4) |  | (5) | (6) | (7) | (8) |  |
| Discrepancies in symptoms (absolute values) | | | | | | | | | |  |
| Depressive symptoms | 0.055*** |  |  |  |  | 0.031*** |  |  |  |  |
|  | (0.004) |  |  |  |  | (0.002) |  |  |  |  |
| Anxiety symptoms |  | 0.027*** |  |  |  |  | 0.023*** |  |  |  |
|  |  | (0.003) |  |  |  |  | (0.002) |  |  |  |
| Internalizing symptoms |  |  | 0.036*** |  |  |  |  | 0.027*** |  |  |
|  |  |  | (0.002) |  |  |  |  | (0.001) |  |  |
| Externalizing symptoms |  |  |  | 0.013*** |  |  |  |  | 0.009*** |  |
|  |  |  |  | (0.002) |  |  |  |  | (0.001) |  |
|  |  |  |  |  |  |  |  |  |  |  |
|  |  |  |  |  |  |  |  |  |  |  |
| Ave. outcome | 0.0476 | 0.0790 | 0.109 | 0.0249 |  | 0.0476 | 0.079 | 0.109 | 0.0249 |  |
| N research sites | 21 | 21 | 21 | 21 |  | 21 | 21 | 21 | 21 |  |
| N families | 8,525 | 8,525 | 8,525 | 8,525 |  | 8,525 | 8,525 | 8,525 | 8,525 |  |
| N youth dyads | 10,223 | 10,223 | 10,223 | 10,223 |  | 10,223 | 10,223 | 10,223 | 10,223 |  |
|  |  |  |  |  |  |  |  |  |  |  |
|  |  |  |  |  |  |  |  |  |  |  |
| DM: Diagnosis mismatch; DEP: Depression; ANX: Anxiety; INT: Internalizing; EXT: Externalizing; N: Number. | | | | | | | | | |  |
| **Notes**: Each column represents an independent model. Linear models were fitted using multilevel mixed-effects linear regression and the estimations were performed by maximum likelihood using Stata 19 and the command MIXED. Logit models were fitted using Stata 19 and the command LOGIT, clustering the standard error at the family level. *** p<0.001, ** p<0.01, * p<0.05, t <0.10. | | | | | | | | | |  |
|  |  |  |  |  |  |  |  |  |  |  |

# **Appendix S3**: Replicating analyses at the symptom level

Following prior work showing significant symptom heterogeneity within single disorders (e.g., previous research reporting that there are 227 different ways to combine symptoms to be diagnosed with depression, which can result in two individuals with the same diagnosis sharing only one out of nine symptoms-related criteria; see Hyman, 2010; Newson et al., 2021), we explored discrepancies at the item level (see **Table S5**). Discrepancies varied substantially across items, with some of the largest differences clustering around behaviors across mental health domains related to hyperactivity and impulsivity as well as irritability and argumentativeness. For example, youth reported markedly higher inattention-related symptoms than caregivers, particularly for restlessness/hyperactivity (Youth [Y] – Caregiver [C] = 0.519), difficulty concentrating (0.248), inattentiveness/distractibility (0.224), and impulsivity (0.215). By contrast, several internalizing items showed smaller differences (e.g., unhappy/sad Y–C = 0.019; feels worthless Y–C = −0.011). In the externalizing domain, the direction of discrepancies was symptom-specific: youth reported fewer disobedience to parents symptoms than caregivers (Y–C = −0.149), while reporting higher irritability (0.208) and hot temper (0.209).

We next examined how symptom-level discrepancies changed across the transition to early adolescence using the difference between the mean discrepancy at the 3-year and 1-year follow-ups (see **Figure S1**). Overall, discrepancies tended to increase over time, with the clearest growth in youth–teacher differences. In youth–caregiver comparisons, the largest increases were observed for restlessness/hyperactivity, difficulty concentrating, stubborn/irritable, and easily embarrassed, whereas several items showed little change or small declines (e.g., destroys property; acts too young; threatens people). In youth–teacher comparisons, the largest increases occurred for hot temper, the anxiety domain, and impulsivity, while some low-frequency behaviors changed minimally (e.g., destroys property; threatens people). Finally, caregiver–teacher discrepancies were near zero for internalizing overall but showed growing divergence for select behaviors over time, most notably impulsivity and arguing a lot, with generally small changes for other items. Together, these symptom-level results highlight *hot spots* where reporter differences are largest and where disagreement grows most strongly as youth transition into early adolescence.

**Table S5.** Mean-level differences, by reporters and mental health symptoms

|  | Differences in symptoms, by reporters | | |  |
| --- | --- | --- | --- | --- |
|  | Youth –  Caregiver | Youth –  Teacher | Caregiver - Teacher |  |
|  | (1) | (2) | (3) |  |
|  |  |  |  |  |
| Internalizing symptoms | 0.405 [0.165] *** | 0.304 [0.111] *** | -0.027 [-0.011] |  |
| Anxiety symptoms | 0.301 [0.173] *** | 0.288 [0.154] *** | 0.062 [0.036]t |  |
| Too fearful or anxious | 0.088 [0.137] *** | 0.09 [0.131] *** | 0.014 [0.023] |  |
| Easily embarrassed | 0.053 [0.064] *** | 0.101 [0.119] *** | 0.073 [0.092] *** |  |
| Worries | 0.160 [0.202] *** | 0.097 [0.117] *** | -0.025 [-0.033] *** |  |
| Depressive symptoms | 0.104 [0.096] *** | 0.016 [0.013] | -0.089 [-0.08] *** |  |
| Feels worthless | -0.011 [-0.023] *** | -0.043 [-0.081] *** | -0.03 [-0.058] *** |  |
| Feels too guilty | 0.096 [0.200] *** | 0.076 [0.147] *** | -0.016 [-0.041] *** |  |
| Unhappy, sad, or depressed | 0.019 [0.037] *** | -0.017 [-0.03] ** | -0.043 [-0.079] *** |  |
|  |  |  |  |  |
| Inattention symptoms | 1.198 [0.398] *** | 0.842 [0.255] *** | -0.259 [-0.088] *** |  |
| Acts too young for age | 0.062 [0.096] *** | 0.062 [0.094] *** | -0.009 [-0.015] t |  |
| Fails to finish things they start | -0.07 [-0.092] *** | -0.054 [-0.07] *** | 0.021 [0.028] *** |  |
| Can't concentrate for long | 0.248 [0.315] *** | 0.154 [0.186] *** | -0.068 [-0.095] *** |  |
| Restless or hyperactive | 0.519 [0.625] *** | 0.479 [0.548] *** | -0.013 [-0.019] * |  |
| Impulsive | 0.215 [0.31] *** | 0.129 [0.171] *** | -0.076 [-0.115] *** |  |
| Inattentive or easily distracted | 0.224 [0.268] *** | 0.073 [0.083] *** | -0.114 [-0.147] *** |  |
|  |  |  |  |  |
| Externalizing symptoms | 0.406 [0.179] *** | 0.877 [0.385] *** | 0.404 [0.187] *** |  |
| Argues a lot | 0.14 [0.173] *** | 0.381 [0.485] *** | 0.242 [0.322] *** |  |
| Destroys property | -0.025 [-0.078] *** | -0.007 [-0.024] *** | 0.018 [0.054] *** |  |
| Disobedient to parents | -0.149 [-0.248] *** | -0.088 [-0.193] *** | -0.047 [-0.113] *** |  |
| Stubborn, sullen, or irritable | 0.208 [0.255] *** | 0.308 [0.372] *** | 0.118 [0.166] *** |  |
| Temper, tantrums, or hot temper | 0.209 [0.289] *** | 0.296 [0.441] *** | 0.11 [0.188] *** |  |
| Threatens people | 0.023 [0.081] *** | -0.013 [-0.035] *** | -0.038 [-0.119] *** |  |
|  |  |  |  |  |
|  |  |  |  |  |
| **Notes:** Results based on the BPM-Y, BPM-T, and CBCL, which are available for all participants from baseline to the three-year follow-up for the caregiver and teacher, and from the one-year follow-up to the three-year follow-up for the youth. We focused our analysis on data from one- to three-year follow-up. Columns (1) to (3) show the average difference between reporters and the statistical significance according to a paired t-tests. Standardized differences in brackets. Differences in Column (1) are based on around 30,000 observations, and differences in reported in Columns (2) and (3) are based on around 12,000 observations *** p<0.001, ** p<0.01, * p<0.05, t <0.10. | | | |  |
|  |  |  |  |  |
|  |  |  |  |  |
|  |  |  |  |  |
|  |  |  |  |  |
|  |  |  |  |  |

**Figure S1.** Discrepancies in item response over time

| 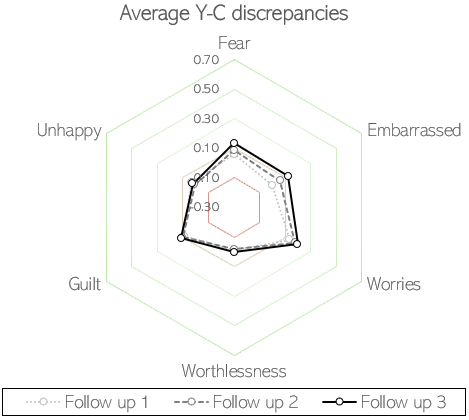 | 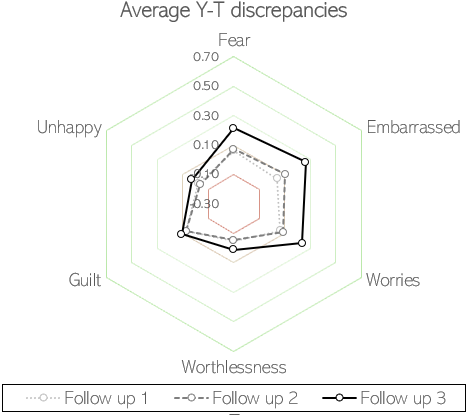 | 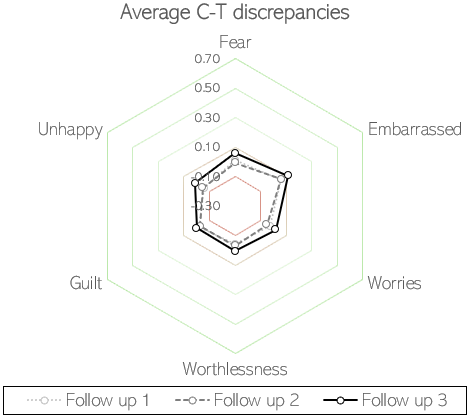 |
| --- | --- | --- |
| 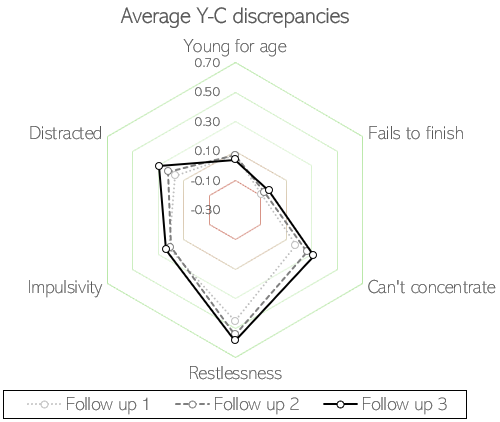 | 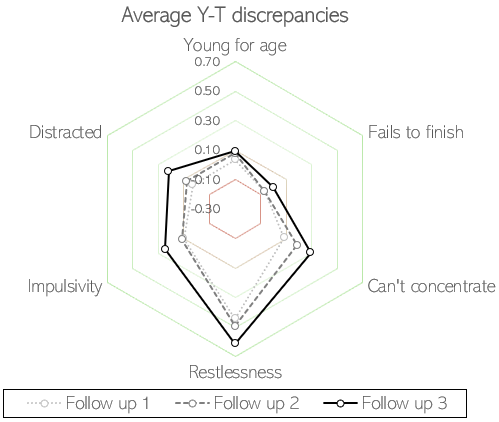 | 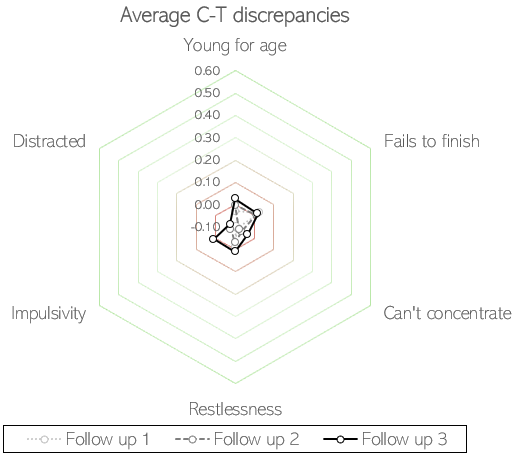 |
| 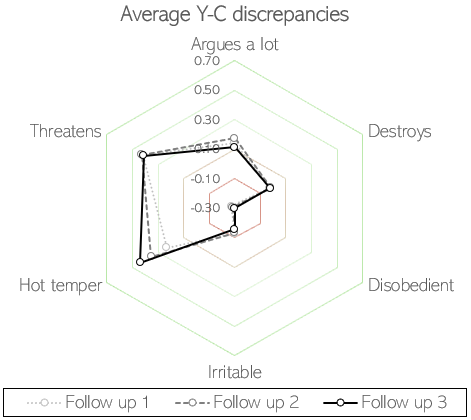 | 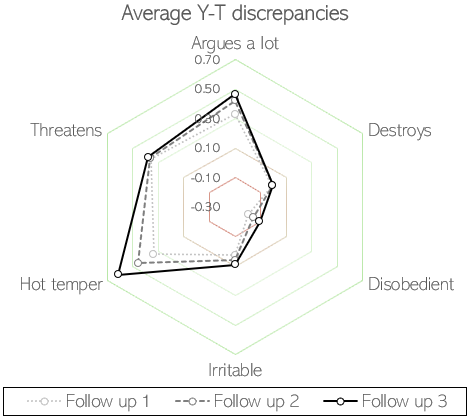 | 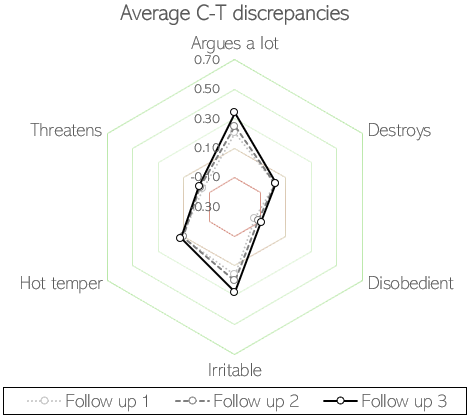 |
|  |  |  |

# **Appendix S4:** Estimates from model testing discrepancies over time

**Table S6.** Predictors of mean-level discrepancies and changes over time (domains x reporters-level data)

|  | Mental health domains | | | |  |
| --- | --- | --- | --- | --- | --- |
|  | INT | INA | EXT | All |  |
|  | (1) | (2) | (3) | (4) |  |
|  |  |  |  |  |  |
| Reporters | | | | |  |
| Caregivers | 1.039*** | 1.442*** | 0.605*** | 1.054*** |  |
|  | (0.168) | (0.205) | (0.142) | (0.112) |  |
| Teachers | 2.150*** | 2.731*** | 1.708*** | 2.201*** |  |
|  | (0.231) | (0.280) | (0.195) | (0.153) |  |
| Caregivers x youth's age | -0.120*** | -0.221*** | -0.093*** | -0.146*** |  |
|  | (0.014) | (0.017) | (0.012) | (0.009) |  |
| Teachers x youth's age | -0.210*** | -0.309*** | -0.222*** | -0.246*** |  |
|  | (0.020) | (0.024) | (0.017) | (0.013) |  |
| Youth's characteristics | | | | |  |
| Age (in years) | 0.131*** | 0.186*** | 0.068*** | 0.129*** |  |
|  | (0.011) | (0.013) | (0.009) | (0.007) |  |
| Sex at birth (girl = 1) | 0.367*** | -0.611*** | -0.169*** | -0.127*** |  |
|  | (0.024) | (0.032) | (0.021) | (0.019) |  |
| Race (non-white = 1) | 0.069t | 0.248*** | 0.099** | 0.130*** |  |
|  | (0.036) | (0.051) | (0.032) | (0.032) |  |
| Pubertal development | 0.086*** | 0.062*** | 0.047*** | 0.056*** |  |
|  | (0.011) | (0.015) | (0.010) | (0.009) |  |
| Immigrant (born outside US = 1) | 0.036 | 0.021 | 0.002 | 0.033 |  |
|  | (0.077) | (0.108) | (0.068) | (0.068) |  |
| Caregiver's characteristics | | | | |  |
| Age (in years) | 0.002 | 0.003 | -0.002 | 0.001 |  |
|  | (0.002) | (0.003) | (0.002) | (0.002) |  |
| Sex at birth (female = 1) | -0.041 | 0.067 | 0.113** | 0.050 |  |
|  | (0.041) | (0.056) | (0.036) | (0.035) |  |
| Race (non-white = 1) | -0.248*** | -0.133* | -0.025 | -0.127*** |  |
|  | (0.039) | (0.055) | (0.035) | (0.035) |  |
| Immigrant (born outside US = 1) | -0.026 | -0.193*** | -0.134*** | -0.121*** |  |
|  | (0.038) | (0.054) | (0.034) | (0.034) |  |
| Depressive symptoms | 0.359*** | 0.451*** | 0.275*** | 0.361*** |  |
|  | (0.013) | (0.019) | (0.012) | (0.012) |  |
|  |  |  |  |  |  |
|  |  |  |  |  |  |
|  |  |  |  |  |  |
|  |  |  |  |  |  |
| (Continues in the next page) | | | | |  |
|  |  |  |  |  |  |
|  |  |  |  |  |  |
| Education level |  |  |  |  |  |
| Some College | 0.042 | 0.063 | -0.029 | 0.016 |  |
|  | (0.047) | (0.066) | (0.042) | (0.042) |  |
| Associate Degree | 0.059 | 0.000 | -0.009 | 0.011 |  |
|  | (0.050) | (0.070) | (0.044) | (0.044) |  |
| College | 0.020 | -0.168** | -0.148*** | -0.098* |  |
|  | (0.046) | (0.064) | (0.040) | (0.041) |  |
| Masters or more | 0.043 | -0.243*** | -0.172*** | -0.131** |  |
|  | (0.048) | (0.067) | (0.042) | (0.042) |  |
| Social environments | | | | |  |
| Parental warmth | -0.037** | -0.121*** | -0.104*** | -0.087*** |  |
|  | (0.012) | (0.016) | (0.011) | (0.010) |  |
| Prosocial school environment | -0.111*** | -0.132*** | -0.084*** | -0.101*** |  |
|  | (0.012) | (0.016) | (0.010) | (0.010) |  |
| Family conflict | 0.105*** | 0.221*** | 0.164*** | 0.153*** |  |
|  | (0.012) | (0.016) | (0.010) | (0.009) |  |
| Neighborhood deprivation | 0.020 | 0.065* | 0.057*** | 0.050** |  |
|  | (0.016) | (0.026) | (0.016) | (0.016) |  |
|  |  |  |  |  |  |
|  |  |  |  |  |  |
| Average outcome | 1.640 | 2.700 | 1.476 | 1.936 |  |
| Number of observations | 69,146 | 68,264 | 69,053 | 206,463 |  |
|  |  |  |  |  |  |
|  |  |  |  |  |  |
| INT: Internalizing; INA: Inattention; EXT: Externalizing; Ave.: Average; N: Number | | | | |  |
| **Notes**: Each column represents an independent model. All models were fitted using multilevel mixed-effects linear regression and the estimations were performed by maximum likelihood using Stata 19 and the command MIXED. *** p<0.001, ** p<0.01, * p<0.05, t <0.10. | | | | |  |
|  |  |  |  |  |  |
|  |  |  |  |  |  |
|  |  |  |  |  |  |

# **Appendix S5**: Alternative operationalizations for reporters’ discrepancies

In this section, we tested the robustness of our main findings across research aims to different operationalizations for reporters’ discrepancies: (a) exact rating mismatches (informants giving different scores; averaged across items), (b) mismatches in symptom presence versus absence (one informant reporting a symptom present while the other did not; averaged across items), and (c) absolute difference scores (i.e., discrepancy $[D]=\left| Reporter1 - Reporter2 \right|$) only for research aim 2 on predictors of discrepancies).

## **(a) Exact mismatches**

We conducted analyses examining the magnitude of mismatches between informants, defined as the proportion of cases where informants provided different ratings for symptoms (exact mismatch). Across all three domains, exact mismatches were substantial. Youth-caregiver mismatches were observed for approximately 31% of internalizing, 46% of inattention, and 31% of externalizing symptoms. Youth-teacher mismatches showed slightly higher values, with approximately 32% for internalizing, 46% for inattention, and 28% for externalizing symptoms. Caregiver-teacher mismatches occurred in approximately 31% of internalizing, 36% of inattention, and 22% of externalizing symptoms (see **Table S7**).

We next replicated Research Aim 2 using exact mismatches as the outcome (see **Table S8**). Several predictors mirrored the patterns observed in the main discrepancy analyses. In particular, caregiver depressive symptoms and family conflict were consistently associated with higher mismatch rates across reporter pairs and domains, while parental warmth and a prosocial school environment were associated with lower mismatch rates. Mismatch rates also varied systematically by youth characteristics, with older age and more advanced pubertal development were generally associated with higher mismatch rates for youth–adult comparisons (especially for inattention), while patterns by sex and race differed by symptom domain and reporter pair. Higher caregiver education tended to predict lower mismatch rates, especially for youth–teacher and caregiver–teacher comparisons.

Finally, we examined whether exact mismatches varied across the transition to early adolescence using models with reporter indicators and reporter-by-age interactions (see **Table S9**, and **Figure S2**). Consistent with the descriptive patterns, exact mismatches between youth self-reports and caregiver/teacher reports increase with age for internalizing and inattention (and for the pooled all-domains measure), whereas caregiver–teacher mismatches are stable or decline. This pattern is consistent with early adolescence as a period of increasing autonomy and “social reorientation,” in which youths’ emotional experiences and attentional difficulties may become less observable to adults and increasingly context-dependent (e.g., occurring in peer settings, online, or internally), while youths’ self-awareness and willingness to report subjective experiences may increase. In contrast, caregivers and teachers—both external observers—may converge in their judgments over time because they rely on similar observable cues and normative expectations for age-appropriate behavior. The relatively flatter age trends for externalizing are also consistent with these symptoms being more overt and therefore easier for different informants to detect similarly.

**Table S7.** Exact mismatches, by reporters and mental health domains

|  | Exact mismatches in symptoms, by reporters | | |
| --- | --- | --- | --- |
|  | Youth - Caregiver | Youth - Teacher | Caregiver - Teacher |
|  | (1) | (2) | (3) |
|  |  |  |  |
| Internalizing symptoms | 30.033% | 32.481% | 30.509% |
| Inattention symptoms | 45.175% | 45.729% | 35.762% |
| Externalizing symptoms | 30.666% | 27.809% | 22.119% |
|  |  |  |  |
|  |  |  |  |
| **Notes:** Results based on the BPM-Y, BPM-T, and CBCL, which are available for all participants from baseline to the three-year follow-up for the caregiver and teacher, and from the one-year follow-up to the three-year follow-up for the youth. We focused our analysis on data from one- to three-year follow-up. Exact mismatches in Column (1) are based on around 30,000 observations, and differences in reported in Columns (2) and (3) are based on around 12,000 observations. | | | |

**Table S8.** Predictors of reporters’ exact mismatches

|  | Youth – Caregiver | | |  | Youth - Teacher | | |  | Caregiver - Teacher | | |  |
| --- | --- | --- | --- | --- | --- | --- | --- | --- | --- | --- | --- | --- |
|  | INT | INA | EXT |  | INT | INA | EXT |  | INT | INA | EXT |  |
|  | (1) | (2) | (3) |  | (4) | (5) | (6) |  | (7) | (8) | (9) |  |
|  |  |  |  |  |  |  |  |  |  |  |  |  |
| Youth's characteristics | | | | | | | | | | | |  |
| Age (in years) | 0.003* | 0.011*** | -0.002 |  | 0.009*** | 0.011*** | 0.001 |  | -0.001 | -0.010*** | -0.009*** |  |
|  | (0.001) | (0.001) | (0.001) |  | (0.003) | (0.003) | (0.002) |  | (0.002) | (0.003) | (0.002) |  |
| Sex at birth  (girl = 1) | 0.040*** | -0.018*** | -0.015*** |  | 0.033*** | -0.049*** | -0.034*** |  | 0.003 | -0.123*** | -0.053*** |  |
|  | (0.004) | (0.004) | (0.003) |  | (0.006) | (0.006) | (0.005) |  | (0.005) | (0.006) | (0.005) |  |
| Race  (non-white = 1) | 0.002 | 0.031*** | 0.013** |  | 0.026*** | 0.035*** | 0.022** |  | 0.018* | 0.028*** | 0.012 t |  |
|  | (0.005) | (0.006) | (0.005) |  | (0.008) | (0.008) | (0.007) |  | (0.007) | (0.008) | (0.007) |  |
| Pubertal  Development | 0.012*** | 0.010*** | 0.008*** |  | 0.015*** | 0.009** | 0.010*** |  | 0.009** | 0.007* | 0.006** |  |
|  | (0.002) | (0.002) | (0.002) |  | (0.003) | (0.003) | (0.002) |  | (0.003) | (0.003) | (0.002) |  |
| Immigrant (born  outside US = 1) | 0.006 | -0.013 | 0.020* |  | -0.012 | -0.017 | 0.009 |  | -0.006 | 0.038t | 0.004 |  |
|  | (0.012) | (0.013) | (0.010) |  | (0.018) | (0.020) | (0.016) |  | (0.018) | (0.020) | (0.016) |  |
| Caregiver's characteristics | | | | | | | | | | | |  |
| Age (in years) | 0.001* | 0.000 | -0.000 |  | 0.000 | 0.001 | -0.001t |  | 0.000 | 0.000 | -0.000 |  |
|  | (0.000) | (0.000) | (0.000) |  | (0.000) | (0.001) | (0.000) |  | (0.000) | (0.001) | (0.000) |  |
| Sex at birth  (female = 1) | -0.010 | -0.015* | 0.014* |  | -0.019* | -0.002 | 0.005 |  | -0.012 | 0.011 | 0.020* |  |
|  | (0.006) | (0.007) | (0.005) |  | (0.009) | (0.010) | (0.008) |  | (0.009) | (0.010) | (0.008) |  |
| Race  (non-white = 1) | -0.016** | -0.005 | 0.001 |  | -0.008 | 0.024* | 0.026** |  | -0.019* | 0.029** | 0.029*** |  |
|  | (0.006) | (0.006) | (0.005) |  | (0.009) | (0.010) | (0.008) |  | (0.009) | (0.010) | (0.008) |  |
| Immigrant (born  outside US = 1) | 0.016** | -0.003 | -0.005 |  | -0.011 | -0.006 | -0.028*** |  | -0.010 | -0.018t | -0.037*** |  |
|  | (0.006) | (0.006) | (0.005) |  | (0.009) | (0.009) | (0.008) |  | (0.008) | (0.009) | (0.008) |  |
| Depressive  Symptoms | 0.044*** | 0.029*** | 0.030*** |  | 0.022*** | 0.019*** | 0.014*** |  | 0.045*** | 0.033*** | 0.041*** |  |
|  | (0.002) | (0.002) | (0.002) |  | (0.003) | (0.003) | (0.003) |  | (0.003) | (0.003) | (0.003) |  |
|  |  |  |  |  |  |  |  |  |  |  |  |  |
|  |  |  |  |  |  |  |  |  |  |  |  |  |
| (Continues in the next page) | | | | | | | | | | | |  |
|  |  |  |  |  |  |  |  |  |  |  |  |  |
|  |  |  |  |  |  |  |  |  |  |  |  |  |
| Education level |  |  |  |  |  |  |  |  |  |  |  |  |
| Some College | 0.009 | 0.006 | -0.004 |  | -0.017 | -0.018 | -0.015 |  | -0.022* | -0.044*** | -0.038*** |  |
|  | (0.007) | (0.008) | (0.006) |  | (0.011) | (0.012) | (0.010) |  | (0.011) | (0.012) | (0.010) |  |
| Associate  Degree | 0.002 | 0.006 | -0.008 |  | -0.021t | -0.008 | -0.025* |  | -0.024* | -0.037** | -0.029** |  |
|  | (0.008) | (0.008) | (0.007) |  | (0.012) | (0.012) | (0.010) |  | (0.011) | (0.012) | (0.010) |  |
| College | -0.001 | -0.016* | -0.013* |  | -0.060*** | -0.049*** | -0.046*** |  | -0.036*** | -0.067*** | -0.059*** |  |
|  | (0.007) | (0.007) | (0.006) |  | (0.010) | (0.011) | (0.009) |  | (0.010) | (0.011) | (0.009) |  |
| Masters or more | -0.000 | -0.016* | -0.015* |  | -0.053*** | -0.064*** | -0.050*** |  | -0.027** | -0.074*** | -0.060*** |  |
|  | (0.007) | (0.008) | (0.006) |  | (0.011) | (0.011) | (0.009) |  | (0.010) | (0.012) | (0.010) |  |
| Social environments | | | | | | | | | | | |  |
| Parental warmth | -0.006** | -0.015*** | -0.011*** |  | -0.009** | -0.013*** | -0.012*** |  | -0.001 | -0.006t | -0.013*** |  |
|  | (0.002) | (0.002) | (0.002) |  | (0.003) | (0.003) | (0.003) |  | (0.003) | (0.003) | (0.003) |  |
| Prosocial school  Environment | -0.013*** | -0.015*** | -0.007*** |  | -0.026*** | -0.018*** | -0.021*** |  | -0.016*** | -0.013*** | -0.009*** |  |
|  | (0.002) | (0.002) | (0.002) |  | (0.003) | (0.003) | (0.003) |  | (0.003) | (0.003) | (0.003) |  |
| Family conflict | 0.014*** | 0.022*** | 0.026*** |  | 0.023*** | 0.025*** | 0.033*** |  | 0.007* | 0.013*** | 0.013*** |  |
|  | (0.002) | (0.002) | (0.002) |  | (0.003) | (0.003) | (0.002) |  | (0.003) | (0.003) | (0.002) |  |
| Neighborhood  Deprivation | 0.004 t | 0.010*** | 0.009*** |  | 0.005 | 0.006 | 0.011*** |  | 0.004 | 0.011** | 0.012*** |  |
|  | (0.002) | (0.003) | (0.002) |  | (0.003) | (0.004) | (0.003) |  | (0.004) | (0.004) | (0.003) |  |
|  |  |  |  |  |  |  |  |  |  |  |  |  |
|  |  |  |  |  |  |  |  |  |  |  |  |  |
| Ave. outcome | 0.306 | 0.456 | 0.307 |  | 0.329 | 0.459 | 0.281 |  | 0.306 | 0.358 | 0.229 |  |
| N research sites | 21 | 21 | 21 |  | 21 | 21 | 21 |  | 21 | 21 | 21 |  |
| N families | 8,817 | 8,783 | 8,818 |  | 6,266 | 6,155 | 6,262 |  | 6,422 | 6,457 | 6,450 |  |
| N person-wave dyads | 28,064 | 27,135 | 27,878 |  | 11,006 | 10,682 | 10,992 |  | 11,467 | 11,518 | 11,518 |  |
|  |  |  |  |  |  |  |  |  |  |  |  |  |
|  |  |  |  |  |  |  |  |  |  |  |  |  |
| INT: Internalizing; INA: Inattention; EXT: Externalizing; Ave.: Average; N: Number | | | | | | | | | | | |  |
| **Notes**: Each column represents an independent model. All models were fitted using multilevel mixed-effects linear regression and the estimations were performed by maximum likelihood using Stata 19 and the command MIXED. *** p<0.001, ** p<0.01, * p<0.05, t <0.10. | | | | | | | | | | | |  |
|  |  |  |  |  |  |  |  |  |  |  |  |  |

**Table S9.** Predictors of exact mismatches and changes over time (domains x reporters-level data)

|  | Mental health domains | | | |  |
| --- | --- | --- | --- | --- | --- |
|  | INT | INA | EXT | All |  |
|  | (1) | (2) | (3) | (4) |  |
|  |  |  |  |  |  |
| Reporters | | | | |  |
| Caregivers | -0.022 | -0.010 | -0.026 | -0.022 |  |
|  | (0.029) | (0.032) | (0.025) | (0.018) |  |
| Teachers | 0.052 t | 0.131*** | 0.022 | 0.066*** |  |
|  | (0.028) | (0.031) | (0.024) | (0.018) |  |
| Caregivers x youth's age | 0.004 t | 0.002 | 0.000 | 0.002 |  |
|  | (0.002) | (0.003) | (0.002) | (0.002) |  |
| Teachers x youth's age | -0.004 t | -0.019*** | -0.008*** | -0.010*** |  |
|  | (0.002) | (0.003) | (0.002) | (0.002) |  |
| Youth's characteristics | | | | |  |
| Age (in years) | 0.004** | 0.010*** | -0.002 | 0.004*** |  |
|  | (0.001) | (0.001) | (0.001) | (0.001) |  |
| Sex at birth (girl = 1) | 0.032*** | -0.045*** | -0.025*** | -0.011*** |  |
|  | (0.003) | (0.003) | (0.003) | (0.002) |  |
| Race (non-white = 1) | 0.009t | 0.030*** | 0.014** | 0.017*** |  |
|  | (0.005) | (0.005) | (0.004) | (0.004) |  |
| Pubertal development | 0.011*** | 0.009*** | 0.007*** | 0.008*** |  |
|  | (0.002) | (0.002) | (0.001) | (0.001) |  |
| Immigrant (born outside US = 1) | 0.005 | -0.004 | 0.016t | 0.006 |  |
|  | (0.011) | (0.011) | (0.010) | (0.008) |  |
| Caregiver's characteristics | | | | |  |
| Age (in years) | 0.001* | 0.000 | -0.000 | 0.000 |  |
|  | (0.000) | (0.000) | (0.000) | (0.000) |  |
| Sex at birth (female = 1) | -0.011* | -0.009 | 0.014** | -0.002 |  |
|  | (0.006) | (0.006) | (0.005) | (0.004) |  |
| Race (non-white = 1) | -0.016** | 0.005 | 0.009t | 0.001 |  |
|  | (0.005) | (0.006) | (0.005) | (0.004) |  |
| Immigrant (born outside US = 1) | 0.007 | -0.005 | -0.012* | -0.004 |  |
|  | (0.005) | (0.006) | (0.005) | (0.004) |  |
| Depressive symptoms | 0.040*** | 0.028*** | 0.030*** | 0.033*** |  |
|  | (0.002) | (0.002) | (0.002) | (0.001) |  |
|  |  |  |  |  |  |
|  |  |  |  |  |  |
|  |  |  |  |  |  |
|  |  |  |  |  |  |
|  |  |  |  |  |  |
|  |  |  |  |  |  |
|  |  |  |  |  |  |
|  |  |  |  |  |  |
|  |  |  |  |  |  |
| (Continues in the next page) | | | | |  |
|  |  |  |  |  |  |
|  |  |  |  |  |  |
|  |  |  |  |  |  |
| Education level |  |  |  |  |  |
| Some College | -0.002 | -0.005 | -0.011t | -0.006 |  |
|  | (0.007) | (0.007) | (0.006) | (0.005) |  |
| Associate Degree | -0.007 | -0.002 | -0.014* | -0.008 |  |
|  | (0.007) | (0.007) | (0.006) | (0.005) |  |
| College | -0.019** | -0.029*** | -0.025*** | -0.024*** |  |
|  | (0.006) | (0.007) | (0.006) | (0.005) |  |
| Masters or more | -0.017* | -0.033*** | -0.029*** | -0.026*** |  |
|  | (0.007) | (0.007) | (0.006) | (0.005) |  |
| Social environments | | | | |  |
| Parental warmth | -0.007*** | -0.013*** | -0.012*** | -0.011*** |  |
|  | (0.002) | (0.002) | (0.002) | (0.001) |  |
| Prosocial school environment | -0.014*** | -0.014*** | -0.010*** | -0.012*** |  |
|  | (0.002) | (0.002) | (0.001) | (0.001) |  |
| Family conflict | 0.013*** | 0.020*** | 0.022*** | 0.017*** |  |
|  | (0.002) | (0.002) | (0.001) | (0.001) |  |
| Neighborhood deprivation | 0.004 t | 0.010*** | 0.011*** | 0.008*** |  |
|  | (0.002) | (0.003) | (0.002) | (0.002) |  |
|  |  |  |  |  |  |
|  |  |  |  |  |  |
| Average outcome | 0.311 | 0.434 | 0.284 | 0.342 |  |
| Number of observations | 50,537 | 49,335 | 50,388 | 150,260 |  |
|  |  |  |  |  |  |
|  |  |  |  |  |  |
| INT: Internalizing; INA: Inattention; EXT: Externalizing; Ave.: Average; N: Number | | | | |  |
| **Notes**: Each column represents an independent model. All models were fitted using multilevel mixed-effects linear regression and the estimations were performed by maximum likelihood using Stata 19 and the command MIXED. *** p<0.001, ** p<0.01, * p<0.05, t <0.10. | | | | |  |
|  |  |  |  |  |  |
|  |  |  |  |  |  |

**Figure S2.** Exact mismatches over time, by youth’s age


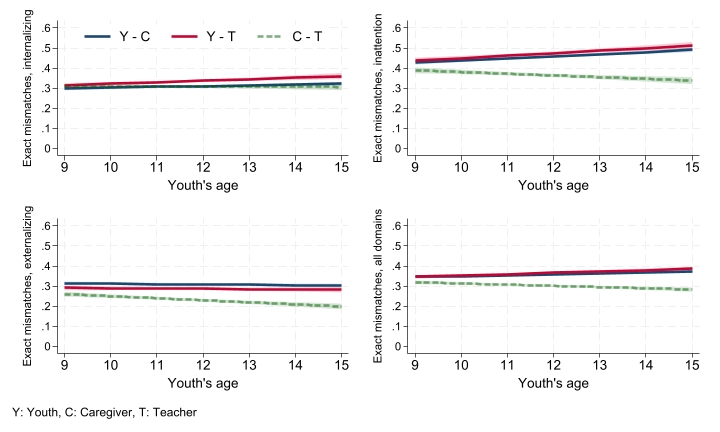


## **(b) Presence versus absence mismatches**

We also examined mismatches based on symptom presence versus absence. The presence versus absence mismatch was defined as one reporter indicating symptom presence while the other indicated absence. The results using this approach revealed similar discrepancies. Youth-caregiver mismatches based on symptom presence were 27% for internalizing, 39% for inattention, and 27% for externalizing symptoms. Youth-teacher mismatches were slightly higher, with rates of 30% for internalizing, 40% for inattention, and 26% for externalizing symptoms. Caregiver-teacher mismatches were lower overall, observed in 28% of internalizing, 30% of inattention, and 20% of externalizing symptoms (see **Table S10**).

Replicating Research Aim 2 with presence–absence mismatches as the outcome yielded broadly consistent results (see **Table S11**). Across domains and reporter pairs, mismatch rates were higher when caregivers reported more depressive symptoms and when youths experienced more family conflict, while parental warmth and a more prosocial school environment were associated with lower mismatch rates. Pubertal development was also consistently associated with greater mismatch. Several demographic patterns replicated those observed for exact mismatches: mismatch rates tended to be higher in the inattention domain and varied by youth sex and race/ethnicity in domain-specific ways (e.g., higher mismatch for girls in internalizing but lower mismatch for girls in externalizing, particularly for youth–adult comparisons). Higher caregiver education was generally associated with lower mismatch rates, especially in comparisons involving teacher reports.

Presence–absence mismatches also showed systematic developmental patterning (see **Table S12**, and **Figure S3**). Across internalizing and inattention, youth–adult mismatches (youth–caregiver and youth–teacher) increased with youth age, indicating that youths’ endorsement of symptom presence becomes progressively less aligned with adult observers across early adolescence. In contrast, caregiver–teacher mismatches were comparatively stable and tended to be flat or slightly declining with age, particularly for inattention. For externalizing symptoms, age-related changes were smaller and, if anything, showed flatter or slightly declining youth–adult mismatches, consistent with externalizing behaviors being more overt and therefore more consistently detected across observers. Overall, these results reinforce the robustness of the main conclusions: reporter discrepancies are substantial under a presence–absence definition and become more pronounced across adolescence primarily for youth–adult comparisons, especially for inattention.

**Table S10.** Presence vs absence mismatches, by reporters and mental health domains

|  | Presence vs absence mismatches in symptoms, by reporters | | |
| --- | --- | --- | --- |
|  | Youth - Caregiver | Youth - Teacher | Caregiver - Teacher |
|  | (1) | (2) | (3) |
|  | | | |
| Internalizing symptoms | 27.085% | 29.794% | 28.258% |
| Inattention symptoms | 39.168% | 39.119% | 30.207% |
| Externalizing symptoms | 27.243% | 25.728% | 20.363% |
|  | | | |
|  | | | |
| **Notes:** Results based on the BPM-Y, BPM-T, and CBCL, which are available for all participants from baseline to the three-year follow-up for the caregiver and teacher, and from the one-year follow-up to the three-year follow-up for the youth. We focused our analysis on data from one- to three-year follow-up. Exact mismatches in Column (1) are based on around 30,000 observations, and differences in reported in Columns (2) and (3) are based on around 12,000 observations. | | | |

**Table S11.** Predictors of reporters’ presence versus absence mismatches

|  | Youth - Caregiver | | |  | Youth – Teacher | | |  | Caregiver - Teacher | | |  |
| --- | --- | --- | --- | --- | --- | --- | --- | --- | --- | --- | --- | --- |
|  | INT | INA | EXT |  | INT | INA | EXT |  | INT | INA | EXT |  |
|  | (1) | (2) | (3) |  | (4) | (5) | (6) |  | (7) | (8) | (9) |  |
|  |  |  |  |  |  |  |  |  |  |  |  |  |
| Youth's characteristics | | | | | | | | | | | |  |
| Age (in years) | -0.000 | 0.009*** | -0.003* |  | 0.007** | 0.011*** | 0.003 |  | 0.001 | -0.003 | -0.006** |  |
|  | (0.001) | (0.001) | (0.001) |  | (0.002) | (0.003) | (0.002) |  | (0.002) | (0.002) | (0.002) |  |
| Sex at birth  (girl = 1) | 0.023*** | -0.001 | -0.013*** |  | 0.024*** | -0.013* | -0.024*** |  | 0.000 | -0.076*** | -0.040*** |  |
|  | (0.003) | (0.004) | (0.003) |  | (0.005) | (0.006) | (0.004) |  | (0.005) | (0.005) | (0.004) |  |
| Race  (non-white = 1) | 0.001 | 0.020*** | 0.008t |  | 0.019** | 0.019* | 0.013* |  | 0.017* | 0.010 | 0.005 |  |
|  | (0.005) | (0.005) | (0.004) |  | (0.007) | (0.008) | (0.006) |  | (0.007) | (0.007) | (0.006) |  |
| Pubertal  Development | 0.009*** | 0.008*** | 0.006*** |  | 0.011*** | 0.009** | 0.008*** |  | 0.007** | 0.004 | 0.006* |  |
|  | (0.002) | (0.002) | (0.001) |  | (0.003) | (0.003) | (0.002) |  | (0.003) | (0.003) | (0.002) |  |
| Immigrant (born  outside US = 1) | 0.004 | -0.014 | 0.019* |  | -0.018 | -0.014 | 0.011 |  | -0.009 | 0.035* | 0.001 |  |
|  | (0.010) | (0.012) | (0.009) |  | (0.017) | (0.019) | (0.015) |  | (0.016) | (0.018) | (0.015) |  |
| Caregiver's characteristics | | | | | | | | | | | |  |
| Age (in years) | 0.001** | -0.000 | 0.000 |  | 0.000 | 0.000 | -0.001* |  | 0.000 | -0.000 | -0.000 |  |
|  | (0.000) | (0.000) | (0.000) |  | (0.000) | (0.000) | (0.000) |  | (0.000) | (0.000) | (0.000) |  |
| Sex at birth  (female = 1) | -0.009 | -0.024*** | 0.007 |  | -0.014 t | -0.007 | 0.002 |  | -0.014t | -0.003 | 0.012 |  |
|  | (0.006) | (0.006) | (0.005) |  | (0.008) | (0.009) | (0.007) |  | (0.008) | (0.009) | (0.007) |  |
| Race  (non-white = 1) | -0.006 | 0.006 | 0.010* |  | -0.003 | 0.011 | 0.020** |  | -0.015t | 0.034*** | 0.028*** |  |
|  | (0.005) | (0.006) | (0.005) |  | (0.009) | (0.009) | (0.007) |  | (0.008) | (0.009) | (0.007) |  |
| Immigrant (born  outside US = 1) | 0.018*** | 0.006 | -0.000 |  | -0.008 | 0.016t | -0.016* |  | -0.006 | -0.001 | -0.030*** |  |
|  | (0.005) | (0.006) | (0.005) |  | (0.008) | (0.009) | (0.007) |  | (0.008) | (0.008) | (0.007) |  |
| Depressive  Symptoms | 0.031*** | 0.007*** | 0.017*** |  | 0.017*** | 0.007* | 0.011*** |  | 0.033*** | 0.016*** | 0.034*** |  |
|  | (0.002) | (0.002) | (0.002) |  | (0.003) | (0.003) | (0.002) |  | (0.003) | (0.003) | (0.002) |  |
|  |  |  |  |  |  |  |  |  |  |  |  |  |
|  |  |  |  |  |  |  |  |  |  |  |  |  |
| (Continues in the next page) | | | | | | | | | | | |  |
|  |  |  |  |  |  |  |  |  |  |  |  |  |
|  |  |  |  |  |  |  |  |  |  |  |  |  |
| Education level |  |  |  |  |  |  |  |  |  |  |  |  |
| Some College | 0.006 | -0.001 | -0.006 |  | -0.016 | -0.005 | -0.004 |  | -0.022* | -0.042*** | -0.041*** |  |
|  | (0.006) | (0.007) | (0.006) |  | (0.010) | (0.011) | (0.009) |  | (0.010) | (0.011) | (0.009) |  |
| Associate Degree | -0.004 | 0.004 | -0.012* |  | -0.023* | 0.014 | -0.013 |  | -0.025* | -0.028* | -0.035*** |  |
|  | (0.007) | (0.008) | (0.006) |  | (0.011) | (0.012) | (0.009) |  | (0.010) | (0.011) | (0.009) |  |
| College | -0.005 | -0.014* | -0.011* |  | -0.052*** | -0.016 | -0.024** |  | -0.034*** | -0.048*** | -0.050*** |  |
|  | (0.006) | (0.007) | (0.005) |  | (0.009) | (0.010) | (0.008) |  | (0.009) | (0.010) | (0.008) |  |
| Masters or more | -0.006 | -0.011 | -0.013* |  | -0.047*** | -0.024* | -0.030*** |  | -0.027** | -0.047*** | -0.053*** |  |
|  | (0.006) | (0.007) | (0.006) |  | (0.010) | (0.011) | (0.009) |  | (0.010) | (0.010) | (0.009) |  |
| Social environments | | | | | | | | | | | |  |
| Parental warmth | -0.005** | -0.009*** | -0.007*** |  | -0.006* | -0.006t | -0.009*** |  | 0.000 | -0.003 | -0.009*** |  |
|  | (0.002) | (0.002) | (0.002) |  | (0.003) | (0.003) | (0.002) |  | (0.003) | (0.003) | (0.002) |  |
| Prosocial school  Environment | -0.009*** | -0.008*** | -0.005** |  | -0.020*** | -0.008** | -0.015*** |  | -0.013*** | -0.007* | -0.006* |  |
|  | (0.002) | (0.002) | (0.002) |  | (0.003) | (0.003) | (0.002) |  | (0.003) | (0.003) | (0.002) |  |
| Family conflict | 0.011*** | 0.012*** | 0.018*** |  | 0.019*** | 0.017*** | 0.029*** |  | 0.004t | 0.008** | 0.012*** |  |
|  | (0.002) | (0.002) | (0.002) |  | (0.003) | (0.003) | (0.002) |  | (0.003) | (0.003) | (0.002) |  |
| Neighborhood  Deprivation | 0.005* | 0.007** | 0.007*** |  | 0.003 | 0.004 | 0.009** |  | 0.002 | 0.009* | 0.011*** |  |
|  | (0.002) | (0.002) | (0.002) |  | (0.003) | (0.003) | (0.003) |  | (0.003) | (0.004) | (0.003) |  |
|  |  |  |  |  |  |  |  |  |  |  |  |  |
|  |  |  |  |  |  |  |  |  |  |  |  |  |
| Ave. outcome | 0.276 | 0.393 | 0.273 |  | 0.302 | 0.394 | 0.260 |  | 0.283 | 0.302 | 0.209 |  |
| N research sites | 21 | 21 | 21 |  | 21 | 21 | 21 |  | 21 | 21 | 21 |  |
| N families | 8,817 | 8,783 | 8,818 |  | 6,266 | 6,155 | 6,262 |  | 6,422 | 6,457 | 6,450 |  |
| N person-wave dyads | 28,064 | 27,135 | 27,878 |  | 11,006 | 10,682 | 10,992 |  | 11,467 | 11,518 | 11,518 |  |
|  |  |  |  |  |  |  |  |  |  |  |  |  |
|  |  |  |  |  |  |  |  |  |  |  |  |  |
| INT: Internalizing; INA: Inattention; EXT: Externalizing; Ave.: Average; N: Number | | | | | | | | | | | |  |
| **Notes**: Each column represents an independent model. All models were fitted using multilevel mixed-effects linear regression and the estimations were performed by maximum likelihood using Stata 19 and the command MIXED. *** p<0.001, ** p<0.01, * p<0.05, t <0.10. | | | | | | | | | | | |  |
|  |  |  |  |  |  |  |  |  |  |  |  |  |

**Table S12.** Predictors of presence versus absence mismatches and changes over time (domains x reporters-level data)

|  | Mental health domains | | | |  |
| --- | --- | --- | --- | --- | --- |
|  | INT | INA | EXT | All |  |
|  | (1) | (2) | (3) | (4) |  |
|  |  |  |  |  |  |
| Reporters | | | | |  |
| Caregivers | -0.045 | -0.028 | -0.051* | -0.043* |  |
|  | (0.028) | (0.031) | (0.024) | (0.018) |  |
| Teachers | 0.005 | 0.055t | -0.011 | 0.015 |  |
|  | (0.027) | (0.031) | (0.024) | (0.017) |  |
| Caregivers x youth's age | 0.006** | 0.003 | 0.004t | 0.005** |  |
|  | (0.002) | (0.003) | (0.002) | (0.001) |  |
| Teachers x youth's age | 0.000 | -0.012*** | -0.004* | -0.005*** |  |
|  | (0.002) | (0.003) | (0.002) | (0.001) |  |
| Youth's characteristics | | | | |  |
| Age (in years) | 0.000 | 0.009*** | -0.003* | 0.002** |  |
|  | (0.001) | (0.001) | (0.001) | (0.001) |  |
| Sex at birth (girl = 1) | 0.019*** | -0.019*** | -0.020*** | -0.005* |  |
|  | (0.003) | (0.003) | (0.003) | (0.002) |  |
| Race (non-white = 1) | 0.007t | 0.018*** | 0.008* | 0.010*** |  |
|  | (0.004) | (0.005) | (0.004) | (0.003) |  |
| Pubertal development | 0.008*** | 0.007*** | 0.006*** | 0.007*** |  |
|  | (0.001) | (0.002) | (0.001) | (0.001) |  |
| Immigrant (born outside US = 1) | 0.001 | -0.004 | 0.016t | 0.004 |  |
|  | (0.009) | (0.010) | (0.008) | (0.007) |  |
| Caregiver's characteristics | | | | |  |
| Age (in years) | 0.001* | -0.000 | -0.000 | 0.000 |  |
|  | (0.000) | (0.000) | (0.000) | (0.000) |  |
| Sex at birth (female = 1) | -0.010* | -0.017** | 0.008t | -0.006t |  |
|  | (0.005) | (0.005) | (0.004) | (0.003) |  |
| Race (non-white = 1) | -0.009t | 0.012* | 0.014** | 0.007* |  |
|  | (0.005) | (0.005) | (0.004) | (0.003) |  |
| Immigrant (born outside US = 1) | 0.010* | 0.007 | -0.007t | 0.003 |  |
|  | (0.005) | (0.005) | (0.004) | (0.003) |  |
| Depressive symptoms | 0.029*** | 0.009*** | 0.019*** | 0.019*** |  |
|  | (0.002) | (0.002) | (0.001) | (0.001) |  |
|  |  |  |  |  |  |
|  |  |  |  |  |  |
|  |  |  |  |  |  |
| (Continues in the next page) | | | | |  |
|  |  |  |  |  |  |
| Education level |  |  |  |  |  |
| Some College | -0.003 | -0.007 | -0.011* | -0.007t |  |
|  | (0.006) | (0.006) | (0.005) | (0.004) |  |
| Associate Degree | -0.011t | 0.001 | -0.016** | -0.009* |  |
|  | (0.006) | (0.007) | (0.005) | (0.004) |  |
| College | -0.020*** | -0.020** | -0.020*** | -0.020*** |  |
|  | (0.006) | (0.006) | (0.005) | (0.004) |  |
| Masters or more | -0.019*** | -0.019** | -0.023*** | -0.021*** |  |
|  | (0.006) | (0.006) | (0.005) | (0.004) |  |
| Social environments | | | | |  |
| Parental warmth | -0.005** | -0.008*** | -0.008*** | -0.007*** |  |
|  | (0.002) | (0.002) | (0.001) | (0.001) |  |
| Prosocial school environment | -0.011*** | -0.008*** | -0.007*** | -0.009*** |  |
|  | (0.002) | (0.002) | (0.001) | (0.001) |  |
| Family conflict | 0.010*** | 0.012*** | 0.017*** | 0.012*** |  |
|  | (0.001) | (0.002) | (0.001) | (0.001) |  |
| Neighborhood deprivation | 0.004* | 0.007*** | 0.009*** | 0.007*** |  |
|  | (0.002) | (0.002) | (0.002) | (0.001) |  |
|  |  |  |  |  |  |
|  |  |  |  |  |  |
| Average outcome | 0.284 | 0.372 | 0.256 | 0.303 |  |
| Number of observations | 50,537 | 49,335 | 50,388 | 150,260 |  |
|  |  |  |  |  |  |
|  |  |  |  |  |  |
| INT: Internalizing; INA: Inattention; EXT: Externalizing; Ave.: Average; N: Number | | | | |  |
| **Notes**: Each column represents an independent model. All models were fitted using multilevel mixed-effects linear regression and the estimations were performed by maximum likelihood using Stata 19 and the command MIXED. *** p<0.001, ** p<0.01, * p<0.05, t <0.10. | | | | |  |
|  |  |  |  |  |  |
|  |  |  |  |  |  |

**Figure S3.** Presence versus absence mismatches over time, by youth’s age


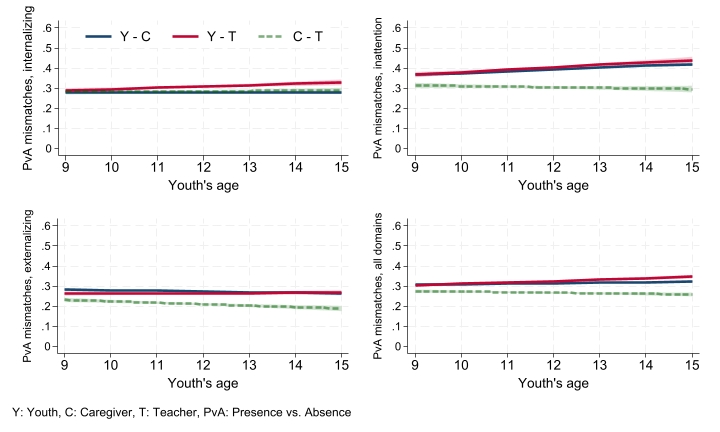


## **(c) Predictors of absolute discrepancies, by domains and reporters**

Overall findings were consistent with the main analyses of mean-level discrepancies (see **Table S13**). Youth age was associated with larger absolute discrepancies primarily in the inattention domain for youth–caregiver (b = 0.079, p < .001) and youth–teacher comparisons (b = 0.076, p < .001). Age-related increases were also evident for youth–teacher internalizing discrepancies (b = 0.079, p < .001), whereas youth–caregiver internalizing discrepancies increased only modestly (b = 0.019, p < .10). In contrast, caregiver–teacher absolute discrepancies decreased with youth age for inattention (b = −0.071, p < .001) and externalizing (b = −0.063, p < .001), suggesting modest convergence between adult observers over development in these domains.

Sex differences were domain-specific. Girls showed larger absolute discrepancies for internalizing in youth–caregiver (b = 0.291, p < .001) and youth–teacher comparisons (b = 0.267, p < .001), but smaller absolute discrepancies for inattention and externalizing across most reporter pairs (e.g., b = −0.089 to −0.364 for inattention and b = −0.089 to −0.370 for externalizing, all p’s < .01). Non-white youth generally exhibited larger absolute discrepancies, particularly for inattention and for comparisons involving teachers (e.g., youth–teacher inattention: b = 0.314, p < .001). Pubertal development was also positively associated with absolute discrepancies across most domains and reporter pairs.

Caregiver depressive symptoms robustly predicted larger absolute discrepancies across all reporter pairs and symptom domains (all p’s < .001). Higher caregiver education was consistently associated with smaller absolute discrepancies, particularly for inattention and externalizing and in comparisons involving teacher reports (most p’s < .001). Regarding socio-experiential environments, family conflict was associated with larger absolute discrepancies across all domains and reporter pairs (all p’s < .01), whereas parental warmth and a prosocial school environment were associated with smaller absolute discrepancies across most comparisons (generally p’s < .01). Neighborhood deprivation showed small, generally positive associations with absolute discrepancies, with stronger and more consistent effects for youth–caregiver comparisons than for youth–teacher comparisons.

**Table S13.** Predictors of absolute discrepancies, by domains and reporters

|  | Youth - Caregiver | | |  | Youth – Teacher | | |  | Caregiver - Teacher | | |  |
| --- | --- | --- | --- | --- | --- | --- | --- | --- | --- | --- | --- | --- |
|  | INT | INA | EXT |  | INT | INA | EXT |  | INT | INA | EXT |  |
|  | (1) | (2) | (3) |  | (4) | (5) | (6) |  | (7) | (8) | (9) |  |
|  |  |  |  |  |  |  |  |  |  |  |  |  |
| Youth's characteristics | | | | | | | | | | | |  |
| Age (in years) | 0.019t | 0.079*** | -0.008 |  | 0.079*** | 0.076*** | 0.014 |  | 0.001 | -0.071*** | -0.063*** |  |
|  | (0.010) | (0.011) | (0.008) |  | (0.019) | (0.021) | (0.016) |  | (0.017) | (0.019) | (0.015) |  |
| Sex at birth  (girl = 1) | 0.291*** | -0.089** | -0.089*** |  | 0.267*** | -0.364*** | -0.209*** |  | 0.010 | -0.900*** | -0.370*** |  |
|  | (0.027) | (0.031) | (0.024) |  | (0.042) | (0.046) | (0.037) |  | (0.038) | (0.044) | (0.036) |  |
| Race  (non-white = 1) | 0.039 | 0.247*** | 0.114** |  | 0.195*** | 0.314*** | 0.186*** |  | 0.133* | 0.227*** | 0.103* |  |
|  | (0.040) | (0.046) | (0.036) |  | (0.058) | (0.065) | (0.052) |  | (0.054) | (0.062) | (0.052) |  |
| Pubertal  Development | 0.086*** | 0.094*** | 0.055*** |  | 0.131*** | 0.076** | 0.075*** |  | 0.071*** | 0.036 | 0.038* |  |
|  | (0.013) | (0.015) | (0.012) |  | (0.022) | (0.024) | (0.019) |  | (0.019) | (0.022) | (0.018) |  |
| Immigrant (born  outside US = 1) | 0.038 | -0.121 | 0.133t |  | -0.034 | -0.224 | 0.061 |  | -0.044 | 0.182 | -0.016 |  |
|  | (0.087) | (0.098) | (0.077) |  | (0.140) | (0.155) | (0.123) |  | (0.127) | (0.147) | (0.120) |  |
| Caregiver's characteristics | | | | | | | | | | | |  |
| Age (in years) | 0.003 | -0.001 | -0.002 |  | 0.003 | 0.003 | -0.006t |  | 0.002 | 0.005 | -0.003 |  |
|  | (0.002) | (0.003) | (0.002) |  | (0.004) | (0.004) | (0.003) |  | (0.003) | (0.004) | (0.003) |  |
| Sex at birth  (female = 1) | -0.096* | -0.103* | 0.091* |  | -0.171* | 0.023 | 0.028 |  | -0.105t | 0.098 | 0.135* |  |
|  | (0.046) | (0.052) | (0.041) |  | (0.070) | (0.077) | (0.061) |  | (0.064) | (0.073) | (0.060) |  |
| Race  (non-white = 1) | -0.066 | 0.010 | 0.066t |  | -0.058 | 0.243** | 0.238*** |  | -0.128* | 0.332*** | 0.249*** |  |
|  | (0.044) | (0.050) | (0.039) |  | (0.071) | (0.079) | (0.063) |  | (0.065) | (0.075) | (0.061) |  |
| Immigrant (born  outside US = 1) | 0.108* | -0.066 | -0.082* |  | -0.059 | -0.051 | -0.222*** |  | -0.057 | -0.151* | -0.298*** |  |
|  | (0.042) | (0.049) | (0.038) |  | (0.066) | (0.074) | (0.059) |  | (0.061) | (0.071) | (0.058) |  |
| Depressive  Symptoms | 0.305*** | 0.206*** | 0.208*** |  | 0.172*** | 0.153*** | 0.121*** |  | 0.322*** | 0.221*** | 0.289*** |  |
|  | (0.015) | (0.017) | (0.013) |  | (0.023) | (0.025) | (0.020) |  | (0.021) | (0.024) | (0.020) |  |
| Education level |  |  |  |  |  |  |  |  |  |  |  |  |
| Some College | 0.044 | 0.033 | -0.080t |  | -0.190* | -0.151 | -0.115 |  | -0.207** | -0.314*** | -0.290*** |  |
|  | (0.053) | (0.060) | (0.047) |  | (0.085) | (0.093) | (0.074) |  | (0.077) | (0.088) | (0.073) |  |
| Associate  Degree | -0.010 | 0.014 | -0.106* |  | -0.229** | -0.087 | -0.180* |  | -0.216** | -0.265** | -0.257*** |  |
|  | (0.056) | (0.063) | (0.050) |  | (0.089) | (0.097) | (0.078) |  | (0.080) | (0.092) | (0.076) |  |
| College | -0.062 | -0.199*** | -0.161*** |  | -0.489*** | -0.437*** | -0.387*** |  | -0.297*** | -0.523*** | -0.473*** |  |
|  | (0.051) | (0.058) | (0.045) |  | (0.079) | (0.087) | (0.070) |  | (0.072) | (0.083) | (0.069) |  |
| Masters or more | -0.056 | -0.218*** | -0.180*** |  | -0.450*** | -0.541*** | -0.416*** |  | -0.237** | -0.587*** | -0.485*** |  |
|  | (0.053) | (0.060) | (0.047) |  | (0.082) | (0.091) | (0.073) |  | (0.075) | (0.087) | (0.071) |  |
| Social environments | | | | | | | | | | | |  |
| Parental warmth | -0.047** | -0.103*** | -0.077*** |  | -0.057* | -0.083** | -0.077*** |  | -0.004 | -0.029 | -0.083*** |  |
|  | (0.015) | (0.017) | (0.013) |  | (0.023) | (0.026) | (0.020) |  | (0.021) | (0.024) | (0.019) |  |
| Prosocial school  Environment | -0.092*** | -0.109*** | -0.059*** |  | -0.196*** | -0.128*** | -0.153*** |  | -0.116*** | -0.083*** | -0.066*** |  |
|  | (0.014) | (0.016) | (0.013) |  | (0.023) | (0.025) | (0.020) |  | (0.021) | (0.024) | (0.019) |  |
| Family conflict | 0.111*** | 0.188*** | 0.194*** |  | 0.173*** | 0.218*** | 0.264*** |  | 0.052** | 0.093*** | 0.095*** |  |
|  | (0.014) | (0.016) | (0.012) |  | (0.022) | (0.024) | (0.019) |  | (0.020) | (0.023) | (0.018) |  |
| Neighborhood  Deprivation | 0.036* | 0.081*** | 0.064*** |  | 0.044t | 0.052t | 0.071** |  | 0.030 | 0.087** | 0.084*** |  |
|  | (0.018) | (0.022) | (0.017) |  | (0.025) | (0.030) | (0.026) |  | (0.026) | (0.031) | (0.025) |  |
|  |  |  |  |  |  |  |  |  |  |  |  |  |
|  |  |  |  |  |  |  |  |  |  |  |  |  |
| Ave. outcome | 2.056 | 3.122 | 2.066 |  | 2.237 | 3.204 | 1.959 |  | 2.000 | 2.418 | 1.541 |  |
| N research sites | 21 | 21 | 21 |  | 21 | 21 | 21 |  | 21 | 21 | 21 |  |
| N families | 8,817 | 8,783 | 8,818 |  | 6,266 | 6,155 | 6,262 |  | 6,422 | 6,457 | 6,450 |  |
| N person-wave dyads | 28,064 | 27,135 | 27,878 |  | 11,006 | 10,682 | 10,992 |  | 11,467 | 11,518 | 11,518 |  |
|  |  |  |  |  |  |  |  |  |  |  |  |  |
|  |  |  |  |  |  |  |  |  |  |  |  |  |
| INT: Internalizing; INA: Inattention; EXT: Externalizing; Ave.: Average; N: Number | | | | | | | | | | | |  |
| **Notes**: Each column represents an independent model. All models were fitted using multilevel mixed-effects linear regression and the estimations were performed by maximum likelihood using Stata 19 and the command MIXED. *** p<0.001, ** p<0.01, * p<0.05, t <0.10. | | | | | | | | | | | |  |
|  |  |  |  |  |  |  |  |  |  |  |  |  |

# **Appendix S6**: Multiple imputation

To assess whether incomplete data could affect our conclusions, we conducted two complementary sensitivity checks. First, we described the extent of missing data across outcomes and covariates and tested whether missingness was systematically related to baseline characteristics (Section 6a). Second, we refit the main models using multiple imputation for missing data (Section 6b).

## **(a) Missingness patterns**

We begin by summarizing the percentage of missing values for each discrepancy outcome and each covariate at the 1-, 2-, and 3-year follow-ups using the subsample of youth with at least one assessment between these waves (N = 11,522) (see **Table S14**). Two patterns can be observed in the summary statistics. First, missingness increases across follow-up waves for most variables, consistent with cumulative attrition and declining availability of some reporters over time. Second, missingness is substantially higher for discrepancy outcomes involving teachers than for youth–caregiver discrepancies: youth–teacher and caregiver–teacher discrepancy outcomes are missing for roughly half of observations at the 1-year follow-up and exceed 80% missingness by the 3-year follow-up, whereas youth–caregiver discrepancy outcomes show much lower missingness (generally under ~17% across waves). Missingness in baseline/follow-up covariates is generally modest (roughly 3–11% depending on wave), with neighborhood deprivation showing comparatively higher missingness (about 10–17% across waves).

To examine whether missingness in discrepancy outcomes was systematically related to baseline characteristics, we constructed a youth-level measure of missingness for each reporter dyad (youth–caregiver, youth–teacher, caregiver–teacher). After balancing the person–wave panel across the three follow-up waves (periods 2–4) using the Stata function or command *fillin*, we created wave-level indicators for whether any of the three domain-specific discrepancies within a dyad (internalizing, inattention, externalizing) was missing in that wave. We then averaged these wave-level indicators across periods 2–4 within each youth, yielding a dyad-specific missingness measure interpreted as the proportion of follow-up waves in which discrepancy information for that dyad was missing. Finally, we estimated linear regressions of these missingness measures on baseline youth and caregiver characteristics (youth age, sex, race; caregiver age, sex, race; and caregiver education), clustering standard errors by research site (**Table S15**).

Overall, missingness in discrepancy scores was not random, but its associations with baseline characteristics were modest in magnitude (see **Table S15**). Older youth at baseline had slightly lower proportion of follow-up waves missing (−2.5 percentage points fewer waves missing per year), while age was not significantly related to missingness in the youth–teacher or caregiver–teacher dyads. Non-White youth and non-White caregivers exhibited higher probabilities of missing discrepancy data across dyads (e.g., 4.0 percentage points for youth–caregiver and 8.9 percentage points for youth–teacher for non-White youth). Higher caregiver education was consistently associated with lower missingness, with the largest reductions observed for college and postgraduate education (approximately 7–8 percentage points relative to the high school-or-less reference category). Caregiver sex was also associated with slightly lower missingness for youth–teacher and caregiver–teacher discrepancies. Taken together, these diagnostics indicate selective missingness, but the predictors most strongly related to missingness are observed and are included in the imputation models, which supports the plausibility of a missing-at-random approach and motivates multiple imputation as a sensitivity analysis.

**Table S14.** Missing values, by variable and follow-up

|  | Year-1  follow-up | Year-2  follow-up | Year-3  follow-up | All years |
| --- | --- | --- | --- | --- |
|  | (1) | (2) | (3) | (4) |
|  |  |  |  |  |
| **Youth mental health symptoms** | | | | |
| Internalizing, Y – C | 8.410% | 9.365% | 15.136% | 10.970% |
| Inattention, Y – C | 12.246% | 12.663% | 16.811% | 13.907% |
| Externalizing, Y – C | 9.174% | 9.964% | 15.527% | 11.555% |
| Internalizing, Y – T | 51.319% | 64.199% | 81.548% | 65.689% |
| Inattention, Y – T | 53.281% | 65.049% | 81.783% | 66.704% |
| Externalizing, Y – T | 51.614% | 64.129% | 81.375% | 65.706% |
| Internalizing, C – T | 48.559% | 62.923% | 81.201% | 64.228% |
| Inattention, C – T | 48.698% | 62.480% | 81.036% | 64.072% |
| Externalizing, C – T | 48.455% | 62.671% | 81.062% | 64.063% |
| **Youth characteristics** | | | | |
| Age (in years) | 2.630% | 4.773% | 10.302% | 5.902% |
| Sex at birth (girl = 1) | 2.821% | 4.964% | 10.519% | 6.101% |
| Race (non-white = 1) | 2.838% | 4.973% | 10.528% | 6.113% |
| Pubertal development | 3.394% | 5.520% | 11.031% | 6.648% |
| Immigrant (born outside US = 1) | 2.942% | 5.069% | 10.623% | 6.211% |
| **Caregiver characteristics** | | | | |
| Age (in years, at baseline) | 3.498% | 5.607% | 11.118% | 6.741% |
| Sex at birth (female = 1) | 2.873% | 5.008% | 10.562% | 6.148% |
| Race (non-white = 1) | 3.012% | 5.155% | 10.684% | 6.284% |
| Immigrant (born outside US = 1) | 2.855% | 4.982% | 10.536% | 6.125% |
| Depressive symptoms | 2.847% | 4.982% | 10.536% | 6.122% |
| Primary caregiver's education | 2.951% | 5.103% | 10.623% | 6.226% |
| **Social Environments** | | | | |
| Parental warmth | 3.029% | 5.147% | 10.719% | 6.298% |
| Prosocial school environment | 2.960% | 5.103% | 10.658% | 6.240% |
| Family conflict | 2.960% | 5.103% | 10.658% | 6.240% |
| Neighborhood deprivation | 9.747% | 11.630% | 16.672% | 12.683% |
|  |  |  |  |  |
|  |  |  |  |  |
| Y: Youth; C: Caregiver; T: Teacher  **Notes**: Missing values are computed relative to the total number of youths participating in the ABCD study at least once between year-1 follow-up and year-3 follow-up (N = 11,522) | | | | |

**Table S15.** Missingness patterns by baseline covariates

|  | Missingness on | | |  |
| --- | --- | --- | --- | --- |
|  | Youth - Caregiver | Youth - Teacher | Caregiver - Teacher |  |
|  | (1) | (2) | (3) |  |
|  |  |  |  |  |
| Youth's characteristics | | | |  |
| Age (in years) | -0.025*** | -0.005 | 0.005 |  |
|  | (0.003) | (0.007) | (0.007) |  |
| Sex at birth (girl = 1) | 0.002 | 0.001 | 0.002 |  |
|  | (0.004) | (0.005) | (0.005) |  |
| Race (non-white = 1) | 0.040*** | 0.089*** | 0.080** |  |
|  | (0.009) | (0.018) | (0.021) |  |
| Caregiver's characteristics | | | |  |
| Age (in years) | -0.001 | -0.001 | -0.001 |  |
|  | (0.001) | (0.001) | (0.001) |  |
| Sex at birth (female = 1) | -0.010 | -0.028** | -0.026* |  |
|  | (0.009) | (0.009) | (0.010) |  |
| Race (non-white = 1) | 0.023* | 0.049** | 0.056** |  |
|  | (0.009) | (0.015) | (0.019) |  |
| Education level |  |  |  |  |
| Some College | -0.033* | -0.039*** | -0.037** |  |
|  | (0.013) | (0.009) | (0.011) |  |
| Associate Degree | -0.037** | -0.037** | -0.042** |  |
|  | (0.013) | (0.011) | (0.011) |  |
| College | -0.074*** | -0.077*** | -0.074*** |  |
|  | (0.014) | (0.012) | (0.013) |  |
| Masters or more | -0.079*** | -0.073*** | -0.072*** |  |
|  | (0.014) | (0.013) | (0.013) |  |
|  |  |  |  |  |
|  |  |  |  |  |
| Average outcome | 0.174 | 0.690 | 0.651 |  |
| Number of observations | 11,370 | 11,370 | 11,370 |  |
|  |  |  |  |  |
|  |  |  |  |  |
| INT: Internalizing; INA: Inattention; EXT: Externalizing; Ave.: Average; N: Number | | | |  |
| **Notes**: Each column represents an independent model. All models were fitted using linear regression and clustering the standard errors at the research site level. *** p<0.001, ** p<0.01, * p<0.05, t <0.10. | | | |  |
|  |  |  |  |  |

## **(b) Implementing Multiple Imputation**

To test the robustness of results to missing values, we used Stata 19’s mi impute chained with 10 imputations under a missing-at-random assumption, including all discrepancy outcomes and the full set of youth, caregiver, and social-environment covariates in the imputation models (continuous variables imputed with linear regression; caregiver education with ordered logit). or Research Aims 1 and 2, we imputed each reporter-by-domain discrepancy outcome in turn and then re-estimated: (a) mean discrepancies and standardized mean differences for all nine reporter-by-domain combinations, and (b) the multilevel mixed-effects models predicting discrepancies from youth, caregiver, and contextual characteristics. For Research Aim 3, we re-estimated the reporter-by-domain panel model of symptoms over age using multiple imputation for the symptom scales and covariates.

The results are highly consistent with our original complete-case findings. For the mean discrepancies, the results were very similar estimates (**Table 2** in the main manuscript and **Table S16**). For example, the youth–caregiver internalizing discrepancy is 0.411 (SMD = 0.168) using the multiple imputation approach compared to 0.405 (SMD = 0.165) in the complete-case analysis. Magnitudes across all reporters and domains are on the order of 0.01–0.03 points (typically <0.03 SD units), with identical directions and patterns of statistical significance.

For the predictors of discrepancies, coefficients and standard errors are likewise nearly unchanged (see **Table 2** in the main manuscript and **Table S17**). For instance, the coefficient of youth age on the youth–caregiver internalizing discrepancy was 0.126 in the multiple imputation analysis and 0.128 in the complete-case model. More generally, key predictors – such as youth sex, caregiver depressive symptoms, and family conflict – retained the same signs, similar magnitudes, and statistical significance across all nine models.

Finally, the multiple-imputation-based age-by-reporter trajectories closely reproduced the complete-case curves, with visually negligible differences on the 0–12 symptom scales (see **Figure 2** in the main manuscript and **Figure S4**). he MI-based trajectories preserve the same developmental patterns across domains, indicating that the age-by-reporter differences reported in the main analyses are not driven by selective missingness.

**Table S16.** Multiple imputation results for mean-level differences

|  | Differences in symptoms, by reporters | | |  |
| --- | --- | --- | --- | --- |
|  | Youth - Caregiver | Youth - Teacher | Caregiver – Teacher |  |
|  | (1) | (2) | (3) |  |
|  |  |  |  |  |
| Internalizing symptoms | 0.411 [0.167] *** | 0.375 [0.137] *** | -0.072 [-0.029] * |  |
| Inattention symptoms | 1.205 [0.400] *** | 0.882 [0.266] *** | -0.336 [-0.114] *** |  |
| Externalizing symptoms | 0.410 [0.181] *** | 0.911 [0.400] *** | 0.365 [0.169] *** |  |
|  |  |  |  |  |
|  |  |  |  |  |
| **Notes:** Results based on the BPM-Y, BPM-T, and CBCL, which are available for all participants from baseline to the three-year follow-up for the caregiver and teacher, and from the one-year follow-up to the three-year follow-up for the youth. We focused our analysis on data from one- to three-year follow-up. Columns (1) to (3) show the average difference between reporters and the statistical significance according to t-tests. Standardized differences in brackets. All discrepancies are based on 32,457 observations. *** p<0.001, ** p<0.01, * p<0.05, t <0.10. | | | |  |
|  |  |  |  |  |
|  |  |  |  |  |
|  |  |  |  |  |
|  |  |  |  |  |
|  |  |  |  |  |

**Table S17.** Multiple imputation results for predictors of mean-level discrepancies

|  | Youth - Caregiver | | |  | Youth - Teacher | | |  | Caregiver - Teacher | | |  |
| --- | --- | --- | --- | --- | --- | --- | --- | --- | --- | --- | --- | --- |
|  | INT | INA | EXT |  | INT | INA | EXT |  | INT | INA | EXT |  |
|  | (1) | (2) | (3) |  | (4) | (5) | (6) |  | (7) | (8) | (9) |  |
|  |  |  |  |  |  |  |  |  |  |  |  |  |
| Youth's characteristics | | | | | | | | | | | |  |
| Age (in years) | 0.126*** | 0.229*** | 0.101*** |  | 0.231*** | 0.306*** | 0.227*** |  | 0.098*** | 0.068t | 0.125*** |  |
|  | (0.012) | (0.016) | (0.011) |  | (0.022) | (0.037) | (0.023) |  | (0.025) | (0.036) | (0.024) |  |
| Sex at birth  (girl = 1) | 0.516*** | 0.884*** | 0.382*** |  | 0.468*** | 1.484*** | 0.506*** |  | 0.054 | 0.720*** | 0.238*** |  |
|  | (0.034) | (0.043) | (0.032) |  | (0.060) | (0.054) | (0.038) |  | (0.043) | (0.063) | (0.036) |  |
| Race  (non-white = 1) | 0.137** | 0.138* | 0.113* |  | -0.104t | -0.050 | -0.079 |  | -0.188* | -0.186*** | -0.135** |  |
|  | (0.051) | (0.064) | (0.048) |  | (0.062) | (0.105) | (0.066) |  | (0.075) | (0.056) | (0.048) |  |
| Pubertal  Development | 0.088*** | -0.009 | 0.008 |  | 0.062** | 0.005 | 0.017 |  | -0.044 | -0.000 | -0.022 |  |
|  | (0.017) | (0.021) | (0.016) |  | (0.023) | (0.042) | (0.019) |  | (0.029) | (0.029) | (0.027) |  |
| Immigrant (born  outside US = 1) | 0.141 | -0.316* | -0.136 |  | -0.065 | -0.153 | 0.085 |  | -0.040 | 0.361** | 0.342* |  |
|  | (0.108) | (0.141) | (0.104) |  | (0.199) | (0.190) | (0.138) |  | (0.112) | (0.134) | (0.144) |  |
| Caregiver's characteristics | | | | | | | | | | | |  |
| Age (in years) | -0.003 | -0.011** | -0.006* |  | -0.012** | -0.011* | -0.001 |  | -0.010* | 0.002 | 0.001 |  |
|  | (0.003) | (0.004) | (0.003) |  | (0.004) | (0.005) | (0.003) |  | (0.004) | (0.004) | (0.003) |  |
| Sex at birth  (female = 1) | -0.258*** | -0.191** | -0.205*** |  | -0.087 | -0.211* | -0.095t |  | 0.183** | -0.010 | 0.135 |  |
|  | (0.058) | (0.073) | (0.055) |  | (0.095) | (0.107) | (0.057) |  | (0.060) | (0.081) | (0.086) |  |
| Race  (non-white = 1) | 0.178** | -0.037 | 0.190*** |  | 0.045 | -0.528*** | -0.488*** |  | -0.313*** | -0.566*** | -0.745*** |  |
|  | (0.056) | (0.070) | (0.053) |  | (0.105) | (0.089) | (0.081) |  | (0.062) | (0.086) | (0.065) |  |
| Immigrant (born  outside US = 1) | 0.149** | 0.088 | 0.031 |  | 0.286** | 0.213* | 0.324*** |  | 0.141* | 0.315** | 0.276*** |  |
|  | (0.053) | (0.070) | (0.051) |  | (0.092) | (0.083) | (0.052) |  | (0.068) | (0.096) | (0.055) |  |
| Depressive  Symptoms | -0.375*** | -0.398*** | -0.330*** |  | 0.045t | 0.112*** | 0.039* |  | 0.440*** | 0.537*** | 0.355*** |  |
|  | (0.019) | (0.024) | (0.018) |  | (0.023) | (0.029) | (0.017) |  | (0.019) | (0.029) | (0.019) |  |
|  |  |  |  |  |  |  |  |  |  |  |  |  |
|  |  |  |  |  |  |  |  |  |  |  |  |  |
| (Continues in next page) | | | | | | | | | | | |  |
| Education level |  |  |  |  |  |  |  |  |  |  |  |  |
| Some College | -0.046 | 0.044 | -0.001 |  | 0.209* | 0.455*** | 0.195* |  | 0.263** | 0.577*** | 0.257** |  |
|  | (0.065) | (0.082) | (0.062) |  | (0.098) | (0.114) | (0.091) |  | (0.095) | (0.115) | (0.094) |  |
| Associate  Degree | -0.113 | -0.075 | -0.121t |  | 0.180 | 0.411*** | 0.243** |  | 0.193* | 0.505*** | 0.302*** |  |
|  | (0.070) | (0.088) | (0.067) |  | (0.110) | (0.113) | (0.081) |  | (0.089) | (0.142) | (0.083) |  |
| College | -0.358*** | -0.269*** | -0.158** |  | 0.348*** | 0.621*** | 0.424*** |  | 0.681*** | 0.987*** | 0.577*** |  |
|  | (0.065) | (0.081) | (0.060) |  | (0.072) | (0.111) | (0.073) |  | (0.079) | (0.101) | (0.079) |  |
| Masters or more | -0.426*** | -0.264** | -0.133* |  | 0.380*** | 0.876*** | 0.361*** |  | 0.715*** | 1.163*** | 0.491*** |  |
|  | (0.068) | (0.082) | (0.062) |  | (0.094) | (0.124) | (0.075) |  | (0.093) | (0.126) | (0.083) |  |
| Social environments | | | | | | | | | | | |  |
| Parental warmth | -0.120*** | -0.126*** | -0.023 |  | -0.029 | -0.103** | -0.043t |  | 0.097** | 0.006 | -0.056** |  |
|  | (0.018) | (0.023) | (0.018) |  | (0.031) | (0.032) | (0.026) |  | (0.032) | (0.024) | (0.022) |  |
| Prosocial school  Environment | -0.061*** | -0.168*** | -0.123*** |  | -0.111*** | -0.177*** | -0.019 |  | -0.017 | 0.021 | 0.114*** |  |
|  | (0.017) | (0.022) | (0.017) |  | (0.028) | (0.042) | (0.024) |  | (0.027) | (0.031) | (0.025) |  |
| Family conflict | 0.147*** | 0.212*** | 0.200*** |  | 0.168*** | 0.233*** | 0.283*** |  | -0.077*** | -0.026 | 0.045* |  |
|  | (0.017) | (0.021) | (0.017) |  | (0.029) | (0.032) | (0.022) |  | (0.022) | (0.030) | (0.022) |  |
| Neighborhood  Deprivation | 0.041 | 0.023 | 0.006 |  | -0.012 | 0.031 | -0.011 |  | -0.029 | 0.044 | -0.021 |  |
|  | (0.026) | (0.027) | (0.023) |  | (0.023) | (0.034) | (0.028) |  | (0.029) | (0.033) | (0.022) |  |
|  |  |  |  |  |  |  |  |  |  |  |  |  |
|  |  |  |  |  |  |  |  |  |  |  |  |  |
| Ave. outcome | 0.404 | 1.197 | 0.406 |  | 0.305 | 0.842 | 0.877 |  | -0.0252 | -0.260 | 0.403 |  |
| N research sites | 21 | 21 | 21 |  | 21 | 21 | 21 |  | 21 | 21 | 21 |  |
| N families | 9,554 | 9,554 | 9,554 |  | 9,554 | 9,554 | 9,554 |  | 9,554 | 9,554 | 9,554 |  |
| N person-wave dyads | 32,457 | 32,457 | 32,457 |  | 32,457 | 32,457 | 32,457 |  | 32,457 | 32,457 | 32,457 |  |
|  |  |  |  |  |  |  |  |  |  |  |  |  |
|  |  |  |  |  |  |  |  |  |  |  |  |  |
| INT: Internalizing; INA: Inattention; EXT: Externalizing; Ave.: Average; N: Number | | | | | | | | | | | |  |
| **Notes**: Each column represents an independent model. All models were fitted using multilevel mixed-effects linear regression and the estimations were performed by maximum likelihood using Stata 19 and the command MIXED. *** p<0.001, ** p<0.01, * p<0.05, t <0.10. | | | | | | | | | | | |  |
|  |  |  |  |  |  |  |  |  |  |  |  |  |

**Table S18.** Multiple imputation results for predictors of mean-level discrepancies at the domains x reporters-level data

|  | Mental health domains | | | |  |
| --- | --- | --- | --- | --- | --- |
|  | INT | INA | EXT | All |  |
|  | (1) | (2) | (3) | (4) |  |
|  |  |  |  |  |  |
| Reporters | | | | |  |
| Caregivers | 0.859*** | 1.867*** | 0.506** | 1.078*** |  |
|  | (0.180) | (0.210) | (0.165) | (0.112) |  |
| Teachers | 0.723** | 2.008*** | -0.575* | 0.719*** |  |
|  | (0.230) | (0.253) | (0.226) | (0.151) |  |
| Caregivers x youth's age | -0.106*** | -0.245*** | -0.084*** | -0.145*** |  |
|  | (0.015) | (0.018) | (0.014) | (0.009) |  |
| Teachers x youth's age | -0.065*** | -0.262*** | 0.025 | -0.101*** |  |
|  | (0.019) | (0.021) | (0.019) | (0.013) |  |
| Youth's characteristics | | | | |  |
| Age (in years) | 0.126*** | 0.196*** | 0.072*** | 0.132*** |  |
|  | (0.011) | (0.013) | (0.010) | (0.007) |  |
| Sex at birth (girl = 1) | 0.240*** | -0.510*** | -0.181*** | -0.148*** |  |
|  | (0.020) | (0.028) | (0.019) | (0.016) |  |
| Race (non-white = 1) | 0.093** | 0.229*** | 0.150*** | 0.154*** |  |
|  | (0.030) | (0.042) | (0.028) | (0.026) |  |
| Pubertal development | 0.082*** | 0.072*** | 0.058*** | 0.064*** |  |
|  | (0.011) | (0.013) | (0.010) | (0.007) |  |
| Immigrant (born outside US = 1) | 0.026 | 0.007 | 0.015 | 0.018 |  |
|  | (0.066) | (0.086) | (0.059) | (0.053) |  |
| Caregiver's characteristics | | | | |  |
| Age (in years) | 0.002 | 0.004 | -0.002 | 0.001 |  |
|  | (0.002) | (0.002) | (0.002) | (0.001) |  |
| Sex at birth (female = 1) | -0.015 | 0.049 | 0.079* | 0.039 |  |
|  | (0.038) | (0.045) | (0.033) | (0.031) |  |
| Race (non-white = 1) | -0.205*** | -0.137** | -0.044 | -0.124*** |  |
|  | (0.034) | (0.043) | (0.030) | (0.027) |  |
| Immigrant (born outside US = 1) | -0.046 | -0.198*** | -0.122*** | -0.124*** |  |
|  | (0.031) | (0.045) | (0.032) | (0.028) |  |
| Depressive symptoms | 0.370*** | 0.429*** | 0.297*** | 0.364*** |  |
|  | (0.011) | (0.015) | (0.011) | (0.009) |  |
|  |  |  |  |  |  |
|  |  |  |  |  |  |
|  |  |  |  |  |  |
| (Continues in the next page) | | | | |  |
|  |  |  |  |  |  |
|  |  |  |  |  |  |
| Education level |  |  |  |  |  |
| Some College | 0.030 | 0.032 | -0.042 | -0.003 |  |
|  | (0.041) | (0.052) | (0.035) | (0.032) |  |
| Associate Degree | 0.030 | -0.018 | -0.029 | -0.012 |  |
|  | (0.041) | (0.055) | (0.041) | (0.035) |  |
| College | -0.017 | -0.156** | -0.174*** | -0.114*** |  |
|  | (0.040) | (0.050) | (0.034) | (0.032) |  |
| Masters or more | 0.016 | -0.205*** | -0.181*** | -0.130*** |  |
|  | (0.041) | (0.053) | (0.037) | (0.034) |  |
| Social environments | | | | |  |
| Parental warmth | -0.046*** | -0.099*** | -0.097*** | -0.082*** |  |
|  | (0.012) | (0.014) | (0.010) | (0.009) |  |
| Prosocial school environment | -0.120*** | -0.143*** | -0.106*** | -0.116*** |  |
|  | (0.012) | (0.013) | (0.009) | (0.008) |  |
| Family conflict | 0.119*** | 0.208*** | 0.182*** | 0.160*** |  |
|  | (0.011) | (0.013) | (0.010) | (0.008) |  |
| Neighborhood deprivation | 0.024t | 0.045* | 0.043** | 0.038** |  |
|  | (0.013) | (0.020) | (0.014) | (0.012) |  |
|  |  |  |  |  |  |
|  |  |  |  |  |  |
| Average outcome | 1.613 | 2.662 | 1.467 | 1.909 |  |
| Number of observations | 97,371 | 97,371 | 97,371 | 292,113 |  |
|  |  |  |  |  |  |
|  |  |  |  |  |  |
| INT: Internalizing; INA: Inattention; EXT: Externalizing; Ave.: Average; N: Number | | | | |  |
| **Notes**: Each column represents an independent model. All models were fitted using multilevel mixed-effects linear regression and the estimations were performed by maximum likelihood using Stata 19 and the command MIXED. *** p<0.001, ** p<0.01, * p<0.05, t <0.10. | | | | |  |
|  |  |  |  |  |  |
|  |  |  |  |  |  |

**Figure S4.** Multiple imputation for mental health symptoms over youth age


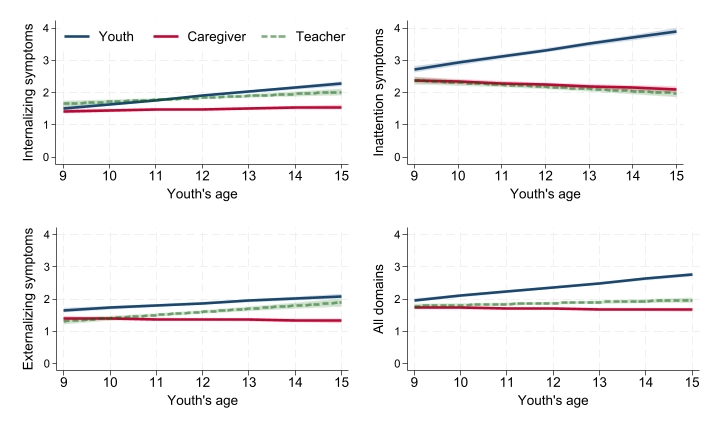


# **Appendix S7**: Correction of multiple testing

To address concerns about inflated Type I error due to the large number of tests reported in **Tables 2–3** in the main manuscript, we implemented formal corrections for multiple comparisons for (a) the reporter contrasts in **Table 2** and (b) the predictor coefficients in **Table 3**. We applied two procedures: (i) a Benjamini–Hochberg false discovery rate (FDR) procedure at q = .05 and (ii) a Bonferroni family-wise error rate correction at α = .05. For the mean-level contrasts (Table 2), corrections were applied separately within each reporter-pair family of tests (youth–caregiver, youth–teacher, caregiver–teacher). For the predictors models (Table 3), corrections were applied within each predictor across the nine discrepancy outcomes (three domains x three reporter-pair comparisons). We then annotated **Table S19** and **Table S20** to indicate which findings remain statistically significant after each procedure (BH = survives Benjamini–Hochberg; B = survives Bonferroni).

Results were robust to both correction strategies. For mean-level discrepancies (**Table S19**), all contrasts that were statistically significant in the original analyses remained significant after both FDR and Bonferroni correction. For predictors of discrepancies (**Table S20**), the main patterns of association were largely unchanged: the strongest and most consistent predictors (e.g., youth age and sex, caregiver depressive symptoms, family conflict, and prosocial school environment) generally remained significant after FDR correction, and many of the larger effects also survived Bonferroni correction. Together, these results suggest that our primary conclusions are not driven by Type I error associated with multiple testing.

**Table S19.** Multiple testing correction for mean-level discrepancies

|  | Differences in symptoms, by reporters | | |  |
| --- | --- | --- | --- | --- |
|  | Youth - Caregiver | Youth - Teacher | Caregiver - Teacher |  |
|  | (1) | (2) | (3) |  |
|  |  |  |  |  |
| Internalizing symptoms | 0.405 [0.165] ^BH, B^ | 0.304 [0.111] ^BH, B^ | -0.027 [-0.011] |  |
| Inattention symptoms | 1.198 [0.398] ^BH, B^ | 0.842 [0.255] ^BH, B^ | -0.259 [-0.088] ^BH, B^ |  |
| Externalizing symptoms | 0.406 [0.179] ^BH, B^ | 0.877 [0.385] ^BH, B^ | 0.404 [0.187] ^BH, B^ |  |
|  |  |  |  |  |
|  |  |  |  |  |
| BH: Survives Benjamini-Hochberg; B: Survives Bonferroni correction (p<0.05) | | | |  |
| **Notes:** Results based on the BPM-Y, BPM-T, and CBCL, which are available for all participants from baseline to the three-year follow-up for the caregiver and teacher, and from the one-year follow-up to the three-year follow-up for the youth. We focused our analysis on data from one- to three-year follow-up. Columns (1) to (3) show the average difference between reporters and the statistical significance according to a paired t-tests. Standardized differences in brackets. Differences in Column (1) are based on around 30,000 observations, and differences in reported in Columns (2) and (3) are based on around 12,000 observations *** p<0.001, ** p<0.01, * p<0.05, t <0.10. | | | |  |
|  |  |  |  |  |
|  |  |  |  |  |
|  |  |  |  |  |
|  |  |  |  |  |
|  |  |  |  |  |

**Table S20.** Multiple testing correction for predictors of mean-level discrepancies

|  | Youth - Caregiver | | |  | Youth - Teacher | | |  | Caregiver - Teacher | | |  |
| --- | --- | --- | --- | --- | --- | --- | --- | --- | --- | --- | --- | --- |
|  | INT | INA | EXT |  | INT | INA | EXT |  | INT | INA | EXT |  |
|  | (1) | (2) | (3) |  | (4) | (5) | (6) |  | (7) | (8) | (9) |  |
|  |  |  |  |  |  |  |  |  |  |  |  |  |
| Youth's characteristics | | | | | | | | | | | |  |
| Age (in years) | 0.132^BH, B^ | 0.235^BH, B^ | 0.106^BH, B^ |  | 0.234^BH, B^ | 0.309^BH, B^ | 0.223^BH, B^ |  | 0.082^BH, B^ | 0.070^BH, B^ | 0.119^BH, B^ |  |
|  | (0.012) | (0.015) | (0.011) |  | (0.026) | (0.030) | (0.020) |  | (0.023) | (0.026) | (0.018) |  |
| Sex at birth  (girl = 1) | 0.518^BH, B^ | 0.875^BH, B^ | 0.378^BH, B^ |  | 0.480^BH, B^ | 1.461^BH, B^ | 0.555^BH, B^ |  | 0.098 | 0.707^BH, B^ | 0.260^BH, B^ |  |
|  | (0.036) | (0.046) | (0.034) |  | (0.057) | (0.069) | (0.048) |  | (0.051) | (0.060) | (0.044) |  |
| Race  (non-white = 1) | 0.136^BH^ | 0.154^BH^ | 0.106 |  | -0.048 | -0.017 | -0.156^BH^ |  | -0.242^BH, B^ | -0.200^BH^ | -0.212^BH, B^ |  |
|  | (0.054) | (0.069) | (0.051) |  | (0.080) | (0.098) | (0.068) |  | (0.071) | (0.084) | (0.062) |  |
| Pubertal  Development | 0.088^BH, B^ | -0.030 | -0.011 |  | 0.064^BH^ | 0.003 | 0.009 |  | -0.027 | 0.018 | -0.029 |  |
|  | (0.018) | (0.022) | (0.017) |  | (0.029) | (0.035) | (0.024) |  | (0.026) | (0.030) | (0.022) |  |
| Immigrant (born  outside US = 1) | 0.146 | -0.361 | -0.156 |  | -0.034 | -0.243 | 0.242 |  | 0.032 | 0.303 | 0.398 |  |
|  | (0.115) | (0.148) | (0.111) |  | (0.188) | (0.233) | (0.161) |  | (0.170) | (0.200) | (0.147) |  |
| Caregiver's characteristics | | | | | | | | | | | |  |
| Age (in years) | -0.000 | -0.008 | -0.005 |  | -0.010 | -0.009 | -0.001 |  | -0.009 | 0.000 | -0.000 |  |
|  | (0.003) | (0.004) | (0.003) |  | (0.005) | (0.006) | (0.004) |  | (0.004) | (0.005) | (0.004) |  |
| Sex at birth  (female = 1) | -0.296^BH, B^ | -0.201^BH^ | -0.223^BH, B^ |  | -0.099 | -0.220 | -0.077 |  | 0.236^BH, B^ | -0.031 | 0.147 |  |
|  | (0.061) | (0.078) | (0.059) |  | (0.095) | (0.116) | (0.080) |  | (0.085) | (0.100) | (0.074) |  |
| Race  (non-white = 1) | 0.153^BH^ | -0.056 | 0.180^BH, B^ |  | -0.090 | -0.556^BH, B^ | -0.438^BH, B^ |  | -0.241^BH, B^ | -0.581^BH, B^ | -0.630^BH, B^ |  |
|  | (0.059) | (0.075) | (0.056) |  | (0.097) | (0.119) | (0.082) |  | (0.086) | (0.101) | (0.075) |  |
| Immigrant (born  outside US = 1) | 0.143^BH^ | 0.078 | 0.025 |  | 0.285^BH, B^ | 0.255 | 0.299 ^BH, B^ |  | 0.144 | 0.360^BH, B^ | 0.266^BH, B^ |  |
|  | (0.057) | (0.073) | (0.054) |  | (0.091) | (0.111) | (0.076) |  | (0.081) | (0.095) | (0.070) |  |
| Depressive  Symptoms | -0.378^BH, B^ | -0.394^BH, B^ | -0.333^BH, B^ |  | 0.036 | 0.099^BH^ | 0.031 |  | 0.432^BH, B^ | 0.514^BH, B^ | 0.333^BH, B^ |  |
|  | (0.020) | (0.026) | (0.019) |  | (0.031) | (0.037) | (0.026) |  | (0.027) | (0.032) | (0.024) |  |
|  |  |  |  |  |  |  |  |  |  |  |  |  |
|  |  |  |  |  |  |  |  |  |  |  |  |  |
| (Continues in the next page) | | | | | | | | | | | |  |
| Education level |  |  |  |  |  |  |  |  |  |  |  |  |
| Some College | -0.047 | 0.035 | -0.036 |  | 0.259^BH^ | 0.439^BH, B^ | 0.194 |  | 0.355^BH, B^ | 0.499^BH, B^ | 0.277^BH, B^ |  |
|  | (0.070) | (0.090) | (0.067) |  | (0.115) | (0.140) | (0.097) |  | (0.102) | (0.120) | (0.089) |  |
| Associate  Degree | -0.100 | -0.082 | -0.126 |  | 0.241 | 0.401^BH^ | 0.140 |  | 0.313^BH, B^ | 0.525^BH, B^ | 0.258^BH^ |  |
|  | (0.074) | (0.096) | (0.071) |  | (0.120) | (0.147) | (0.101) |  | (0.107) | (0.126) | (0.093) |  |
| College | -0.354^BH, B^ | -0.284^BH, B^ | -0.180^BH, B^ |  | 0.387^BH, B^ | 0.662^BH, B^ | 0.427^BH, B^ |  | 0.729^BH, B^ | 0.936^BH, B^ | 0.579^BH, B^ |  |
|  | (0.068) | (0.087) | (0.065) |  | (0.108) | (0.132) | (0.091) |  | (0.096) | (0.113) | (0.084) |  |
| Masters or  More | -0.425^BH, B^ | -0.278^BH, B^ | -0.143 |  | 0.442^BH, B^ | 0.932^BH, B^ | 0.356^BH, B^ |  | 0.835^BH, B^ | 1.164^BH, B^ | 0.498^BH, B^ |  |
|  | (0.071) | (0.091) | (0.068) |  | (0.112) | (0.137) | (0.095) |  | (0.100) | (0.117) | (0.087) |  |
| Social environments | | | | | | | | | | | |  |
| Parental warmth | -0.131^BH, B^ | -0.118^BH, B^ | -0.021 |  | -0.032 | -0.108^BH, B^ | -0.043 |  | 0.097^BH, B^ | 0.012 | -0.066^BH, B^ |  |
|  | (0.019) | (0.024) | (0.018) |  | (0.032) | (0.038) | (0.026) |  | (0.028) | (0.032) | (0.024) |  |
| Prosocial school  Environment | -0.058^BH, B^ | -0.176^BH, B^ | -0.122^BH, B^ |  | -0.117^BH, B^ | -0.180^BH, B^ | -0.038 |  | -0.015 | 0.009 | 0.102^BH, B^ |  |
|  | (0.019) | (0.023) | (0.018) |  | (0.031) | (0.037) | (0.026) |  | (0.027) | (0.032) | (0.023) |  |
| Family conflict | 0.149^BH, B^ | 0.205^BH, B^ | 0.220^BH, B^ |  | 0.124^BH, B^ | 0.208^BH, B^ | 0.255^BH, B^ |  | -0.085^BH, B^ | -0.024 | 0.006 |  |
|  | (0.018) | (0.023) | (0.017) |  | (0.030) | (0.036) | (0.025) |  | (0.027) | (0.031) | (0.023) |  |
| Neighborhood  Deprivation | 0.047 | 0.031 | 0.015 |  | -0.006 | 0.040 | -0.022 |  | -0.018 | 0.040 | -0.009 |  |
|  | (0.027) | (0.031) | (0.024) |  | (0.039) | (0.046) | (0.032) |  | (0.033) | (0.038) | (0.029) |  |
|  |  |  |  |  |  |  |  |  |  |  |  |  |
|  |  |  |  |  |  |  |  |  |  |  |  |  |
| Ave. outcome | 0.393 | 1.199 | 0.400 |  | 0.300 | 0.853 | 0.888 |  | -0.0228 | -0.255 | 0.417 |  |
| N research sites | 21 | 21 | 21 |  | 21 | 21 | 21 |  | 21 | 21 | 21 |  |
| N families | 8,817 | 8,783 | 8,818 |  | 6,266 | 6,155 | 6,262 |  | 6,422 | 6,457 | 6,450 |  |
| N pw dyads | 28,064 | 27,135 | 27,878 |  | 11,006 | 10,682 | 10,992 |  | 11,467 | 11,518 | 11,518 |  |
|  |  |  |  |  |  |  |  |  |  |  |  |  |
|  |  |  |  |  |  |  |  |  |  |  |  |  |
| BH: Survives Benjamini-Hochberg; B: Survives Bonferroni correction (p<0.05)  INT: Internalizing; INA: Inattention; EXT: Externalizing; Ave.: Average; N: Number; pw: person-wave | | | | | | | | | | | |  |
| **Notes**: Each column represents an independent model. All models were fitted using multilevel mixed-effects linear regression and the estimations were performed by maximum likelihood using Stata 19 and the command MIXED. *** p<0.001, ** p<0.01, * p<0.05, t <0.10. | | | | | | | | | | | |  |
|  |  |  |  |  |  |  |  |  |  |  |  |  |

# **Appendix S8**: Alternative specifications for the predictors model

We further examined the associations between our predicting variables and reporters’ discrepancies by re-estimating the predictor models using alternative specifications using (a) standardized values for the discrepancies to report standardized coefficient (i.e., we z-standardized discrepancy outcomes – mean = 0, SD = 1 – so coefficients can be interpreted in SD units), (b) time-varying caregiver age and depressive symptoms as well as social-context variables (instead of baseline-only covariates**)^[[1]](#footnote-1)^**, (c) parental reports of pubertal development (instead of youth reports**)**, and (d) data from each of the follow-ups to replicate the main analysis separately for 1-year, 2-year, and 3-year follow-ups.

The standardized coefficients models (see **Table S21**) closely mirrored the main predictor results, indicating that the substantive conclusions are not sensitive to the scale of the discrepancy outcomes. Youth age was positively associated with larger discrepancies across all reporter dyads and domains (βs ≈ 0.02–0.10 SD per year; all p’s < .01), with the largest standardized associations generally observed for youth–teacher discrepancies (e.g., β = 0.085–0.098 across domains). Female youth showed higher youth–adult discrepancies across domains (β = 0.167–0.442; all p’s < .001), while caregiver–teacher discrepancies were also higher for girls in inattention and externalizing (β = 0.240 and 0.121, respectively). Caregiver depressive symptoms remained one of the most consistent predictors of discrepancies (youth–caregiver: β = −0.131 to −0.154; caregiver–teacher: β = 0.154–0.175; p’s < .001), consistent with the sign pattern in the main models (i.e., higher caregiver depressive symptoms are associated with caregivers reporting more symptoms relative to youth, and fewer symptoms relative to teachers). Family conflict continued to predict larger youth–adult discrepancies across all domains (β = 0.045–0.112; p’s < .001), whereas parental warmth and prosocial school environment were generally associated with smaller discrepancies, particularly for youth–caregiver and youth–teacher dyads (e.g., prosocial school environment: β = −0.024 to −0.058 for youth–caregiver and β = −0.043 to −0.055 for youth–teacher; p’s < .01).

Specifications that replaced baseline covariates with their time-varying values yielded results that were broadly consistent with the main predictor models (Table 3), with similar directions and patterns of statistical significance across most youth and caregiver characteristics (**Table S22**). Youth age remained a robust predictor of discrepancies in all dyad-by-domain models, indicating that reporter differences systematically shift with age, although the magnitude of coefficients decreased as part of the variation in other significant covariates is associated with age (e.g., parental warmth). The most notable changes relative to the baseline-covariate specification appear for the socio-contextual measures: when modeled as time-varying, caregiver warmth, prosocial school environment, and family conflict showed substantially larger associations with discrepancies than in Table 3, while preserving the same direction of effects (warmth and prosocial school environment predicting smaller/more negative discrepancies; family conflict predicting larger/more positive discrepancies). Caregiver depressive symptoms remained statistically significant in most models but with attenuated magnitudes relative to the baseline specification (e.g., youth–caregiver discrepancies: −0.138 to −0.115 in Table S22 vs. −0.378 to −0.333 in Table 3), consistent with contemporaneous symptom reports partly accounting for stable between-family differences captured by baseline measures. Neighborhood deprivation continued to show small and generally non-significant associations. We interpret these specifications as robustness checks, given uneven measurement schedules across constructs and waves. Overall, allowing key caregiver and contextual variables to vary over time does not materially change the substantive conclusions, while suggesting that contemporaneous family and school environments are more strongly linked to discrepancies than baseline levels.

Following previous studies documenting differences between caregivers’ and youths’ reports of pubertal development in the ABCD data (Beltz et al., 2025; Cheng et al., 2021), we re-estimated the predictors models using caregivers’ reports of pubertal development in place of youths’ self-reports (see **Table S23**). Focusing on pubertal development, the main pattern emphasized in the manuscript remains: more advanced pubertal development continues to be associated with larger youth–caregiver discrepancies in internalizing symptoms (β = 0.049, p < .05), consistent with the corresponding result in the baseline specification (Table 3). For the remaining dyad-by-domain outcomes, associations between pubertal development and discrepancies are smaller and less consistent across models; accordingly, we do not place interpretive weight on those coefficients in the main text. Importantly, replacing youth-reported with caregiver-reported pubertal development does not materially change the estimated associations for other predictors (e.g., youth age and sex, caregiver depressive symptoms, caregiver education, family conflict, and prosocial school environment), which remain very similar in magnitude and statistical significance to Table 3.

Lastly, we replicated the predictors models separately using data from each follow-up wave (1-year, 2-year, and 3-year) to assess whether baseline characteristics show similar associations with discrepancies at specific time points (**Table S24**, **Table S25**, and **Table S26**). Across waves, most patterns remain, particularly for the larger and most consistently estimated predictors. Youth sex (girl = 1) is positively associated with discrepancies for most reporter pairs and domains at each wave, with especially robust associations for inattention and externalizing discrepancies. Caregiver depressive symptoms also show a stable pattern across waves: higher depressive symptoms predict smaller youth–caregiver discrepancies (i.e., more similar youth and caregiver reports) and larger caregiver–teacher discrepancies across domains, while associations with youth–teacher discrepancies are weaker and less consistently significant. Family conflict generally predicts larger discrepancies for youth–caregiver and youth–teacher comparisons across waves, whereas prosocial school environment and parental warmth tend to predict smaller discrepancies in the youth–caregiver models (and in some cases youth–teacher), though significance varies by wave and outcome. In contrast, the association with youth age is less stable across the single-wave models, particularly for youth–caregiver internalizing at the 1-year follow-up (β = −0.003; **Table S24**), but the association becomes stronger by the 3-year follow-up (β = 0.188; **Table S26**). Overall, these wave-specific replications support the main conclusions while also highlighting that some smaller effects vary by follow-up wave and are estimated less precisely in models involving teacher reports likely due to smaller analytic samples.

**Table S21.** Predictors of mean-level discrepancies, standardized coefficients

|  | Youth – Caregiver | | |  | Youth – Teacher | | |  | Caregiver - Teacher | | |  |
| --- | --- | --- | --- | --- | --- | --- | --- | --- | --- | --- | --- | --- |
|  | INT | INA | EXT |  | INT | INA | EXT |  | INT | INA | EXT |  |
|  | (1) | (2) | (3) |  | (4) | (5) | (6) |  | (7) | (8) | (9) |  |
|  |  |  |  |  |  |  |  |  |  |  |  |  |
| Youth's characteristics | | | | | | | | | | | |  |
| Age (in years) | 0.054*** | 0.078*** | 0.047*** |  | 0.085*** | 0.093*** | 0.098*** |  | 0.032*** | 0.024** | 0.055*** |  |
|  | (0.005) | (0.005) | (0.005) |  | (0.009) | (0.009) | (0.009) |  | (0.009) | (0.009) | (0.009) |  |
| Sex at birth  (girl = 1) | 0.211*** | 0.291*** | 0.167*** |  | 0.175*** | 0.442*** | 0.243*** |  | 0.039t | 0.240*** | 0.121*** |  |
|  | (0.015) | (0.015) | (0.015) |  | (0.021) | (0.021) | (0.021) |  | (0.020) | (0.020) | (0.020) |  |
| Race  (non-white = 1) | 0.056* | 0.051* | 0.047* |  | -0.018 | -0.005 | -0.069* |  | -0.096*** | -0.068* | -0.098*** |  |
|  | (0.022) | (0.023) | (0.023) |  | (0.029) | (0.030) | (0.030) |  | (0.028) | (0.028) | (0.029) |  |
| Pubertal  Development | 0.036*** | -0.010 | -0.005 |  | 0.023* | 0.001 | 0.004 |  | -0.011 | 0.006 | -0.014 |  |
|  | (0.007) | (0.007) | (0.007) |  | (0.011) | (0.011) | (0.011) |  | (0.010) | (0.010) | (0.010) |  |
| Immigrant (born  outside US = 1) | 0.060 | -0.120* | -0.069 |  | -0.012 | -0.073 | 0.106 |  | 0.013 | 0.103 | 0.184** |  |
|  | (0.047) | (0.049) | (0.049) |  | (0.069) | (0.071) | (0.071) |  | (0.067) | (0.068) | (0.068) |  |
| Caregiver's characteristics | | | | | | | | | | | |  |
| Age (in years) | -0.000 | -0.003t | -0.002 |  | -0.004* | -0.003 | -0.000 |  | -0.004* | 0.000 | -0.000 |  |
|  | (0.001) | (0.001) | (0.001) |  | (0.002) | (0.002) | (0.002) |  | (0.002) | (0.002) | (0.002) |  |
| Sex at birth  (female = 1) | -0.121*** | -0.067* | -0.098*** |  | -0.036 | -0.066t | -0.034 |  | 0.094** | -0.011 | 0.068* |  |
|  | (0.025) | (0.026) | (0.026) |  | (0.035) | (0.035) | (0.035) |  | (0.034) | (0.034) | (0.034) |  |
| Race  (non-white = 1) | 0.062** | -0.019 | 0.080** |  | -0.033 | -0.168*** | -0.192*** |  | -0.096** | -0.197*** | -0.292*** |  |
|  | (0.024) | (0.025) | (0.025) |  | (0.035) | (0.036) | (0.036) |  | (0.034) | (0.034) | (0.035) |  |
| Immigrant (born  outside US = 1) | 0.058* | 0.026 | 0.011 |  | 0.104** | 0.077* | 0.131*** |  | 0.057t | 0.122*** | 0.123*** |  |
|  | (0.023) | (0.024) | (0.024) |  | (0.033) | (0.034) | (0.034) |  | (0.032) | (0.032) | (0.033) |  |
| Depressive  Symptoms | -0.154*** | -0.131*** | -0.147*** |  | 0.013 | 0.030** | 0.013 |  | 0.172*** | 0.175*** | 0.154*** |  |
|  | (0.008) | (0.009) | (0.008) |  | (0.011) | (0.011) | (0.011) |  | (0.011) | (0.011) | (0.011) |  |
|  |  |  |  |  |  |  |  |  |  |  |  |  |
|  |  |  |  |  |  |  |  |  |  |  |  |  |
| (Continues in the next page) | | | | | | | | | | | |  |
|  |  |  |  |  |  |  |  |  |  |  |  |  |
| Education level |  |  |  |  |  |  |  |  |  |  |  |  |
| Some College | -0.019 | 0.012 | -0.016 |  | 0.095* | 0.133** | 0.085* |  | 0.141*** | 0.170*** | 0.129** |  |
|  | (0.029) | (0.030) | (0.030) |  | (0.042) | (0.042) | (0.043) |  | (0.041) | (0.041) | (0.041) |  |
| Associate  Degree | -0.041 | -0.027 | -0.056t |  | 0.088* | 0.121** | 0.061 |  | 0.124** | 0.178*** | 0.120** |  |
|  | (0.030) | (0.032) | (0.031) |  | (0.044) | (0.044) | (0.044) |  | (0.042) | (0.043) | (0.043) |  |
| College | -0.144*** | -0.094** | -0.080** |  | 0.141*** | 0.200*** | 0.187*** |  | 0.290*** | 0.318*** | 0.268*** |  |
|  | (0.028) | (0.029) | (0.029) |  | (0.039) | (0.040) | (0.040) |  | (0.038) | (0.038) | (0.039) |  |
| Masters or more | -0.173*** | -0.092** | -0.063* |  | 0.161*** | 0.282*** | 0.156*** |  | 0.332*** | 0.396*** | 0.231*** |  |
|  | (0.029) | (0.030) | (0.030) |  | (0.041) | (0.041) | (0.042) |  | (0.040) | (0.040) | (0.040) |  |
| Social environments | | | | | | | | | | | |  |
| Parental warmth | -0.053*** | -0.039*** | -0.009 |  | -0.012 | -0.033** | -0.019t |  | 0.039*** | 0.004 | -0.031** |  |
|  | (0.008) | (0.008) | (0.008) |  | (0.012) | (0.012) | (0.011) |  | (0.011) | (0.011) | (0.011) |  |
| Prosocial school  Environment | -0.024** | -0.058*** | -0.054*** |  | -0.043*** | -0.055*** | -0.017 |  | -0.006 | 0.003 | 0.047*** |  |
|  | (0.008) | (0.008) | (0.008) |  | (0.011) | (0.011) | (0.011) |  | (0.011) | (0.011) | (0.011) |  |
| Family conflict | 0.061*** | 0.068*** | 0.097*** |  | 0.045*** | 0.063*** | 0.112*** |  | -0.034** | -0.008 | 0.003 |  |
|  | (0.007) | (0.008) | (0.008) |  | (0.011) | (0.011) | (0.011) |  | (0.011) | (0.011) | (0.010) |  |
| Neighborhood  Deprivation | 0.019t | 0.010 | 0.006 |  | -0.002 | 0.012 | -0.009 |  | -0.007 | 0.014 | -0.004 |  |
|  | (0.011) | (0.010) | (0.010) |  | (0.014) | (0.014) | (0.014) |  | (0.013) | (0.013) | (0.013) |  |
|  |  |  |  |  |  |  |  |  |  |  |  |  |
|  |  |  |  |  |  |  |  |  |  |  |  |  |
| Ave. outcome | 0.000 | 0.000 | 0.000 |  | 0.000 | 0.000 | 0.000 |  | 0.000 | 0.000 | 0.000 |  |
| N research sites | 21 | 21 | 21 |  | 21 | 21 | 21 |  | 21 | 21 | 21 |  |
| N families | 8,817 | 8,783 | 8,818 |  | 6,266 | 6,155 | 6,262 |  | 6,422 | 6,457 | 6,450 |  |
| N person-wave dyads | 28,064 | 27,135 | 27,878 |  | 11,006 | 10,682 | 10,992 |  | 11,467 | 11,518 | 11,518 |  |
|  |  |  |  |  |  |  |  |  |  |  |  |  |
|  |  |  |  |  |  |  |  |  |  |  |  |  |
| INT: Internalizing; INA: Inattention; EXT: Externalizing; Ave.: Average; N: Number | | | | | | | | | | | |  |
| **Notes**: Each column represents an independent model. All models were fitted using multilevel mixed-effects linear regression and the estimations were performed by maximum likelihood using Stata 19 and the command MIXED. *** p<0.001, ** p<0.01, * p<0.05, t <0.10. | | | | | | | | | | | |  |
|  |  |  |  |  |  |  |  |  |  |  |  |  |

**Table S22.** Predictors of mean-level discrepancies, using time-varying covariates

|  | Youth - Caregiver | | |  | Youth – Teacher | | |  | Caregiver - Teacher | | |  |
| --- | --- | --- | --- | --- | --- | --- | --- | --- | --- | --- | --- | --- |
|  | INT | INA | EXT |  | INT | INA | EXT |  | INT | INA | EXT |  |
|  | (1) | (2) | (3) |  | (4) | (5) | (6) |  | (7) | (8) | (9) |  |
|  |  |  |  |  |  |  |  |  |  |  |  |  |
| Youth's characteristics | | | | | | | | | | | |  |
| Age (in years) | 0.066*** | 0.153*** | 0.051*** |  | 0.182*** | 0.230*** | 0.159*** |  | 0.096*** | 0.080** | 0.114*** |  |
|  | (0.013) | (0.016) | (0.012) |  | (0.027) | (0.031) | (0.021) |  | (0.023) | (0.027) | (0.019) |  |
| Sex at birth  (girl = 1) | 0.498*** | 0.833*** | 0.358*** |  | 0.476*** | 1.420*** | 0.518*** |  | 0.105* | 0.694*** | 0.251*** |  |
|  | (0.036) | (0.045) | (0.034) |  | (0.057) | (0.069) | (0.047) |  | (0.051) | (0.060) | (0.044) |  |
| Race  (non-white = 1) | 0.086 | 0.090 | 0.072 |  | -0.121 | -0.121 | -0.195** |  | -0.243*** | -0.220** | -0.195** |  |
|  | (0.053) | (0.068) | (0.051) |  | (0.080) | (0.098) | (0.068) |  | (0.071) | (0.084) | (0.063) |  |
| Pubertal  Development | 0.090*** | -0.028 | -0.005 |  | 0.053t | -0.008 | 0.006 |  | -0.045t | 0.003 | -0.034 |  |
|  | (0.017) | (0.022) | (0.017) |  | (0.029) | (0.036) | (0.024) |  | (0.026) | (0.030) | (0.022) |  |
| Immigrant (born  outside US = 1) | 0.165 | -0.272t | -0.175 |  | 0.015 | -0.175 | 0.290t |  | 0.058 | 0.343t | 0.425** |  |
|  | (0.113) | (0.147) | (0.110) |  | (0.188) | (0.233) | (0.160) |  | (0.170) | (0.200) | (0.147) |  |
| Caregiver's characteristics | | | | | | | | | | | |  |
| Age (in years) | 0.003 | -0.008* | -0.002 |  | -0.006 | -0.005 | 0.003 |  | -0.008t | 0.001 | -0.000 |  |
|  | (0.003) | (0.004) | (0.003) |  | (0.005) | (0.006) | (0.004) |  | (0.004) | (0.005) | (0.004) |  |
| Sex at birth  (female = 1) | -0.265*** | -0.191* | -0.183** |  | -0.117 | -0.244* | -0.071 |  | 0.217* | -0.052 | 0.105 |  |
|  | (0.060) | (0.077) | (0.058) |  | (0.094) | (0.115) | (0.079) |  | (0.084) | (0.100) | (0.074) |  |
| Race  (non-white = 1) | 0.121* | -0.093 | 0.144* |  | -0.019 | -0.480*** | -0.385*** |  | -0.179* | -0.529*** | -0.604*** |  |
|  | (0.059) | (0.075) | (0.056) |  | (0.098) | (0.119) | (0.082) |  | (0.087) | (0.102) | (0.076) |  |
| Immigrant (born  outside US = 1) | 0.121* | 0.048 | 0.027 |  | 0.299** | 0.299** | 0.277*** |  | 0.184* | 0.429*** | 0.255*** |  |
|  | (0.056) | (0.072) | (0.054) |  | (0.091) | (0.111) | (0.077) |  | (0.081) | (0.096) | (0.071) |  |
| Depressive  symptoms | -0.138*** | -0.139*** | -0.115*** |  | 0.005 | 0.022* | 0.010 |  | 0.151*** | 0.171*** | 0.110*** |  |
|  | (0.005) | (0.007) | (0.005) |  | (0.009) | (0.011) | (0.007) |  | (0.008) | (0.009) | (0.007) |  |
|  |  |  |  |  |  |  |  |  |  |  |  |  |
|  |  |  |  |  |  |  |  |  |  |  |  |  |
| (Continues in the next page) | | | | | | | | | | | |  |
|  |  |  |  |  |  |  |  |  |  |  |  |  |
| Education level |  |  |  |  |  |  |  |  |  |  |  |  |
| Some College | -0.027 | 0.022 | -0.017 |  | 0.225 t | 0.449** | 0.210* |  | 0.239* | 0.477*** | 0.256** |  |
|  | (0.070) | (0.091) | (0.068) |  | (0.116) | (0.142) | (0.098) |  | (0.103) | (0.122) | (0.090) |  |
| Associate  Degree | -0.073 | -0.073 | -0.065 |  | 0.206 t | 0.386** | 0.169t |  | 0.194t | 0.504*** | 0.248** |  |
|  | (0.074) | (0.096) | (0.072) |  | (0.121) | (0.148) | (0.102) |  | (0.108) | (0.128) | (0.095) |  |
| College | -0.312*** | -0.264** | -0.128 t |  | 0.361*** | 0.671*** | 0.461*** |  | 0.614*** | 0.924*** | 0.581*** |  |
|  | (0.068) | (0.087) | (0.065) |  | (0.109) | (0.133) | (0.092) |  | (0.097) | (0.115) | (0.085) |  |
| Masters or more | -0.400*** | -0.293** | -0.126t |  | 0.421*** | 0.920*** | 0.372*** |  | 0.742*** | 1.172*** | 0.512*** |  |
|  | (0.071) | (0.091) | (0.068) |  | (0.112) | (0.137) | (0.095) |  | (0.100) | (0.119) | (0.088) |  |
| Social environments | | | | | | | | | | | |  |
| Parental warmth | -0.208*** | -0.142*** | -0.049** |  | -0.096** | -0.133*** | -0.158*** |  | 0.129*** | -0.007 | -0.110*** |  |
|  | (0.016) | (0.020) | (0.015) |  | (0.031) | (0.037) | (0.025) |  | (0.027) | (0.032) | (0.023) |  |
| Prosocial school  Environment | -0.165*** | -0.316*** | -0.168*** |  | -0.242*** | -0.271*** | -0.102*** |  | -0.030 | 0.077* | 0.097*** |  |
|  | (0.015) | (0.019) | (0.014) |  | (0.030) | (0.036) | (0.024) |  | (0.026) | (0.030) | (0.022) |  |
| Family conflict | 0.328*** | 0.392*** | 0.382*** |  | 0.333*** | 0.422*** | 0.402*** |  | -0.041 | -0.003 | 0.026 |  |
|  | (0.016) | (0.020) | (0.015) |  | (0.030) | (0.036) | (0.024) |  | (0.027) | (0.031) | (0.022) |  |
| Neighborhood  deprivation | 0.044t | 0.029 | 0.015 |  | -0.009 | 0.033 | -0.025 |  | -0.020 | 0.034 | -0.015 |  |
|  | (0.026) | (0.031) | (0.024) |  | (0.038) | (0.045) | (0.032) |  | (0.033) | (0.038) | (0.029) |  |
|  |  |  |  |  |  |  |  |  |  |  |  |  |
|  |  |  |  |  |  |  |  |  |  |  |  |  |
| Ave. outcome | 0.388 | 1.201 | 0.400 |  | 0.321 | 0.874 | 0.905 |  | -0.00648 | -0.241 | 0.422 |  |
| N research sites | 21 | 21 | 21 |  | 21 | 21 | 21 |  | 21 | 21 | 21 |  |
| N families | 8,380 | 8,333 | 8,371 |  | 6,032 | 5,930 | 6,025 |  | 6,198 | 6,231 | 6,225 |  |
| N person-wave dyads | 26,862 | 25,980 | 26,681 |  | 10,629 | 10,317 | 10,607 |  | 11,103 | 11,148 | 11,147 |  |
|  |  |  |  |  |  |  |  |  |  |  |  |  |
|  |  |  |  |  |  |  |  |  |  |  |  |  |
| INT: Internalizing; INA: Inattention; EXT: Externalizing; Ave.: Average; N: Number | | | | | | | | | | | |  |
| **Notes**: Each column represents an independent model. All models were fitted using multilevel mixed-effects linear regression and the estimations were performed by maximum likelihood using Stata 19 and the command MIXED. *** p<0.001, ** p<0.01, * p<0.05, t <0.10. | | | | | | | | | | | |  |
|  |  |  |  |  |  |  |  |  |  |  |  |  |

**Table S23.** Predictors of mean-level discrepancies, using parental reports of pubertal development

|  | Youth - Caregiver | | |  | Youth - Teacher | | |  | Caregiver - Teacher | | |  |
| --- | --- | --- | --- | --- | --- | --- | --- | --- | --- | --- | --- | --- |
|  | INT | INA | EXT |  | INT | INA | EXT |  | INT | INA | EXT |  |
|  | (1) | (2) | (3) |  | (4) | (5) | (6) |  | (7) | (8) | (9) |  |
|  |  |  |  |  |  |  |  |  |  |  |  |  |
| Youth's characteristics | | | | | | | | | | | |  |
| Age (in years) | 0.133*** | 0.231*** | 0.105*** |  | 0.238*** | 0.309*** | 0.231*** |  | 0.087*** | 0.078** | 0.126*** |  |
|  | (0.012) | (0.015) | (0.011) |  | (0.026) | (0.030) | (0.020) |  | (0.023) | (0.026) | (0.018) |  |
| Sex at birth  (girl = 1) | 0.500*** | 0.857*** | 0.368*** |  | 0.461*** | 1.447*** | 0.588*** |  | 0.112* | 0.722*** | 0.309*** |  |
|  | (0.038) | (0.048) | (0.036) |  | (0.060) | (0.073) | (0.051) |  | (0.054) | (0.063) | (0.047) |  |
| Race  (non-white = 1) | 0.131* | 0.129t | 0.096t |  | -0.055 | -0.028 | -0.147* |  | -0.246*** | -0.192* | -0.195** |  |
|  | (0.054) | (0.069) | (0.051) |  | (0.080) | (0.098) | (0.068) |  | (0.071) | (0.084) | (0.062) |  |
| Pubertal  development | 0.049* | 0.035 | 0.011 |  | 0.036 | 0.012 | -0.059* |  | -0.025 | -0.028 | -0.080** |  |
|  | (0.021) | (0.026) | (0.019) |  | (0.034) | (0.041) | (0.028) |  | (0.030) | (0.036) | (0.026) |  |
| Immigrant (born  outside US = 1) | 0.150 | -0.353* | -0.151 |  | -0.032 | -0.242 | 0.243 |  | 0.033 | 0.300 | 0.399** |  |
|  | (0.115) | (0.148) | (0.110) |  | (0.188) | (0.233) | (0.161) |  | (0.170) | (0.199) | (0.147) |  |
| Caregiver's characteristics | | | | | | | | | | | |  |
| Age (in years) | -0.001 | -0.009* | -0.005t |  | -0.010* | -0.008 | -0.001 |  | -0.009* | 0.000 | -0.000 |  |
|  | (0.003) | (0.004) | (0.003) |  | (0.005) | (0.006) | (0.004) |  | (0.004) | (0.005) | (0.004) |  |
| Sex at birth  (female = 1) | -0.292*** | -0.197* | -0.219*** |  | -0.096 | -0.215t | -0.064 |  | 0.243** | -0.026 | 0.156* |  |
|  | (0.061) | (0.078) | (0.058) |  | (0.095) | (0.116) | (0.080) |  | (0.085) | (0.100) | (0.074) |  |
| Race  (non-white = 1) | 0.148* | -0.059 | 0.179** |  | -0.085 | -0.552*** | -0.412*** |  | -0.228** | -0.562*** | -0.610*** |  |
|  | (0.059) | (0.075) | (0.056) |  | (0.098) | (0.119) | (0.082) |  | (0.087) | (0.102) | (0.075) |  |
| Immigrant (born  outside US = 1) | 0.147* | 0.076 | 0.020 |  | 0.285** | 0.248* | 0.289*** |  | 0.137t | 0.348*** | 0.259*** |  |
|  | (0.057) | (0.073) | (0.054) |  | (0.091) | (0.111) | (0.076) |  | (0.081) | (0.095) | (0.070) |  |
| Depressive  symptoms | -0.378*** | -0.394*** | -0.334*** |  | 0.038 | 0.097** | 0.032 |  | 0.434*** | 0.515*** | 0.335*** |  |
|  | (0.020) | (0.026) | (0.019) |  | (0.031) | (0.037) | (0.026) |  | (0.027) | (0.032) | (0.024) |  |
|  |  |  |  |  |  |  |  |  |  |  |  |  |
|  |  |  |  |  |  |  |  |  |  |  |  |  |
| (Continues in the next page) | | | | | | | | | | | |  |
|  |  |  |  |  |  |  |  |  |  |  |  |  |
|  |  |  |  |  |  |  |  |  |  |  |  |  |
| Education level |  |  |  |  |  |  |  |  |  |  |  |  |
| Some College | -0.047 | 0.021 | -0.041 |  | 0.245* | 0.404** | 0.175t |  | 0.334** | 0.476*** | 0.269** |  |
|  | (0.070) | (0.090) | (0.067) |  | (0.114) | (0.140) | (0.097) |  | (0.102) | (0.120) | (0.089) |  |
| Associate  Degree | -0.094 | -0.089 | -0.132t |  | 0.233t | 0.372* | 0.112 |  | 0.294** | 0.500*** | 0.242** |  |
|  | (0.074) | (0.096) | (0.071) |  | (0.120) | (0.146) | (0.101) |  | (0.107) | (0.125) | (0.093) |  |
| College | -0.342*** | -0.273** | -0.176** |  | 0.390*** | 0.634*** | 0.398*** |  | 0.714*** | 0.907*** | 0.556*** |  |
|  | (0.068) | (0.087) | (0.065) |  | (0.108) | (0.132) | (0.091) |  | (0.096) | (0.113) | (0.084) |  |
| Masters or more | -0.414*** | -0.260** | -0.139* |  | 0.434*** | 0.907*** | 0.326*** |  | 0.811*** | 1.134*** | 0.478*** |  |
|  | (0.071) | (0.091) | (0.068) |  | (0.112) | (0.137) | (0.095) |  | (0.100) | (0.117) | (0.087) |  |
| Social environments | | | | | | | | | | | |  |
| Parental warmth | -0.134*** | -0.119*** | -0.024 |  | -0.034 | -0.114** | -0.051t |  | 0.096*** | 0.008 | -0.065** |  |
|  | (0.019) | (0.024) | (0.018) |  | (0.031) | (0.038) | (0.026) |  | (0.028) | (0.032) | (0.024) |  |
| Prosocial school  environment | -0.056** | -0.175*** | -0.121*** |  | -0.110*** | -0.176*** | -0.029 |  | -0.012 | 0.011 | 0.103*** |  |
|  | (0.018) | (0.023) | (0.017) |  | (0.031) | (0.037) | (0.026) |  | (0.027) | (0.032) | (0.023) |  |
| Family conflict | 0.155*** | 0.208*** | 0.219*** |  | 0.128*** | 0.205*** | 0.254*** |  | -0.089*** | -0.028 | 0.004 |  |
|  | (0.018) | (0.023) | (0.017) |  | (0.030) | (0.036) | (0.025) |  | (0.027) | (0.031) | (0.023) |  |
| Neighborhood  deprivation | 0.049t | 0.027 | 0.014 |  | -0.007 | 0.039 | -0.017 |  | -0.023 | 0.040 | -0.007 |  |
|  | (0.027) | (0.031) | (0.023) |  | (0.039) | (0.046) | (0.032) |  | (0.033) | (0.038) | (0.029) |  |
|  |  |  |  |  |  |  |  |  |  |  |  |  |
|  |  |  |  |  |  |  |  |  |  |  |  |  |
| Ave. outcome | 0.391 | 1.196 | 0.400 |  | 0.298 | 0.854 | 0.887 |  | -0.0221 | -0.253 | 0.415 |  |
| N research sites | 21 | 21 | 21 |  | 21 | 21 | 21 |  | 21 | 21 | 21 |  |
| N families | 8,847 | 8,812 | 8,847 |  | 6,288 | 6,174 | 6,284 |  | 6,445 | 6,479 | 6,472 |  |
| N person-wave dyads | 28,172 | 27,240 | 27,988 |  | 11,052 | 10,729 | 11,042 |  | 11,519 | 11,570 | 11,571 |  |
|  |  |  |  |  |  |  |  |  |  |  |  |  |
|  |  |  |  |  |  |  |  |  |  |  |  |  |
| INT: Internalizing; INA: Inattention; EXT: Externalizing; Ave.: Average; N: Number | | | | | | | | | | | |  |
| **Notes**: Each column represents an independent model. All models were fitted using multilevel mixed-effects linear regression and the estimations were performed by maximum likelihood using Stata 19 and the command MIXED. *** p<0.001, ** p<0.01, * p<0.05, t <0.10. | | | | | | | | | | | |  |
|  |  |  |  |  |  |  |  |  |  |  |  |  |

**Table S24.** Predictors of reporting differences, using data only from 1-year follow-up

|  | Youth - Caregiver | | |  | Youth - Teacher | | |  | Caregiver - Teacher | | |
| --- | --- | --- | --- | --- | --- | --- | --- | --- | --- | --- | --- |
|  | INT | INA | EXT |  | INT | INA | EXT |  | INT | INA | EXT |
|  | (1) | (2) | (3) |  | (4) | (5) | (6) |  | (7) | (8) | (9) |
|  |  |  |  |  |  |  |  |  |  |  |  |
| Youth's characteristics | | | | | | | | | | | |
| Age (in years) | -0.003 | 0.133** | 0.116** |  | 0.088 | 0.173* | 0.166** |  | 0.132* | 0.060 | 0.102* |
|  | (0.038) | (0.048) | (0.036) |  | (0.058) | (0.072) | (0.051) |  | (0.052) | (0.061) | (0.046) |
| Sex at birth  (girl = 1) | 0.180*** | 0.477*** | 0.171*** |  | 0.270*** | 1.395*** | 0.499*** |  | 0.084 | 0.796*** | 0.305*** |
|  | (0.049) | (0.062) | (0.047) |  | (0.074) | (0.093) | (0.065) |  | (0.067) | (0.079) | (0.059) |
| Race  (non-white = 1) | 0.181** | 0.150t | 0.034 |  | 0.001 | -0.042 | -0.200* |  | -0.139 | -0.201t | -0.214** |
|  | (0.068) | (0.085) | (0.066) |  | (0.104) | (0.127) | (0.090) |  | (0.092) | (0.109) | (0.082) |
| Pubertal  development | 0.108*** | -0.027 | 0.036 |  | 0.069t | 0.065 | 0.065t |  | -0.017 | 0.051 | -0.039 |
|  | (0.025) | (0.032) | (0.024) |  | (0.039) | (0.049) | (0.035) |  | (0.035) | (0.042) | (0.031) |
| Immigrant (born  outside US = 1) | 0.181 | -0.331t | -0.237t |  | -0.030 | 0.078 | 0.220 |  | 0.059 | 0.598* | 0.580** |
|  | (0.148) | (0.188) | (0.144) |  | (0.256) | (0.324) | (0.228) |  | (0.233) | (0.275) | (0.205) |
| Caregiver's characteristics | | | | | | | | | | | |
| Age (in years) | 0.001 | -0.005 | -0.003 |  | -0.009 | -0.007 | 0.002 |  | -0.006 | -0.001 | 0.003 |
|  | (0.004) | (0.005) | (0.004) |  | (0.006) | (0.008) | (0.006) |  | (0.006) | (0.007) | (0.005) |
| Sex at birth  (female = 1) | -0.246** | -0.177t | -0.271*** |  | -0.151 | -0.072 | -0.101 |  | 0.112 | 0.040 | 0.149 |
|  | (0.079) | (0.101) | (0.077) |  | (0.124) | (0.155) | (0.108) |  | (0.111) | (0.132) | (0.098) |
| Race  (non-white = 1) | 0.236** | -0.005 | 0.236** |  | -0.095 | -0.581*** | -0.469*** |  | -0.384*** | -0.650*** | -0.734*** |
|  | (0.076) | (0.095) | (0.073) |  | (0.127) | (0.156) | (0.110) |  | (0.112) | (0.132) | (0.099) |
| Immigrant (born  outside US = 1) | 0.183* | 0.019 | -0.032 |  | 0.242* | 0.344* | 0.247* |  | 0.120 | 0.425*** | 0.293** |
|  | (0.073) | (0.092) | (0.070) |  | (0.120) | (0.148) | (0.104) |  | (0.107) | (0.126) | (0.094) |
| Depressive  symptoms | -0.474*** | -0.456*** | -0.395*** |  | 0.013 | 0.079 | 0.059t |  | 0.460*** | 0.567*** | 0.396*** |
|  | (0.025) | (0.032) | (0.025) |  | (0.039) | (0.049) | (0.034) |  | (0.035) | (0.042) | (0.031) |
|  |  |  |  |  |  |  |  |  |  |  |  |
|  |  |  |  |  |  |  |  |  |  |  |  |
| (Continues in the next page) | | | | | | | | | | | |
|  |  |  |  |  |  |  |  |  |  |  |  |
|  |  |  |  |  |  |  |  |  |  |  |  |
| Education level |  |  |  |  |  |  |  |  |  |  |  |
| Some College | -0.116 | -0.047 | -0.130 |  | 0.261t | 0.390* | 0.099 |  | 0.303* | 0.477** | 0.245* |
|  | (0.090) | (0.113) | (0.086) |  | (0.148) | (0.184) | (0.129) |  | (0.133) | (0.158) | (0.118) |
| Associate  Degree | -0.180t | -0.116 | -0.191* |  | 0.227 | 0.300 | 0.041 |  | 0.285* | 0.341* | 0.155 |
|  | (0.094) | (0.119) | (0.091) |  | (0.154) | (0.192) | (0.135) |  | (0.137) | (0.164) | (0.122) |
| College | -0.473*** | -0.343** | -0.192* |  | 0.378** | 0.547** | 0.348** |  | 0.832*** | 0.875*** | 0.570*** |
|  | (0.086) | (0.108) | (0.083) |  | (0.140) | (0.173) | (0.122) |  | (0.125) | (0.148) | (0.111) |
| Masters or more | -0.475*** | -0.287* | -0.186* |  | 0.437** | 0.760*** | 0.290* |  | 0.840*** | 1.032*** | 0.491*** |
|  | (0.090) | (0.113) | (0.087) |  | (0.145) | (0.179) | (0.127) |  | (0.129) | (0.153) | (0.115) |
| Social environments | | | | | | | | | | | |
| Parental warmth | -0.132*** | -0.117*** | -0.020 |  | -0.040 | -0.060 | -0.004 |  | 0.090* | 0.039 | -0.060t |
|  | (0.027) | (0.034) | (0.026) |  | (0.042) | (0.052) | (0.037) |  | (0.037) | (0.044) | (0.033) |
| Prosocial school  environment | -0.098*** | -0.199*** | -0.180*** |  | -0.135*** | -0.177*** | -0.061t |  | -0.006 | 0.017 | 0.129*** |
|  | (0.026) | (0.033) | (0.025) |  | (0.041) | (0.051) | (0.036) |  | (0.037) | (0.043) | (0.032) |
| Family conflict | 0.206*** | 0.289*** | 0.242*** |  | 0.153*** | 0.325*** | 0.336*** |  | -0.056 | 0.019 | 0.076* |
|  | (0.026) | (0.033) | (0.025) |  | (0.039) | (0.049) | (0.034) |  | (0.035) | (0.041) | (0.031) |
| Neighborhood  deprivation | 0.038 | 0.043 | 0.017 |  | 0.010 | 0.086 | -0.012 |  | 0.015 | 0.092t | 0.010 |
|  | (0.032) | (0.037) | (0.030) |  | (0.051) | (0.056) | (0.042) |  | (0.043) | (0.047) | (0.037) |
|  |  |  |  |  |  |  |  |  |  |  |  |
|  |  |  |  |  |  |  |  |  |  |  |  |
| Ave. outcome | 0.254 | 0.949 | 0.271 |  | 0.132 | 0.598 | 0.680 |  | -0.0525 | -0.315 | 0.312 |
| N research sites | 21 | 21 | 21 |  | 21 | 21 | 21 |  | 21 | 21 | 21 |
| N families | 8,163 | 7,874 | 8,097 |  | 4,571 | 4,411 | 4,535 |  | 4,776 | 4,775 | 4,789 |
| N youth dyads | 9,614 | 9,217 | 9,533 |  | 5,180 | 4,978 | 5,141 |  | 5,463 | 5,450 | 5,475 |
|  |  |  |  |  |  |  |  |  |  |  |  |
|  |  |  |  |  |  |  |  |  |  |  |  |
| INT: Internalizing; INA: Inattention; EXT: Externalizing; Ave.: Average; N: Number | | | | | | | | | | | |
| **Notes**: Each column represents an independent model. All models were fitted using multilevel mixed-effects linear regression and the estimations were performed by maximum likelihood using Stata 19 and the command MIXED. *** p<0.001, ** p<0.01, * p<0.05, t <0.10. | | | | | | | | | | | |

**Table S25.** Predictors of reporting differences, using data only from 2-year follow-up

|  | Youth - Caregiver | | |  | Youth - Teacher | | |  | Caregiver - Teacher | | |
| --- | --- | --- | --- | --- | --- | --- | --- | --- | --- | --- | --- |
|  | INT | INA | EXT |  | INT | INA | EXT |  | INT | INA | EXT |
|  | (1) | (2) | (3) |  | (4) | (5) | (6) |  | (7) | (8) | (9) |
|  |  |  |  |  |  |  |  |  |  |  |  |
| Youth's characteristics | | | | | | | | | | | |
| Age (in years) | 0.057 | 0.215*** | 0.100** |  | 0.125t | 0.176* | 0.111* |  | 0.152* | -0.027 | 0.133** |
|  | (0.037) | (0.046) | (0.034) |  | (0.068) | (0.081) | (0.054) |  | (0.061) | (0.070) | (0.049) |
| Sex at birth  (girl = 1) | 0.547*** | 0.837*** | 0.408*** |  | 0.537*** | 1.474*** | 0.530*** |  | 0.065 | 0.657*** | 0.128* |
|  | (0.049) | (0.061) | (0.046) |  | (0.088) | (0.105) | (0.070) |  | (0.080) | (0.091) | (0.064) |
| Race  (non-white = 1) | 0.092 | 0.127 | 0.087 |  | 0.079 | -0.010 | -0.067 |  | -0.099 | -0.123 | -0.181* |
|  | (0.069) | (0.086) | (0.064) |  | (0.121) | (0.145) | (0.096) |  | (0.109) | (0.124) | (0.089) |
| Pubertal  development | 0.095*** | 0.028 | -0.008 |  | 0.075 | 0.003 | -0.013 |  | -0.074t | -0.009 | -0.004 |
|  | (0.025) | (0.031) | (0.023) |  | (0.046) | (0.055) | (0.036) |  | (0.041) | (0.047) | (0.033) |
| Immigrant (born  outside US = 1) | 0.157 | -0.350t | 0.014 |  | 0.007 | -0.497 | 0.242 |  | 0.108 | 0.026 | 0.147 |
|  | (0.150) | (0.189) | (0.143) |  | (0.287) | (0.347) | (0.228) |  | (0.258) | (0.299) | (0.207) |
| Caregiver's characteristics | | | | | | | | | | | |
| Age (in years) | 0.003 | -0.012* | -0.004 |  | -0.006 | -0.007 | 0.000 |  | -0.007 | 0.008 | -0.000 |
|  | (0.004) | (0.005) | (0.004) |  | (0.008) | (0.009) | (0.006) |  | (0.007) | (0.008) | (0.006) |
| Sex at birth  (female = 1) | -0.292*** | -0.268** | -0.219** |  | -0.148 | -0.305t | -0.061 |  | 0.255t | -0.073 | 0.181t |
|  | (0.080) | (0.100) | (0.075) |  | (0.145) | (0.173) | (0.114) |  | (0.131) | (0.149) | (0.105) |
| Race  (non-white = 1) | 0.198** | -0.030 | 0.239*** |  | -0.121 | -0.367* | -0.477*** |  | -0.292* | -0.540*** | -0.655*** |
|  | (0.076) | (0.095) | (0.071) |  | (0.152) | (0.183) | (0.119) |  | (0.136) | (0.156) | (0.110) |
| Immigrant (born  outside US = 1) | 0.092 | 0.049 | -0.011 |  | 0.323* | 0.108 | 0.396*** |  | 0.149 | 0.294* | 0.344*** |
|  | (0.073) | (0.092) | (0.069) |  | (0.140) | (0.169) | (0.110) |  | (0.126) | (0.145) | (0.102) |
| Depressive  symptoms | -0.345*** | -0.377*** | -0.321*** |  | 0.057 | 0.037 | -0.022 |  | 0.460*** | 0.432*** | 0.289*** |
|  | (0.026) | (0.032) | (0.024) |  | (0.048) | (0.056) | (0.037) |  | (0.043) | (0.049) | (0.035) |
|  |  |  |  |  |  |  |  |  |  |  |  |
|  |  |  |  |  |  |  |  |  |  |  |  |
| (Continues in the next page) | | | | | | | | | | | |
|  |  |  |  |  |  |  |  |  |  |  |  |
|  |  |  |  |  |  |  |  |  |  |  |  |
| Education level |  |  |  |  |  |  |  |  |  |  |  |
| Some College | -0.105 | 0.108 | -0.056 |  | 0.361* | 0.563** | 0.141 |  | 0.626*** | 0.575** | 0.134 |
|  | (0.090) | (0.112) | (0.085) |  | (0.181) | (0.216) | (0.142) |  | (0.162) | (0.186) | (0.131) |
| Associate  Degree | -0.006 | -0.023 | -0.136 |  | 0.450* | 0.702** | 0.239 |  | 0.483** | 0.827*** | 0.331* |
|  | (0.095) | (0.119) | (0.089) |  | (0.187) | (0.225) | (0.147) |  | (0.168) | (0.194) | (0.136) |
| College | -0.380*** | -0.221* | -0.188* |  | 0.486** | 1.011*** | 0.507*** |  | 0.841*** | 1.084*** | 0.496*** |
|  | (0.086) | (0.109) | (0.081) |  | (0.167) | (0.201) | (0.132) |  | (0.150) | (0.173) | (0.122) |
| Masters or more | -0.451*** | -0.257* | -0.138 |  | 0.457** | 1.060*** | 0.359** |  | 0.917*** | 1.266*** | 0.359** |
|  | (0.091) | (0.113) | (0.085) |  | (0.172) | (0.207) | (0.137) |  | (0.155) | (0.179) | (0.126) |
| Social environments | | | | | | | | | | | |
| Parental warmth | -0.116*** | -0.139*** | 0.006 |  | 0.046 | -0.082 | -0.004 |  | 0.113* | 0.027 | -0.018 |
|  | (0.027) | (0.034) | (0.025) |  | (0.051) | (0.061) | (0.039) |  | (0.045) | (0.051) | (0.036) |
| Prosocial school  environment | -0.078** | -0.201*** | -0.155*** |  | -0.102* | -0.196*** | -0.029 |  | 0.012 | 0.015 | 0.081* |
|  | (0.026) | (0.033) | (0.024) |  | (0.049) | (0.058) | (0.038) |  | (0.044) | (0.050) | (0.035) |
| Family conflict | 0.153*** | 0.187*** | 0.238*** |  | 0.151** | 0.144* | 0.229*** |  | -0.129** | -0.052 | -0.031 |
|  | (0.026) | (0.032) | (0.024) |  | (0.048) | (0.057) | (0.038) |  | (0.043) | (0.049) | (0.034) |
| Neighborhood  deprivation | 0.051 | 0.016 | 0.004 |  | -0.039 | 0.022 | -0.022 |  | -0.000 | 0.061 | 0.006 |
|  | (0.033) | (0.039) | (0.029) |  | (0.054) | (0.065) | (0.043) |  | (0.048) | (0.055) | (0.041) |
|  |  |  |  |  |  |  |  |  |  |  |  |
|  |  |  |  |  |  |  |  |  |  |  |  |
| Ave. outcome | 0.373 | 1.213 | 0.463 |  | 0.225 | 0.878 | 0.962 |  | -0.0998 | -0.286 | 0.438 |
| N research sites | 21 | 21 | 21 |  | 21 | 21 | 21 |  | 21 | 21 | 21 |
| N families | 8,077 | 7,831 | 8,037 |  | 3,411 | 3,337 | 3,412 |  | 3,518 | 3,556 | 3,537 |
| N youth dyads | 9,516 | 9,166 | 9,449 |  | 3,864 | 3,769 | 3,872 |  | 4,000 | 4,049 | 4,027 |
|  |  |  |  |  |  |  |  |  |  |  |  |
|  |  |  |  |  |  |  |  |  |  |  |  |
| INT: Internalizing; INA: Inattention; EXT: Externalizing; Ave.: Average; N: Number | | | | | | | | | | | |
| **Notes**: Each column represents an independent model. All models were fitted using multilevel mixed-effects linear regression and the estimations were performed by maximum likelihood using Stata 19 and the command MIXED. *** p<0.001, ** p<0.01, * p<0.05, t <0.10. | | | | | | | | | | | |

**Table S26.** Predictors of reporting differences, using data only from 3-year follow-up

|  | Youth - Caregiver | | |  | Youth - Teacher | | |  | Caregiver - Teacher | | |  |
| --- | --- | --- | --- | --- | --- | --- | --- | --- | --- | --- | --- | --- |
|  | INT | INA | EXT |  | INT | INA | EXT |  | INT | INA | EXT |  |
|  | (1) | (2) | (3) |  | (4) | (5) | (6) |  | (7) | (8) | (9) |  |
|  |  |  |  |  |  |  |  |  |  |  |  |  |
| Youth's characteristics | | | | | | | | | | | |  |
| Age (in years) | 0.188*** | 0.274*** | 0.156*** |  | 0.131 | 0.299** | 0.129t |  | -0.064 | -0.030 | -0.032 |  |
|  | (0.040) | (0.049) | (0.036) |  | (0.104) | (0.114) | (0.076) |  | (0.086) | (0.096) | (0.070) |  |
| Sex at birth  (girl = 1) | 0.827*** | 1.278*** | 0.587*** |  | 0.883*** | 1.505*** | 0.507*** |  | 0.180 | 0.393** | 0.149t |  |
|  | (0.051) | (0.064) | (0.047) |  | (0.132) | (0.147) | (0.098) |  | (0.111) | (0.124) | (0.090) |  |
| Race  (non-white = 1) | 0.102 | 0.061 | 0.174** |  | -0.510** | -0.063 | 0.008 |  | -0.739*** | -0.205 | -0.065 |  |
|  | (0.072) | (0.089) | (0.065) |  | (0.176) | (0.196) | (0.130) |  | (0.145) | (0.163) | (0.119) |  |
| Pubertal  development | 0.099*** | -0.009 | -0.010 |  | 0.109 | -0.072 | -0.037 |  | 0.006 | 0.017 | -0.044 |  |
|  | (0.026) | (0.033) | (0.024) |  | (0.069) | (0.076) | (0.050) |  | (0.056) | (0.062) | (0.045) |  |
| Immigrant (born  outside US = 1) | 0.055 | -0.296 | -0.181 |  | -0.095 | -0.634 | 0.403 |  | -0.091 | 0.211 | 0.450 |  |
|  | (0.157) | (0.198) | (0.144) |  | (0.434) | (0.497) | (0.328) |  | (0.365) | (0.408) | (0.295) |  |
| Caregiver's characteristics | | | | | | | | | | | |  |
| Age (in years) | -0.003 | -0.017** | -0.010* |  | -0.014 | -0.016 | -0.006 |  | -0.027** | -0.004 | -0.003 |  |
|  | (0.004) | (0.005) | (0.004) |  | (0.011) | (0.013) | (0.008) |  | (0.009) | (0.010) | (0.008) |  |
| Sex at birth  (female = 1) | -0.305*** | -0.256* | -0.148t |  | 0.030 | -0.169 | -0.154 |  | 0.379* | 0.136 | 0.057 |  |
|  | (0.084) | (0.105) | (0.077) |  | (0.218) | (0.243) | (0.162) |  | (0.185) | (0.205) | (0.150) |  |
| Race  (non-white = 1) | 0.029 | -0.112 | 0.015 |  | 0.170 | -0.669** | -0.412* |  | 0.180 | -0.504* | -0.487** |  |
|  | (0.081) | (0.100) | (0.073) |  | (0.222) | (0.249) | (0.165) |  | (0.184) | (0.207) | (0.151) |  |
| Immigrant (born  outside US = 1) | 0.198* | 0.186t | 0.147* |  | 0.283 | 0.012 | 0.096 |  | 0.244 | 0.165 | -0.037 |  |
|  | (0.078) | (0.096) | (0.070) |  | (0.198) | (0.221) | (0.147) |  | (0.165) | (0.186) | (0.135) |  |
| Depressive  symptoms | -0.339*** | -0.333*** | -0.313*** |  | 0.022 | 0.190* | 0.069 |  | 0.320*** | 0.481*** | 0.258*** |  |
|  | (0.027) | (0.034) | (0.025) |  | (0.069) | (0.078) | (0.051) |  | (0.058) | (0.065) | (0.048) |  |
|  |  |  |  |  |  |  |  |  |  |  |  |  |
|  |  |  |  |  |  |  |  |  |  |  |  |  |
| (Continues in the next page) | | | | | | | | | | | |  |
|  |  |  |  |  |  |  |  |  |  |  |  |  |
|  |  |  |  |  |  |  |  |  |  |  |  |  |
| Education level |  |  |  |  |  |  |  |  |  |  |  |  |
| Some College | 0.111 | 0.045 | 0.043 |  | -0.046 | 0.363 | 0.335t |  | -0.154 | 0.485* | 0.320t |  |
|  | (0.097) | (0.121) | (0.088) |  | (0.260) | (0.290) | (0.192) |  | (0.217) | (0.242) | (0.178) |  |
| Associate  Degree | -0.089 | -0.063 | -0.086 |  | -0.281 | 0.083 | 0.196 |  | -0.052 | 0.385 | 0.339t |  |
|  | (0.102) | (0.127) | (0.093) |  | (0.279) | (0.311) | (0.207) |  | (0.234) | (0.261) | (0.192) |  |
| College | -0.187* | -0.273* | -0.188* |  | 0.091 | 0.274 | 0.220 |  | 0.277 | 0.859*** | 0.567*** |  |
|  | (0.093) | (0.115) | (0.084) |  | (0.244) | (0.270) | (0.179) |  | (0.203) | (0.227) | (0.165) |  |
| Masters or more | -0.346*** | -0.295* | -0.145t |  | 0.295 | 0.918** | 0.327t |  | 0.639** | 1.232*** | 0.554** |  |
|  | (0.097) | (0.120) | (0.088) |  | (0.253) | (0.281) | (0.187) |  | (0.211) | (0.236) | (0.172) |  |
| Social environments | | | | | | | | | | | |  |
| Parental warmth | -0.137*** | -0.100** | -0.029 |  | -0.151* | -0.220** | -0.180*** |  | 0.084 | -0.065 | -0.128** |  |
|  | (0.028) | (0.035) | (0.026) |  | (0.073) | (0.081) | (0.054) |  | (0.061) | (0.067) | (0.049) |  |
| Prosocial school  environment | -0.009 | -0.185*** | -0.108*** |  | -0.056 | -0.145t | 0.022 |  | -0.082 | -0.015 | 0.084t |  |
|  | (0.027) | (0.034) | (0.025) |  | (0.071) | (0.079) | (0.053) |  | (0.059) | (0.066) | (0.048) |  |
| Family conflict | 0.118*** | 0.164*** | 0.158*** |  | 0.023 | 0.082 | 0.136** |  | -0.098t | -0.167** | -0.030 |  |
|  | (0.027) | (0.034) | (0.025) |  | (0.069) | (0.076) | (0.051) |  | (0.057) | (0.063) | (0.046) |  |
| Neighborhood  deprivation | 0.030 | 0.013 | 0.009 |  | -0.029 | -0.102 | -0.033 |  | -0.147* | -0.100 | -0.082 |  |
|  | (0.036) | (0.041) | (0.030) |  | (0.080) | (0.087) | (0.059) |  | (0.064) | (0.073) | (0.053) |  |
|  |  |  |  |  |  |  |  |  |  |  |  |  |
|  |  |  |  |  |  |  |  |  |  |  |  |  |
| Ave. outcome | 0.564 | 1.447 | 0.472 |  | 0.892 | 1.460 | 1.283 |  | 0.212 | -0.0322 | 0.657 |  |
| N research sites | 21 | 21 | 21 |  | 21 | 21 | 21 |  | 21 | 21 | 21 |  |
| N families | 7,577 | 7,444 | 7,557 |  | 1,736 | 1,722 | 1,756 |  | 1,768 | 1,784 | 1,780 |  |
| N youth dyads | 8,934 | 8,752 | 8,896 |  | 1,962 | 1,935 | 1,979 |  | 2,004 | 2,019 | 2,016 |  |
|  |  |  |  |  |  |  |  |  |  |  |  |  |
|  |  |  |  |  |  |  |  |  |  |  |  |  |
| INT: Internalizing; INA: Inattention; EXT: Externalizing; Ave.: Average; N: Number | | | | | | | | | | | |  |
| **Notes**: Each column represents an independent model. All models were fitted using multilevel mixed-effects linear regression and the estimations were performed by maximum likelihood using Stata 19 and the command MIXED. *** p<0.001, ** p<0.01, * p<0.05, t <0.10. | | | | | | | | | | | |  |
|  |  |  |  |  |  |  |  |  |  |  |  |  |

# **Appendix S9**: Alternative specifications for the model testing discrepancies over time

As a robustness check for our over-time analyses, we re-estimated the reporter-by-domain panel models using ABCD data-collection periods (1-year, 2-year, and 3-year follow-up) as the time metric instead of youth’s continuous age. This alternative specification is useful because participants span a non-trivial age range within each assessment wave, and period-based models summarize average differences across assessment occasions without imposing a particular functional form for age-related change. We fit the same multilevel mixed-effects framework as in the main manuscript, but replaced the age-based time trend with indicator variables for the 2-year and 3-year follow-ups (with the 1-year follow-up as the reference), along with reporter indicators (caregiver, teacher; youth as the reference) and reporter-by-period interactions (**Table S27** and **Figure S5**). To avoid collinearity and over-adjustment—given that age varies within each period and is strongly linked to wave—we did not additionally control for youth age in this period-based specification.

Results from the period-based model support the same conclusion as the age-based analyses in the main text: reporter discrepancies persist and tend to widen across follow-up waves. **Figure S5** shows that youth-reported symptoms increase from the 1-year to the 3-year follow-up across domains, with the clearest upward shift for inattention (and a similar pattern for the “*all symptoms*” index). In contrast, caregiver- and teacher-reported symptoms are consistently lower than youth reports at each wave and appear comparatively flatter over time – so the gap between youth and the other reporters becomes larger by the 3-year follow-up, especially for inattention. This pattern is also reflected in **Table S27**. Relative to youth reports at the 1-year follow-up, caregivers and teachers report substantially lower symptom levels across domains (all p < .001). The positive period coefficients indicate that youth-reported symptoms increase at later follow-ups (including from 1-year to 3-year across all domains), while the negative reporter-by-period interaction terms indicate that caregiver and teacher reports increase less than youth reports across waves (i.e., caregiver and teacher trajectories are flatter), yielding larger youth–caregiver and youth–teacher discrepancies at later follow-ups – most notably by the 3-year follow-up.

**Table S27.** Predictors of reporting differences at the domains x reporters-level data (using periods)

|  | Mental health domains | | | |  |
| --- | --- | --- | --- | --- | --- |
|  | INT | INA | EXT | ALL |  |
|  | (1) | (2) | (3) | (4) |  |
|  |  |  |  |  |  |
| Reporters | | | | |  |
| Caregivers | -0.252*** | -0.945*** | -0.385*** | -0.517*** |  |
|  | (0.025) | (0.030) | (0.021) | (0.017) |  |
| Teachers | -0.167*** | -0.621*** | -0.687*** | -0.482*** |  |
|  | (0.030) | (0.037) | (0.025) | (0.020) |  |
| Periods | | | | |  |
| Two-year follow-up | 0.061* | 0.152*** | 0.096*** | 0.103*** |  |
|  | (0.025) | (0.031) | (0.022) | (0.017) |  |
| Three-year follow-up | 0.284*** | 0.428*** | 0.119*** | 0.282*** |  |
|  | (0.026) | (0.032) | (0.022) | (0.017) |  |
| Reporters x periods | | | | |  |
| Caregivers x two-year follow-up | -0.129*** | -0.258*** | -0.178*** | -0.188*** |  |
|  | (0.035) | (0.043) | (0.030) | (0.024) |  |
| Caregivers x three-year follow-up | -0.316*** | -0.493*** | -0.182*** | -0.335*** |  |
|  | (0.036) | (0.044) | (0.030) | (0.024) |  |
| Teachers x two-year follow-up | -0.070 | -0.290*** | -0.285*** | -0.208*** |  |
|  | (0.045) | (0.055) | (0.038) | (0.030) |  |
| Teachers x three-year follow-up | -0.544*** | -0.798*** | -0.514*** | -0.619*** |  |
|  | (0.054) | (0.065) | (0.045) | (0.036) |  |
| Youth's characteristics | | | | |  |
| Sex at birth (girl = 1) | 0.365*** | -0.613*** | -0.169*** | -0.129*** |  |
|  | (0.024) | (0.032) | (0.021) | (0.019) |  |
| Race (non-white = 1) | 0.068t | 0.247*** | 0.099** | 0.129*** |  |
|  | (0.036) | (0.051) | (0.032) | (0.032) |  |
| Puberty | 0.090*** | 0.066*** | 0.047*** | 0.059*** |  |
|  | (0.011) | (0.015) | (0.010) | (0.009) |  |
| Immigrant (born outside US = 1) | 0.037 | 0.022 | 0.002 | 0.034 |  |
|  | (0.077) | (0.108) | (0.068) | (0.068) |  |
|  | | | | |  |
|  | | | | |  |
|  | | | | |  |
|  | | | | |  |
|  | | | | |  |
|  | | | | |  |
|  | | | | |  |
| (Continues in the next page) | | | | |  |
|  | | | | |  |
| Caregiver’s characteristics | | | | |  |
| Age (in years) | 0.002 | 0.004 | -0.002 | 0.001 |  |
|  | (0.002) | (0.003) | (0.002) | (0.002) |  |
| Sex at birth (female = 1) | -0.040 | 0.068 | 0.113** | 0.052 |  |
|  | (0.041) | (0.056) | (0.036) | (0.035) |  |
| Race (non-white = 1) | -0.246*** | -0.131* | -0.026 | -0.126*** |  |
|  | (0.039) | (0.055) | (0.035) | (0.035) |  |
| Immigrant (born outside US = 1) | -0.025 | -0.193*** | -0.133*** | -0.121*** |  |
|  | (0.038) | (0.054) | (0.034) | (0.034) |  |
| Depressive symptoms | 0.359*** | 0.451*** | 0.275*** | 0.361*** |  |
|  | (0.013) | (0.019) | (0.012) | (0.012) |  |
| Education level |  |  |  |  |  |
| Some College | 0.041 | 0.061 | -0.028 | 0.015 |  |
|  | (0.047) | (0.066) | (0.042) | (0.042) |  |
| Associate Degree | 0.057 | -0.002 | -0.009 | 0.009 |  |
|  | (0.050) | (0.070) | (0.044) | (0.044) |  |
| College | 0.019 | -0.170** | -0.149*** | -0.099* |  |
|  | (0.046) | (0.064) | (0.040) | (0.041) |  |
| Masters or more | 0.039 | -0.246*** | -0.172*** | -0.133** |  |
|  | (0.048) | (0.067) | (0.042) | (0.043) |  |
| Social environments | | | | |  |
| Parental warmth | -0.036** | -0.121*** | -0.104*** | -0.086*** |  |
|  | (0.012) | (0.016) | (0.011) | (0.010) |  |
| Prosocial school environment | -0.111*** | -0.133*** | -0.084*** | -0.101*** |  |
|  | (0.012) | (0.016) | (0.010) | (0.010) |  |
| Family conflict | 0.103*** | 0.219*** | 0.165*** | 0.152*** |  |
|  | (0.012) | (0.016) | (0.010) | (0.009) |  |
| Neighborhood deprivation | 0.019 | 0.065* | 0.057*** | 0.050** |  |
|  | (0.016) | (0.026) | (0.016) | (0.016) |  |
|  |  |  |  |  |  |
|  |  |  |  |  |  |
| Average outcome | 1.640 | 2.700 | 1.476 | 1.936 |  |
| Number of observations | 69,146 | 68,264 | 69,053 | 206,463 |  |
|  |  |  |  |  |  |
|  |  |  |  |  |  |
| INT: Internalizing; INA: Inattention; EXT: Externalizing; Ave.: Average; N: Number | | | | |  |
| **Notes**: Each column represents an independent model. All models were fitted using multilevel mixed-effects linear regression and the estimations were performed by maximum likelihood using Stata 19 and the command MIXED. *** p<0.001, ** p<0.01, * p<0.05, t <0.10. | | | | |  |
|  |  |  |  |  |  |
|  |  |  |  |  |  |

**Figure S5.** Mental health symptoms over periods


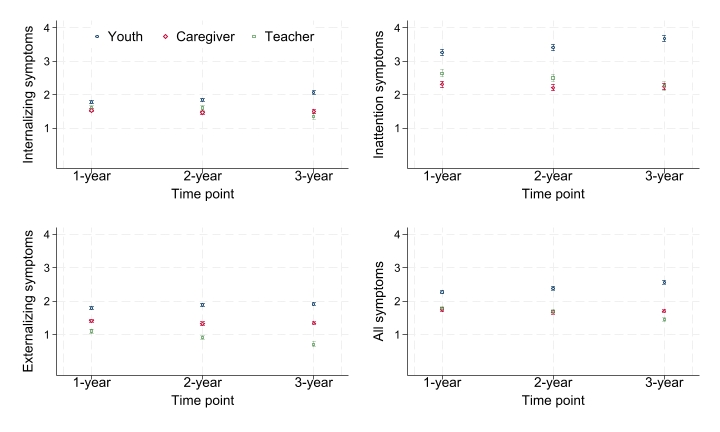


# **Appendix S10**: Sensitivity analysis results

Sensitivity analyses examined the robustness of our findings in several ways. First, we tested alternative operationalizations of discrepancies by substituting the outcome from continuous symptom ratings to (a) exact rating mismatches (**Tables S7-S9, and Figure S2 in Appendix S5**), (b) mismatches in symptom presence versus absence (**Tables S10-S12, and Figure S3 in Appendix S5**), and (c) absolute difference scores (i.e., discrepancy $[D]=\left| Reporter1 - Reporter2 \right|$) only for predictors of discrepancies; see **Table S13 in Appendix S5**). Second, we (a) assessed missingness patterns (**Tables S14- S15 in Appendix S6**) and (b) we refit the main models using multiple imputation for missing data (**Tables S16-S18 and Figure S4 in Appendix S6**). Third, we applied false-discovery-rate and Bonferroni corrections to address multiple testing (**Tables S19-S20 in Appendix S7**). Fourth, we re-estimated the predictor models using alternative specifications using (a) standardized values for the discrepancies to report standardized estimates (**Table S21 in Appendix S8)**, (b) time-varying caregiver age and depressive symptoms as well as social-context variables (instead of baseline-only covariates: see **Table S22 in Appendix S8)**, (c) parental reports of pubertal development (instead of youth reports: see **Table S23 in Appendix S8)**, and (d) data from each of the follow-ups to replicate the main analysis separately for 1-year, 2-year, and 3-year follow-ups (**Tables S24-S26 in Appendix S8**). Lastly, we explored discrepancies over time (aim 3) by including interactions between informant and assessment period (instead of youth’s age; **Table S27 and Figure S5 in Appendix S9**).

Results from these sensitivity analyses were highly consistent with the reported results. First, the mean-level discrepancies used for the main analyses correspond to between 20-45% mismatches in symptom reporters, with the highest levels for inattention. Second, most covariates, including sex at birth, pubertal development, caregiver’s depressive symptoms, and social environments, are consistently associated with greater discrepancies. Third, similar to mean-level, mismatches in discrepancies tend to increase over time.

# **References**

Achenbach, T. M., & Rescorla, L. A. (2001). *Manual for the ASEBA school-age forms & profiles*. University of Vermont Research Centre for Children, Youth and Families.

Beltz, A. M., Pham, H., Smith, T., Hidalgo‐Lopez, E., Becker, H., Portengen, C. M., Heitzeg, M. M., Kaplan, C., & Berenbaum, S. A. (2025). Research Review: On the (mis) use of puberty data in the ABCD Study®–a systematic review, problem illustration, and path forward. *Journal of Child Psychology and Psychiatry*.

Cheng, T. W., Magis-Weinberg, L., Guazzelli Williamson, V., Ladouceur, C. D., Whittle, S. L., Herting, M. M., Uban, K. A., Byrne, M. L., Barendse, M. E., & Shirtcliff, E. A. (2021). A Researcher’s Guide to the Measurement and Modeling of Puberty in the ABCD Study® at Baseline. *Frontiers in Endocrinology*, *12*, 608575. https://doi.org/10.3389/fendo.2021.608575

Hyman, S. E. (2010). The diagnosis of mental disorders: The problem of reification. *Annual Review of Clinical Psychology*, *6*, 155–179. https://doi.org/10.1146/annurev.clinpsy.3.022806.091532

Karcher, N. R., & Barch, D. M. (2020). The ABCD study: Understanding the development of risk for mental and physical health outcomes. *Neuropsychopharmacology*, *46*(1), 131–142. https://doi.org/10.1038/s41386-020-0736-6

Michelini, G., Barch, D. M., Tian, Y., Watson, D., Klein, D. N., & Kotov, R. (2019). Delineating and validating higher-order dimensions of psychopathology in the Adolescent Brain Cognitive Development (ABCD) study. *Translational Psychiatry*, *9*, Article 261. https://doi.org/10.1038/s41398-019-0593-4

Newson, J. J., Pastukh, V., & Thiagarajan, T. C. (2021). Poor separation of clinical symptom profiles by DSM-5 disorder criteria. *Frontiers in Psychiatry*, *12*, 775762. https://doi.org/10.3389/fpsyt.2021.775762

Pedersen, M. L., Jozefiak, T., Sund, A. M., Holen, S., Neumer, S.-P., Martinsen, K. D., Rasmussen, L. M. P., Patras, J., & Lydersen, S. (2021). Psychometric properties of the Brief Problem Monitor (BPM) in children with internalizing symptoms: Examining baseline data from a national randomized controlled intervention study. *BMC Psychology*, *9*, 1–12. https://doi.org/10.1186/s40359-021-00689-1

Piper, B. J., Gray, H. M., Raber, J., & Birkett, M. A. (2014). Reliability and validity of brief problem monitor, an abbreviated form of the child behavior checklist. *Psychiatry and Clinical Neurosciences*, *68*(10), 759–767. https://doi.org/10.1111/pcn.12188

1. When wave-specific values were unavailable, we constructed time-varying covariates using within-person linear interpolation between adjacent observed waves; because caregiver depressive symptoms were not measured at the 3-year follow-up, we carried forward the 2-year value for that wave. [↑](#footnote-ref-1)
